# Multi-center, pragmatic, cluster-randomized, controlled trial of standardized peritoneal dialysis (PD) training versus usual care on PD-related infections (the TEACH-PD trial): trial protocol

**DOI:** 10.1186/s13063-023-07715-0

**Published:** 2023-11-14

**Authors:** Josephine S. F. Chow, Neil Boudville, Yeoungjee Cho, Suetonia Palmer, Elaine M. Pascoe, Carmel M. Hawley, Donna M. Reidlinger, Laura E. Hickey, Ruth Stastny, Andrea Valks, Liza Vergara, Ramya Movva, Charani Kiriwandeniya, Hayley Candler, Gabor Mihala, Bernadette Buisman, Keri-Lu Equinox, Ana E. Figueiredo, Trudi Fuge, Kirsten Howard, Martin Howell, Allison Jaure, Matthew D. Jose, Anna Lee, Susana S. Miguel, Jo-anne Moodie, Thu T. Nguyen, Geraldine Pinlac, Annie Reynolds, Walaa W. M. Saweirs, Genevieve Z. Steiner-Lim, Bronwen TeWhare, Melinda Tomlins, Megan Upjohn, David Voss, Rachael C. Walker, Joanne Wilson, David W. Johnson

**Affiliations:** 1 South Western Sydney Local Health District, Liverpool, NSW Australia; 2 Ingham Institute for Applied Medical Research, Liverpool, NSW Australia; 3 University of New South Wales, Kennington, NSW Australia; 4 Western Sydney University, Sydney, NSW Australia; 5 University of Tasmania, Hobart, TAS Australia; 6 Medical School, University of Western Australia, Crawley, WA Australia; 7 Department of Renal Medicine, Sir Charles Gairdner Hospital, Nedlands, WA Australia; 8 Department of Kidney and Transplant Service, Princess Alexandra Hospital, Brisbane, QLD Australia; 9 Australasian Kidney Trials Network, Centre for Health Services Research, The University of Queensland, Brisbane, QLD Australia; 10 Department of Medicine, University of Otago, Christchurch, New Zealand; 11 Te Whatu Ora Health New Zealand, Hamilton, New Zealand; 12 Translational Research Institute, Brisbane, QLD Australia; 13 Metro South Kidney and Transplant Service, Princess Alexandra Hospital, Brisbane, QLD Australia; 14 Centre for Health Services Research, The University of Queensland, Brisbane, QLD Australia; 15 Health New Zealand, Te Whatu Ora Te Tai Tokerau, Hamilton, New Zealand; 16 University of Auckland, Te Tai Tokerau Northtec, Auckland, New Zealand; 17 Cairns Hospital, Cairns, QLD Australia; 18 School of Nursing, Escola de Ciências da Saúde E da Vida, Pontifícia Universidade Católica Do Rio Grande Do Sul, Porto Alegre, Brazil; 19 Wollongong, Australia; 20 Sydney School of Public Health, The University of Sydney, Sydney, NSW Australia; 21 Menzies Centre for Health Policy and Economics, Faculty of Medicine and Health, University of Sydney, Sydney, NSW Australia; 22 Renal Unit, Royal Hobart Hospital, Hobart, TAS Australia; 23 Shoalhaven, Australia; 24 Royal Melbourne Hospital, Parkville, VIC Australia; 25 Health New Zealand, Te Whatu Ora Te Toka Tumai, Hamilton, New Zealand; 26 Health New Zealand, Te Whatu Ora Te Matua a Māui Hawkes Bay, Hamilton, New Zealand; 27 NICM Health Research Institute, Western Sydney University Sydney, Campbelltown, NSW Australia; 28 Health New Zealand, Te Whatu Ora Taranaki, Hamilton, New Zealand; 29 Department of Nephrology, Hunter New England Local Health District, New Lambton, NSW Australia; 30 Health New Zealand, Te Whatu Ora Counties Manukau, Hamilton, New Zealand; 31 Te Pukenga Eastern Institute of Technology, Christchurch, New Zealand

**Keywords:** Cluster randomized controlled trial, Competency assessment, Cost-effectiveness, Outcomes, Patient education, Peritoneal dialysis, Peritonitis, Standardized training

## Abstract

**Background:**

Peritoneal dialysis (PD)-related infections, such as peritonitis, exit site, and tunnel infections, substantially impair the sustainability of PD. Accordingly, PD-related infection is the top-priority research outcome for patients and caregivers. While PD nurse trainers teach patients to perform their own PD, PD training curricula are not standardized or informed by an evidentiary base and may offer a potential approach to prevent PD infections. The Targeted Education ApproaCH to improve Peritoneal Dialysis outcomes (TEACH-PD) trial evaluates whether a standardized training curriculum for PD nurse trainers and incident PD patients based on the International Society for Peritoneal Dialysis (ISPD) guidelines reduces PD-related infections compared to usual training practices.

**Methods:**

The TEACH-PD trial is a registry-based, pragmatic, open-label, multi-center, binational, cluster-randomized controlled trial. TEACH-PD will recruit adults aged 18 years or older who have not previously undergone PD training at 42 PD treatment units (clusters) in Australia and New Zealand (ANZ) between July 2019 and June 2023. Clusters will be randomized 1:1 to standardized TEACH-PD training curriculum or usual training practice. The primary trial outcome is the time to the first occurrence of any PD-related infection (exit site infection, tunnel infection, or peritonitis). The secondary trial outcomes are the individual components of the primary outcome, infection-associated catheter removal, transfer to hemodialysis (greater than 30 days and 180 days), quality of life, hospitalization, all-cause death, a composite of transfer to hemodialysis or all-cause death, and cost-effectiveness. Participants are followed for a minimum of 12 months with a targeted average follow-up period of 2 years. Participant and outcome data are collected from the ANZ Dialysis and Transplant Registry (ANZDATA) and the New Zealand Peritoneal Dialysis (NZPD) Registry. This protocol follows the Standard Protocol Items: Recommendations for Interventional Trials (SPIRIT) guidelines.

**Discussion:**

TEACH-PD is a registry-based, cluster-randomized pragmatic trial that aims to provide high-certainty evidence about whether an ISPD guideline-informed standardized PD training curriculum for PD nurse trainers and adult patients prevents PD-related infections.

**Trial registration:**

ClinicalTrials.gov NCT03816111. Registered on 24 January 2019.

**Supplementary Information:**

The online version contains supplementary material available at 10.1186/s13063-023-07715-0.

## Administrative Information

Note: the numbers in curly brackets in this protocol refer to a Standard Protocol Items: Recommendations for Interventional Trials (SPIRIT) checklist item numbers [1, 2]. The order of the items has been modified to group similar items (see http://www.equator-network.org/reporting-guidelines/spirit-2013-statement-defining-standard-protocol-items-for-clinical-trials/).

**Table:** 

Title {1}	Multi-center, pragmatic, cluster-randomized, controlled trial of standardized Peritoneal Dialysis (PD) training versus usual care on PD-related infections (The TEACH-PD Trial): Trial Protocol
Trial registration {2a and 2b}	ClinicalTrials.gov NCT03816111. Registered on 24^th^ January 2019.
Protocol version {3}	Version 1.0, 30 January 2019
Funding {4}	1. MRFF Clinical Trials Activity: Rare Cancers, Rare Diseases and Unmet Need Grant Opportunity (APP1170238) 2. National Health & Medical Research Council (NHMRC) BEAT-CKD Program Grant (APP1092957) 3. Health Research Council of New Zealand grant 19/290 4. Funding support from Metro South Health Research Support Scheme Research Fund—Health System and Health Economics Project Grant 5. Funding support from Queensland Health 6. South Western Sydney Research Small Grant Scheme 7. Funding support from the International Society for Peritoneal Dialysis 8. Supported by the Translational Research Institute Australia 9. Supported by Amgen 10. Supported by Baxter Healthcare
Author details {5a}	1. South Western Sydney Local Health District, Liverpool, New South Wales, Australia 2. Ingham Institute for Applied Medical Research, Liverpool, New South Wales, Australia 3. University of New South Wales, Kennington, New South Wales, Australia 4. Western Sydney University, Sydney, New South Wales, Australia 5. University of Tasmania, Hobart, Tasmania, Australia 6. Medical School, University of Western Australia, Crawley, Western Australia, Australia 7. Department of Renal Medicine, Sir Charles Gairdner Hospital, Nedlands, Western Australia, Australia 8. Department of Kidney and Transplant Service, Princess Alexandra Hospital, Brisbane, Queensland, Australia 9. Australasian Kidney Trials Network, Centre for Health Services Research, The University of Queensland, Brisbane, Queensland, Australia 10. Department of Medicine, University of Otago, Christchurch, Christchurch, New Zealand 11. Te Whatu Ora Health New Zealand, New Zealand 12. Translational Research Institute, Brisbane, Queensland, Australia 13. Metro South Kidney and Transplant Service, Princess Alexandra Hospital, Brisbane, Queensland, Australia 14. Centre for Health Services Research, The University of Queensland, Brisbane, Queensland, Australia 15. Te Whatu Ora Te Tai Tokerau, Health New Zealand, New Zealand 16. University of Auckland, Te Tai Tokerau Northtec, New Zealand 17. Cairns Hospital, Cairns, Queensland, Australia 18. School of Nursing, Escola de Ciências da Saúde e da Vida, Pontifícia Universidade Católica do Rio Grande do Sul, Porto Alegre, Brazil 19. Sydney School of Public Health, The University of Sydney, Sydney, New South Wales, Australia 20. Menzies Centre for Health Policy and Economics, Faculty of Medicine and Health, University of Sydney, Sydney, New South Wales, Australia 21. Renal Unit, Royal Hobart Hospital, Hobart, Tasmania, Australia 22. Royal Melbourne Hospital, Parkville, Victoria, Australia 23.Te Whatu Ora Te Toka Tumai, Health New Zealand, New Zealand 24.Te Whatu Ora Te Matua a Māui Hawkes Bay, Health New Zealand, New Zealand 25. NICM Health Research Institute, Western Sydney University Sydney, Campbelltown, New South Wales, Australia 26. Te Whatu Ora Taranaki, Health New Zealand, New Zealand 27. Department of Nephrology, Hunter New England Local Health District, New Lambton, New South Wales, Australia 28. Te Whatu Ora Counties Manukau, Health New Zealand, New Zealand 29. Te Pukenga Eastern Institute of Technology, New Zealand
Name and contact information for the trial sponsor {5b}	The University of Queensland acting through the Australasian Kidney Trials Network (AKTN) Email: aktn@uq.edu.au
Role of sponsor {5c}	The sponsor is the coordinating centre for the trial and is involved in overall study activities including study design, collection, management, analysis and interpretation of data, writing of the report, and decision to submit the report for publication.

## Introduction

### Background and rationale {6a}

People with kidney failure require kidney replacement therapy for survival, which involves transplantation or dialysis, including hemodialysis (HD) or peritoneal dialysis (PD) [3]. Although patient survival is comparable between PD and HD in the first 3 years [4], PD is associated with a higher likelihood of returning to work, greater flexibility in dialysis schedules, cost savings from reduced travel to dialysis centers, better quality of life [5–7], patient satisfaction, ability to social distance, and independence [8]. PD is less expensive than HD in most countries and enables an increase in home-based dialysis therapies [4]. Despite these favorable features, the uptake of PD has diminished worldwide [9]. Estimates show that the percentages of prevalent patients with kidney failure who receive PD are below 10% in the USA, 22% in Canada, and 11% in Europe [10–13]. The prevalence of PD has decreased from 34% of the total dialysis population in Australia in 1995 to 18% in 2021 and from 61% of the New Zealand dialysis population in 1995 to 26% in 2021 [13].

Peritonitis causes PD cessation and death in 65% and 7% of patients on PD, respectively [13]. PD cessation causes patients to permanently transfer to HD or withdraw from treatment [14, 15]. PD-related peritonitis is also associated with peritoneal membrane dysfunction and higher morbidity, hospitalization, treatment costs, and mortality for up to 6 months after an episode [16]. The Standardized Outcomes in Nephrology – Peritoneal Dialysis (SONG-PD) initiative has identified PD-related infections as a core outcome for patients, caregivers, and clinicians for trials in patients on PD [14].

Substantial global variation in peritonitis rates exists among countries including Australia, France, New Zealand, Scotland, Taiwan, and the UK [17–20]. In addition, considerable variability exists in peritonitis rates between different PD units within the same country, with center-related factors outweighing patient-related factors [21]. PD is unique in that patients undertake their own treatment; thus, it is plausible that there could be a link between PD training and patient outcomes. Our publication following nationwide survey of PD units confirmed that differences in PD training practices currently exist in Australia [22]. It is hypothesized that a key center-related factor that may contribute to the variability in peritonitis risk is the variation in PD training [21, 23–25]. The ISPD has developed guidelines for PD training in clinical practice which are primarily based on adult education principles (andragogy) [26]. The ISPD guidelines provide recommendations concerning the performance of PD procedures, assisting patients to identify complications, and taking prompt, appropriate action [27, 28]. However, the effectiveness of standardized guideline-informed PD training curricula for trainers and patients has not been formally evaluated in a randomized controlled trial. A comprehensive PD training curriculum (TEACH-PD) for PD nurse trainers and patients has been developed by kidney nurses, doctors, educational experts and consumers, and, in alignment with the ISPD guidelines, utilizes evidence-based adult learning andragogy and eLearning pedagogy [29–31]. The curricula were identified as acceptable and usable by clinicians and patients in a feasibility study undertaken in two Australian units [30].

The Targeted Education ApproaCH to improve Peritoneal Dialysis outcomes (TEACH-PD) trial is a registry-based, pragmatic, multi-center, binational, cluster-randomized controlled trial (CRCT) to evaluate whether a guideline-informed standardized curricula of PD training for trainers and patients prevents PD-related infections and is cost-effective compared to standard care.

### Objectives {7}

The primary objective of the TEACH-PD trial is to determine whether the implementation of standardized training modules delays the time to the first episode of an exit site infection, tunnel infection, or PD-related peritonitis in incident PD patients compared to existing training practices.

The secondary objective is to determine whether a standardized PD training curriculum improves other important patient-centered outcomes and is cost-effective.

### Trial design {8}

The TEACH-PD trial is a pragmatic, multi-center, multinational, parallel arm, registry-based, CRCT, in which PD unit clusters are randomly assigned to implement TEACH-PD training curriculum or existing training practices for PD trainers and incident PD patients.

## Methods: Participants, interventions, and outcomes

### Study setting {9}

PD centers in Australia and New Zealand which provided PD training to more than ten patients over the 2 years prior to trial entry, as documented in the Australia and New Zealand Dialysis and Transplant (ANZDATA) Registry, are eligible. Clusters are randomly allocated to utilizing the TEACH-PD standardized curriculum or usual training practices for nurse trainers and incident PD patients. Allocation to treatment is stratified by site size according to the number of incident PD patients (small, medium, large, and very large). Investigators in Australia are asked to consider inclusivity regarding geography, unit size, location (including urban, regional, and rural), and center type (teaching and community hospitals). All 11 PD units in New Zealand have agreed to participate. The list of study sites is available elsewhere (see Additional file 1).

### Eligibility criteria {10}

To be eligible to participate in this trial, the participant must satisfy the following criteria:

Inclusion criteria:

1. Patients new to PD
2. Patients ≥ 18 years of age
3. Need to undergo PD training (patients who have a caregiver to be trained will also be included in the trial)
4. Are able to provide written informed consent

Exclusion criteria:

1. Patients who are established on PD (i.e., prevalent patients) or those patients or caregivers with a history of previous exposure to PD training as an adult will be excluded as their learning requirements are expected to be different from incident PD patients.
2. Paediatric patients are excluded as training modules were not designed or specifically tailored for their needs.

### Who will take informed consent? {26a}

TEACH-PD investigators will approach prospective trial participants to introduce the trial, describe the study, and answer questions. Prospective participants will be provided with the Patient Information Sheet and Consent Form. After discussing the trial, ample time will be given to the prospective participant to enquire about the trial and decide whether to participate. If the participant is unable to read the Patient Information Sheet and Consent Form, an impartial witness will be present during the entire discussion and will also be responsible for signing and dating the form on the participants’ behalf. In doing so, the witness attests that the information on the consent form was sufficiently and accurately explained to the participant, was understood by the participant, and that informed consent was freely given by the participant. If informed consent is provided, a consent form will be signed. The informed consent process covers the collection of study-related questionnaires and data linkage.

### Additional consent provisions for collection and use of participant data and biological specimens {26b}

The consent process includes a provision for data linkage to collect incidence of all cause hospitalizations with national and state-based health databases. No additional biological samples outside those collected as part of routine clinical care are being collected from participants.

## Intervention

### Explanation for the choice of comparators {6b}

Participating study sites in the experimental arm will implement PD training using TEACH-PD training curriculum/modules for the trainers and patients.

Participating study sites in the control group will continue pre-existing local PD training practice. A trial induction is completed at all control sites to assist with training in participant identification and screening, informed consent, data collection and entry, and outcome reporting.

### Intervention description {11a}

The TEACH-PD training curriculum and materials were developed by a core group of kidney nurses from The HOME Network (THN) [29, 30] in conjunction with senior medical clinicians from the Australasian Kidney Trials Network (AKTN), eLearning curriculum developers, consumer partners (i.e., patients and caregivers) and education experts, and were informed by the ISPD guidelines, utilizing evidence-based adult learning principles and best practice eLearning techniques [31]. A feasibility study was undertaken, involving ten PD trainers and 14 patients in two Australian PD units, to evaluate the feasibility and acceptability of the intervention, readability and usefulness of the education content and materials to nurse trainers and patients, and acceptability for participants [30]. The outcomes of this feasibility study informed the refinement of the TEACH-PD curriculum. As part of a process evaluation, 46 semi-structured interviews were conducted with PD nurse trainers and patients to ascertain their perspectives on the TEACH-PD intervention. These data were also used to refine the TEACH-PD curriculum intervention.

**Fig. 1:**
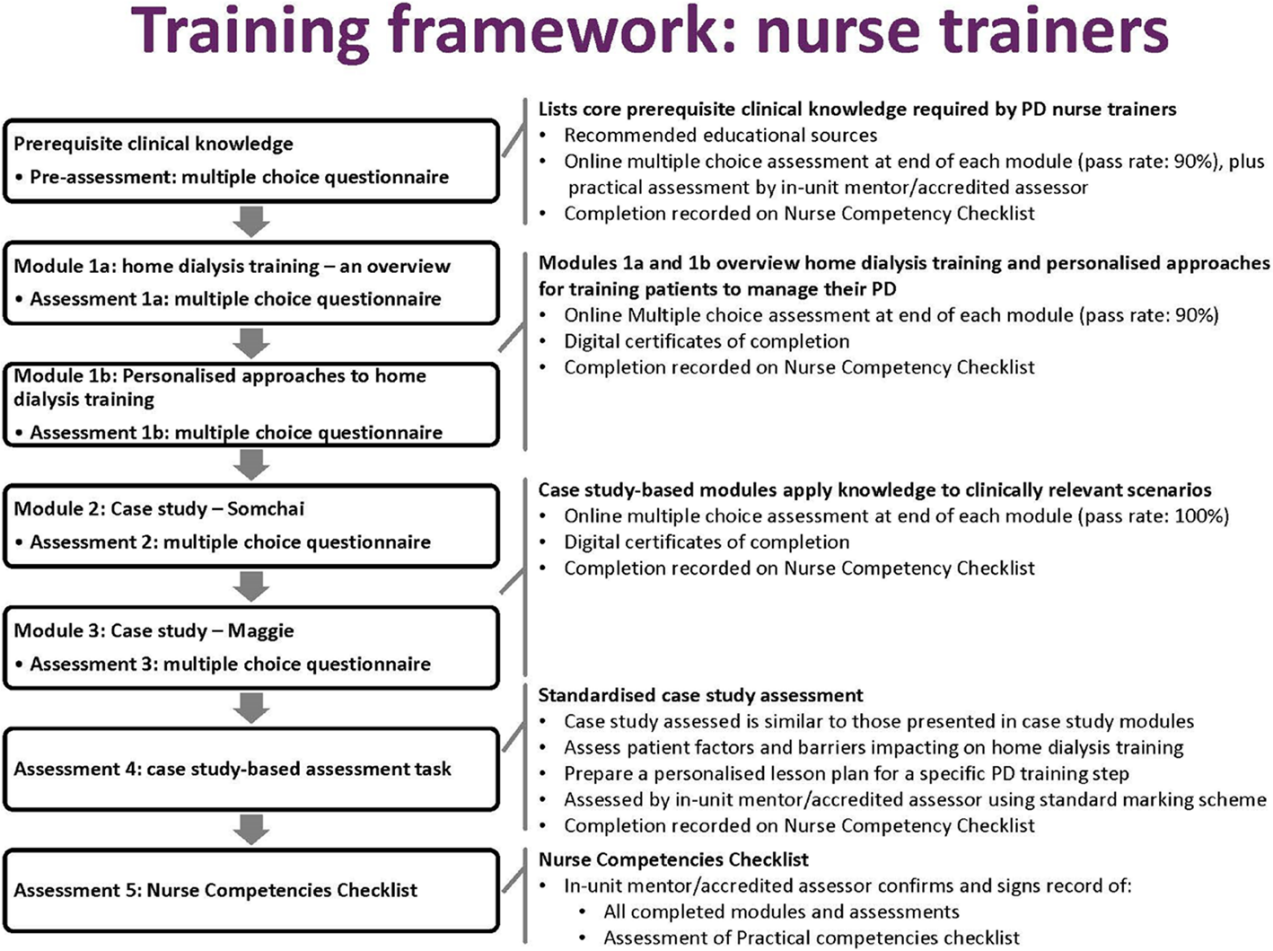
Training framework for PD nurse trainers

All PD nurse trainers at sites allocated to the TEACH-PD intervention will complete the following activities in the training framework [29] (Fig. 1: Training framework for PD nurse trainers):

#### Induction visit

Induction is conducted either in-person or virtually at each cluster site to introduce the TEACH-PD curriculum and its learning outcomes, the modules, and the importance of standardization. The induction provides an overview of the patient training manuals, learning prerequisites, and assessment methods aligned to the learning outcomes. The trial induction also assists site staff with training in participant screening and enrolment, informed consent, data collection and entry, and outcome reporting.

#### Prerequisite knowledge

All nurse trainers are required to demonstrate nursing-level understanding of all clinical content areas covered by the PD patient training modules. This includes a combination of theory-based and practical knowledge assessments relevant to the PD training environment and the nurse trainer’s role.

#### Web-based training modules

In this step, the PD nurse trainers work through the TEACH-PD online modules. Competencies in the core topics in each module and clinical case studies are assessed using multiple-choice questions in an online learning management system interface (Blackboard) (Fig. 1: Training framework for PD nurse trainers).

#### Case study modules

On completion of the case studies, PD nurse trainers are assessed by developing a care plan and a lesson plan for PD patient training.

#### Practical “Train the Trainer” modules

The final assessment is based on the completion of the Nurse Competencies Checklist, which is completed in partnership with an accredited TEACH-PD nurse assessor as a record of having achieved all learning outcomes and competencies of the TEACH-PD curriculum. The checklist includes a list of practical competencies that are demonstrated during hands-on training within the PD training unit. The accredited assessors are kidney nurse consultants, nurse practitioners, and nurse educators.

Study sites allocated to the intervention arm implement the TEACH-PD training curriculum/modules for nurse trainers and patients once 75% of eligible PD nurse trainers have completed and achieved competency in the TEACH-PD training curriculum. PD nurse trainers conduct patient training at the hospital, clinic, or in the participants’ homes according to local policies.

All participants' follow-up and PD management will be in adherence with the TEACH-PD curriculum principles. Annual refresher training is offered to all intervention sites. Financial support will be provided to all intervention sites by reimbursement for the time spent on nurse module training and assessment and for the annual refresher training. In addition, the research team will also be providing technical support and assistance.

### Criteria for discontinuing or modifying allocated interventions {11b}

The TEACH-PD intervention will only be discontinued at the request of the participant.

### Strategies to improve adherence to interventions {11c}

PD trainers’ adherence to the TEACH-PD training modules will be assessed using the techniques outlined in the training framework for PD nurse trainers. The PD patient adherence will be assessed by their PD trainer.

### Relevant concomitant care permitted or prohibited during the trial {11d}

All aspects of care provided will follow standard local practice for individuals with kidney failure being managed by a nephrologist.

### Provisions for post-trial care {30}

There are no provisions for post-trial care given the anticipated low risk of harm from a participant’s involvement in this trial.

### Outcomes {12}

#### Primary outcome

The primary outcome is time from the first day of PD training to the first occurrence of any PD-related infection (PD peritonitis, tunnel infection, or exit site infection), as defined by ISPD guidelines [28].

#### Secondary outcomes

The secondary outcomes are the individual components of the primary outcome: PD infection-related catheter removal, transfer to hemodialysis for greater than 30 days and 180 days, all-cause hospitalization, PD infection-related hospitalization, death from any cause, and quality of life.

A cost-effectiveness analysis from the perspective of the health funder will be completed.

### Participant timeline {13}

**Fig. 2:**
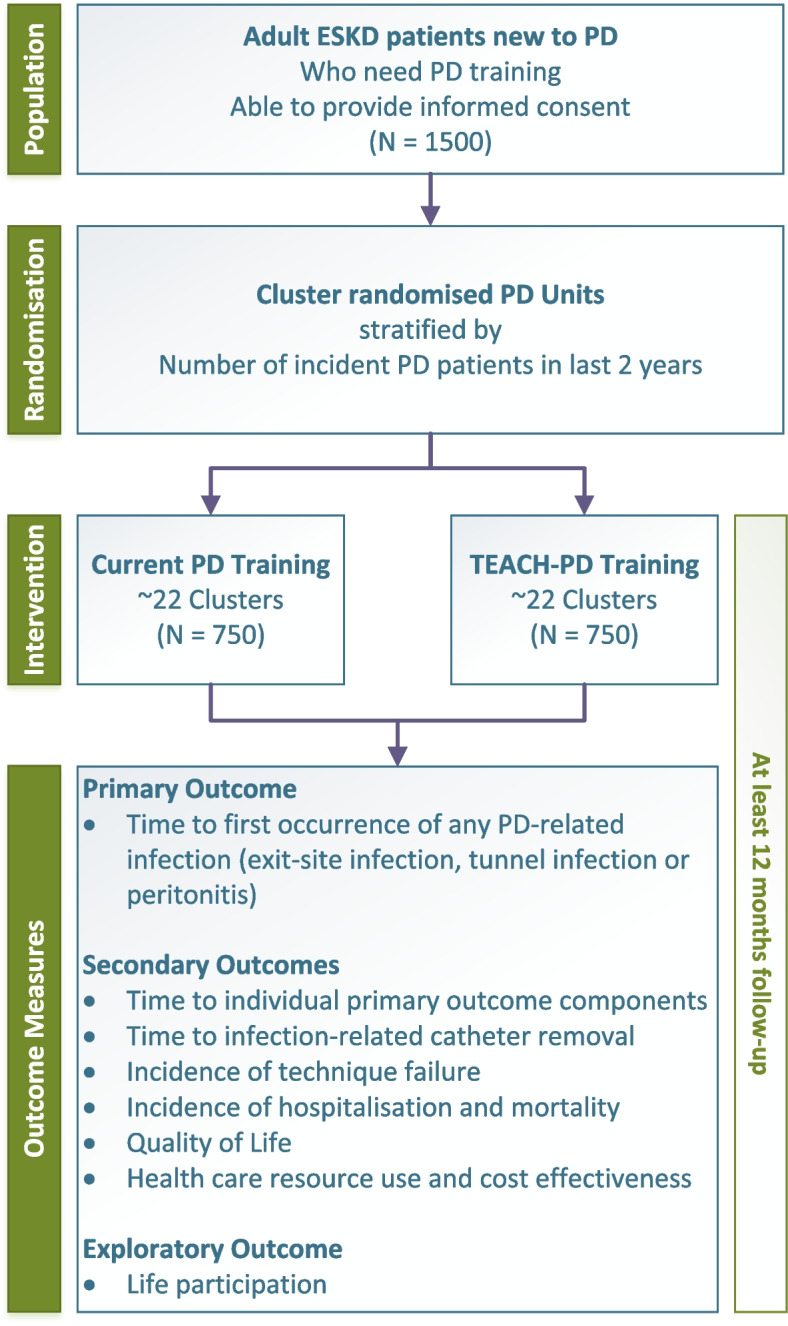
Trial schema showing an overview of the TEACH-PD trial

The follow-up period for participants is a minimum of 12 months with a targeted average follow-up period of 2 years (Fig. 2: Trial schema showing an overview of the TEACH-PD trial). Reasons for participant early exit of trial outcomes are kidney transplantation, permanent transfer to hemodialysis, death, transfer to a PD unit not participating in the trial, loss to follow-up, withdrawal of participant consent, or at the patient’s or treating physician’s request.

### Sample size {14}

A cluster randomized trial with an average cluster size of 37 incident participants over 2 years requires 42 clusters to achieve 80% power to detect a 20% reduction in the hazard of a first PD-related infection with the log-rank test in a cluster-randomized design at a type I error rate of 5%. This translates to approximately 650 events from approximately 1500 participants. The power calculation assumes 53% of the participants in the control group remain infection-free (data derived from HONEYPOT trial [32]) and an intra-cluster correlation coefficient of 0.001. Average cluster size is based on data from eligible Australian and New Zealand PD units in 2014–2015. Sample size calculations were performed using Power Analysis and Sample Size (PASS) Version 15.

### Recruitment and consent {15}

#### Unit recruitment

Eligible PD units (38 units in Australia, 11 units in NZ) will be invited to participate in the trial. Senior managers and clinical staff at each unit will be contacted by investigators and provided a TEACH-PD study synopsis. They will also be invited to attend one of the TEACH-PD virtual preparation sessions which provides additional information such as study background, rationale, and design. PD unit staff are offered opportunities to ask questions and seek further information.

#### Participant recruitment and consent

All patients new to PD requiring training in the participating units and are able to provide informed consent will be invited to take part in the trial. The timing of the initial approach to patients regarding participation will depend on unit practice patterns. This will potentially be made during pre-dialysis education, the first meeting with the PD team, at time of insertion of the PD catheter, at time of flushing of the PD catheter prior to training commencement, or on the first day of starting PD training. Every attempt will be made to approach the patients as early as possible.

All participant information sheets and consent forms (PISCFs) will be approved by an independent Ethics Committee with jurisdiction for the participating site. All participants will provide written informed consent prior to trial participation. If a participant is unable to read the PISCF, an impartial witness will be present during the entire discussion before the participant signs the consent.

## Assignment of interventions: Allocation

### Sequence generation {16a}

Random allocation of clusters will be 1:1 according to a computer-generated randomization algorithm using random permuted blocks within strata formed by country (Australia, New Zealand) and center size (small, medium, large, and very large) to minimize the risks of imbalance in baseline participant and center characteristics across different sized clusters.

### Concealment mechanism {16b}

Allocation will be concealed by holding the stratified random permuted blocks on a password-protected server at the Central Coordinating Center and available only to the trial unblinded statistician.

Due to the type of intervention, allocation is unmasked to the PD nurse trainers and the site contact at the local site.

### Implementation {16c}

All participants at a cluster randomised to an intervention group will receive the intervention, standardized PD training. Participants at a cluster randomised to a control group will receive usual care.

## Assignment of interventions: Blinding

### Who will be blinded {17a}

Emphasis has been placed on ensuring that blinding is maintained. Of the Steering Committee, only one member is unblinded to the details of the intervention and cluster allocation and one member is unblinded to allocation for New Zealand clusters only. Additionally, a minimum number of key operational staff are unblinded to cluster allocations, and all data that are presented are blinded. Unblinded members have received training about the maintenance of blinding in all aspects of the trial conduct.

### Procedure for unblinding if needed {17b}

The Data and Safety Monitoring Board and the independent statistician will make recommendations to the Trial Steering Committee, as required, should safety monitoring warrant unblinding.

## Data collection and management

### Plan for assessment and collection of outcomes {18a, 19}

Participant baseline and outcome data will be captured electronically within the ANZDATA Registry, the New Zealand Peritoneal Dialysis Registry (NZPDR), and a purpose-built REDCap (Research Electronic Data Capture) database according to the country-specific protocol appendices. REDCap [33] is a secure, web-based application designed to support data capture for research studies hosted by the University of Queensland. Original consent forms will be stored locally according to the International Council for Harmonization Good Clinical Practice (ICH GCP) and ethics committee approvals. Investigators will be required to maintain all study documentation, including consent documents, ethics committee approvals, and correspondence, for a period of 15 years after the closure of the trial. The complete participant data set will be made available by the Central Coordinating Center to researchers within the TEACH-PD CRCT study for analysis of sub-studies and country-specific outcomes after the dataset has been locked and analysis for the primary trial outcome is completed. The list of assessments for the participants during the TEACH-PD trial is outlined in Participant study assessments (Table 1).

**Table 1:** Participant study assessments

Timing of visit	Pre-training	Post-training (baseline)	Mth 6	Mth 12	Mth 18	Mth 24	End of Study
Screening	X						
Consent, demographics		X					
EQ-5D-5L		X	X	X	X	X	
Exit site infection^a^		X	X	X	X	X	X
Tunnel infection^a^		X	X	X	X	X	X
Peritonitis^a^		X	X	X	X	X	X

^a^Collected in real time as event occurs

### Main study measures {18a}

#### Demographic and clinical information

Participant demographic and clinical data are collected within the ANZDATA and the NZPDR Registries as part of routine Australian and New Zealand clinical practice. Baseline participant data will be extracted from the Registries including age (in years), sex, ethnicity, primary cause of kidney failure, height, weight, co-existing medical conditions (including cardiovascular disease, cerebrovascular disease, peripheral vascular disease, diabetes mellitus, and chronic lung disease), and smoking history. Additional information will be extracted during the enrolment period including kidney failure treatment, peritonitis (undifferentiated between local and systemic cause), tunnel infections and exit site infections, and infection treatment regimens.

#### Quality of life

**Fig. 3:**
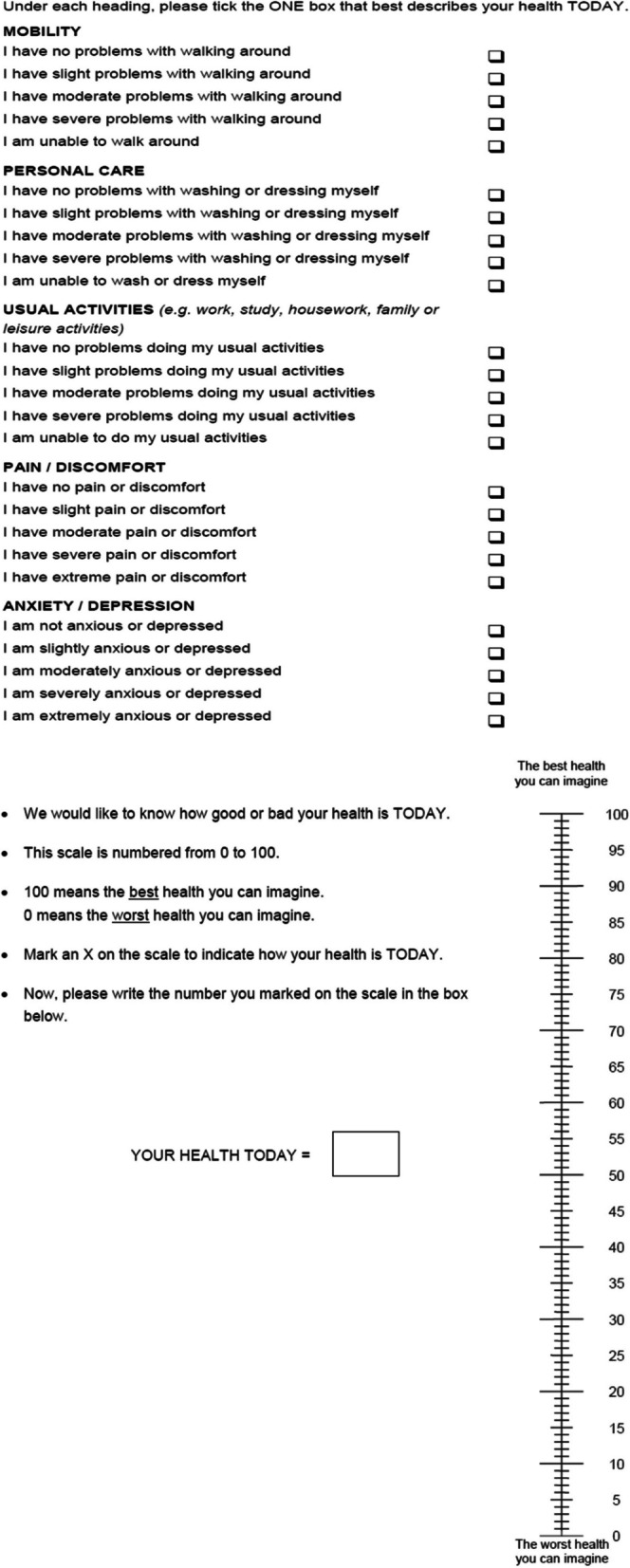
Example of EQ-5D-5L questionnaire (Australian version)

Quality of life is measured using the EuroQol (EQ-5D-5L, Fig. 3: Example of EQ-5D-5L questionnaire (Australian version)), a widely used instrument developed in Europe which assesses quality of life across five dimensions and five levels at baseline and at each 6-monthly visit. The utility value for estimation of quality adjusted life years (QALYs) will be estimated from the EQ-5D-5L scores using the Australian valuation set [34]. Study questionnaires are collected during participants’ regular clinic visits or e-mailed to participants and completed via REDCap or paper forms.

#### Healthcare utilization

Data on healthcare utilization during the trial will be obtained from national- and state-based health databases in Australia and the National Minimum Dataset in New Zealand. Data collected will include prescriptions, ambulatory healthcare encounters, pathology tests, imaging, and all-cause hospitalizations.

The cost of the intervention will be estimated based on patient training time captured using a purpose-built REDCap database in Australia and the NZPDR in New Zealand. Training times for the PD nurse trainers will also be collected and analyzed via the online time usage calculator in the TEACH-PD portal for each nurse trainer.

### Plans to promote participant retention and complete follow-up {18b}

Participant retention will be achieved by several strategies. Participant involvement throughout the trial development, activation and conduct, facilitated primarily by obtaining the Consumer Advisory Board’s input, ensures a patient-centred approach is applied to all trial activities and interactions with trial participants. The study staff at each site will be accessible to participants to answer their questions and respond to any concerns. Practical guidance and suggestions for participant retention awareness training will occur at the site initiation meetings and is documented in the Operations Manual.

For participants who withdraw from the trial, no further information will be collected from the date of withdrawal.

### Confidentiality {27}

Participants’ records and the data generated by the study will remain confidential in line with the recommendations of the National Health and Medical Research Council (NHMRC), the 2001 privacy legislation in Australia, and the Privacy Act 2020 in New Zealand. Any information that may identify a participant will be excluded from data presented in the public arena. Data will be stored in a secure, lockable location. Electronic data storage will have adequate password protection. The participants in this study will be identified only by initials and study number. De-identified information will only be released to the Central Coordinating Center or designee, according to ethics committees’ approval.

### Plans for collection, laboratory evaluation, and storage of biological specimens for genetic or molecular analysis in this trial/future use {33}

No additional biological samples outside those collected as part of routine clinical care are being collected from participants.

## Statistical methods

### Statistical methods for primary and secondary outcomes {20a}

Data will be analyzed at the patient level. Patient and treatment characteristics will be presented by study group using descriptive statistics. The primary outcome (coded as yes/no) and follow-up time (censored if < 24 months) will be displayed using Kaplan–Meier survival curves by group and by center size. Survival estimates of the treatment groups will be compared using the log-rank test for clustered data. Cox regression models with standard errors to allow for non-independent observations due to clustering will be used to assess the effect of the intervention on the primary and secondary outcomes. The models will be adjusted for cluster size category and country. The proportional-hazards assumption will be tested for each model. All data will be analyzed on an intention-to-treat basis with *p*-values less than 0.05 taken to indicate statistical significance.

### Interim analyses {21b}

No interim analyses are planned for outcomes.

### Methods for additional analyses (e.g., subgroup analyses) {20b}

Subgroup analysis and country specific outcomes analysis may be undertaken where possible.

### Methods in analysis to handle protocol non-adherence and any statistical methods to handle missing data {20c}

Protocol deviations will be investigated in sensitivity analyses. Imputation of randomly missing values will be tested during sensitivity analyses.

### Plans to give access to the full protocol, participant level-data and statistical code {31c}

Access will only be provided after the primary results of the trial and any pre-specified analyses are published. De-identified individual participant data will be made available upon request to a Data Access Committee, a review board set up to assess proposals based on sound science, benefit-risk balancing, and research team expertise. Appropriate data will be made available to approved proposals. This process will be in effect for a period of up to 5 years following publication of the main study results. After 5 years, the data will be available in the Sponsor’s data warehouse but without investigator support other than deposited metadata.

## Oversight and monitoring

The Australasian Kidney Trials Network (AKTN) is the coordinating centre on behalf of the University of Queensland. The AKTN will be responsible for convening and managing the Global Steering Committee. AKTN will also be responsible for developing and maintaining Global Steering Committee and Data and Safety Monitoring Board. The Central Coordinating Group (CCG) will be based at AKTN. AKTN will be responsible for reporting to the Global Steering Committee who in turn will be responsible for the oversight of the study. AKTN will also be responsible for acting as the Regional Coordinating Centre for Australia.

The Global Steering Committee (GSC) has ultimate responsibility for the study and will oversee the trial. The GSC will be responsible for study design; collection, management, analysis, and interpretation of data; writing of the report; and the decision to submit the report for publication. The Global Steering Committee will have ultimate authority over these activities. The project funders will not have any role in these activities. Alterations to the Charters may be made by the Global Steering Committee providing members of the Steering Committee have received 1 week’s notice of the proposed changes, and the changes are approved at a duly constituted meeting by a majority vote representing a minimum of one-third of the eligible voting members.

Each region will have a Trial Management Committee (TMCs) led by the Regional Chief Investigator, which will report to the Global Steering Committee and the Central Coordinating Centre. The Trial Management Committee will have responsibility for the delivery of the trial in their region and are answerable to the Global Steering Committee. Each region will have a Regional Coordinating Centre (RCC) consisting of the Regional Coordinator and Project Lead for that region. The Regional Coordinating Centre will be responsible for managing and supporting the activities of the Trial Management Committee and regional trial activities.

### Composition of the data monitoring committee, its role, and reporting structure {21a}

An independent four-member Data and Safety Monitoring Board (DSMB) with expertise in trial monitoring will be constituted by the TEACH-PD Global Steering Committee and operate in accordance with the Trial DSMB Charter. Members will have no financial or scientific conflicts of interest with the TEACH-PD CRCT trial. One DSMB member will be an experienced statistician with expertise in cluster randomized trials.

The DSMB will monitor accumulating safety and event data to examine data integrity and to protect the safety of trial participants. There are no formal statistical guidelines for early stopping of the trial. The DSMB will make appropriate recommendations to the GSC Co-Chairs regarding trial continuation and modifications to trial design and procedures. The GSC will retain sole decision-making responsibility for modifications to, or early stopping of, the trial.

### Adverse event reporting and harms {22}

No adverse events (serious or not) will be collected for this study. Trial related outcomes (exit-site infections, tunnel infections, and peritonitis) and deaths will be collected via the Renal Registries.

Incidence of all-cause hospitalisations will be collected via data linkage with national- and state-based health databases. All adverse events will be managed as per usual local clinical care practice.

### Frequency and plans for auditing trial conduct {23}

This study will be monitored by Regional Coordinating Centre or its designee in accordance with International Conference on Harmonisation Good Clinical Practices (ICH GCP), 21CFR Part 312. Monitoring efficiency will be optimized by a system of remote monitoring performed by AKTN. Risk-based monitoring is used for the study. If indicated, and with advance notice, study sites may be visited by a Clinical Monitor. The visits will be an opportunity to provide additional support and training to site staff, ensure the study is conducted according to the protocol, and in line with local regulatory requirements, including Good Clinical Practice. Source documents from which the data are obtained will be made available during the visit to the Clinical Monitor for review. Information garnered through monitoring will be fed back as appropriate to the independent Data and Safety Monitoring Board. The DSMB will make appropriate recommendations to the GSC Chair regarding trial continuation and modifications to trial design and procedures while maintaining confidentiality of the accumulating data. The GSC will retain sole decision-making responsibility for modifications to or early stopping of the trial.

### Plans for communicating important protocol amendments to relevant parties (e.g., trial participants, ethical committees) {25}

The Global Steering Committee will be responsible for ensuring any protocol amendments are approved by the responsible independent ethics committees and local site governance, and then communicated to the principal site investigators and site staff for implementation.

### Dissemination plans {31a}

Knowledge dissemination to consumers, clinicians, and policymakers will occur via the Renal Society of Australasia, Kidney Health Australia, Kidney Health New Zealand and New Zealand Patient Societies, the Australasian Kidney Trials Network (AKTN) website, Australian and New Zealand Society of Nephrology (ANZSN), International Society of Peritoneal Dialysis, peer-reviewed journal publications, state kidney networks, webinars, social media networks (e.g., NephJC), clinical practice guidelines, and presentations at national and international scientific meetings.

## Discussion

PD-related infections, especially peritonitis is a significant cause of mortality, morbidity, and hemodialysis transfer for patients undergoing PD [21, 23]. PD-related peritonitis rates vary widely across different centers and several studies have reported that this variation is mainly attributed to center-related rather than patient-related factors [22, 26]. In particular, variation may be influenced by differing PD training practices and approaches. Although the ISPD has documented and established guidelines for PD training, significant evidence gaps exist in PD training approaches. Several reviews have highlighted the association between patient training and PD-related outcomes [4, 12, 24]. However, there is no robust evidence evaluating the effectiveness of standardized PD training practices and curricula with regard to PD-related infection or other patient-important outcomes [24].

The TEACH-PD trial aims to provide robust evidence that addresses this evidence gap in PD training. The cluster randomized design of the trial reduces complex care intervention contamination between the intervention and control arms. This study is designed to recruit all patients new to PD and requiring PD training in the participating units. Patients who are established on PD (i.e., prevalent patients) or those with a history of previous exposure to PD training are excluded. The eligibility criteria are deliberately broad to reflect routine clinical practice (i.e., non-English-speaking patients are not excluded). Pediatric patients are excluded as the training modules have not been designed or specifically tailored for their needs. In addition, those patients with a history of receiving PD training are excluded as their learning requirements are expected to be different from those of incident PD patients.

A key outcome of the TEACH-PD CRCT includes the evaluation of the safety, efficacy, cost-effectiveness, and patient-reported experience of a standardized PD training approach and curriculum, compared with current training. With PD-related infections identified as a critical outcome in PD trials, the findings of this study will be disseminated to kidney healthcare professionals and consumers via Kidney Health Australia, Kidney Health New Zealand, publications in peer-reviewed journals, state kidney networks, clinical practice guidelines, and presentations at national and international scientific meetings.

A key strength of the TEACH-PD trial is the utilization of a vigorous and adequately powered methodological approach to evaluate a standardized PD training curriculum for both trainers and patients. Both the approach and curriculum have been developed by patients, PD nurses (through THN) [29], doctors and education experts, and have been demonstrated as feasible and acceptable to clinicians and patients [30]. The TEACH-PD trial has a practical focus with broad eligibility criteria, avoidance of extra blood tests, use of local patient care practices, and minimal additional data collection, as data are collected via linkage with national and state-based administrative data and the ANZDATA and NZPD Registries. The registry-based design of the trial offers the advantage of rapid enrolment of eligible participants and complete (and potentially extended) follow-up of all study participants. The investigators acknowledge that PD nurses are the integral aspect of TEACH-PD and the key to its success, such that every effort has been made in designing this study to avoid excessive data collection and management burden. By following a pragmatic design with broad inclusion criteria, patient co-production, and involvement of clinical staff in the research design, the ability to implement the trial’s findings have been maximized [25].

Nonetheless, the TEACH-PD trial also has its limitations. First, it is an open-label trial and therefore potentially introduces detection and performance biases. Second, the trial is limited to PD centers in Australia and New Zealand, such that the findings may not be generalizable to other countries and income settings. TEACH-PD investigators acknowledge and have considered expanding the trial to international sites to improve external validity. Third, there exists a lag time between activation of control and intervention sites due to the time taken to complete the TEACH-PD training curriculum at intervention sites. However, the lengthy follow-up time for both arms will minimize any performance biases. Finally, training practices among sites in the control arm are heterogeneous and may increase outcome variation in the control arm, limiting comparability between arms. In addition, if a TEACH-PD patient from an intervention site is hospitalized and is assisted in their dialysis, this may be performed by a nurse who has not been trained in the TEACH-PD intervention.

In summary, PD training is widely acknowledged as being critically important for mitigating infection risk and minimizing HD transfer. There is no high certainty evidence guiding how, when, where, or by whom training is best performed, and consequently, PD training practices are highly variable within and between countries. The TEACH-PD CRCT will provide high certainty evidence regarding whether guideline-informed PD training curricula mitigate PD-related infections.

## Trial status

All PD units in New Zealand were eligible for the TEACH-PD study. Forty-two clusters have been randomized and it is anticipated that recruitment of 1500 incident PD patients in Australia and New Zealand will be completed by June 2023.

### Supplementary Information

**Additional file 1. **List of study sites.

**Additional file 2. **Funding documents.

**Additional file 3. **Ethics Approval (Australia).

**Additional file 4. **Ethics Approval (New Zealand).

## Data Availability

The datasets used and/or analyzed during the current study are available from the corresponding author on reasonable request.

## Abbreviations

AKTN: Australasian Kidney Trials Network
ANZDATA: Australia and New Zealand Dialysis and Transplant Registry
CRCT: Cluster-randomized controlled trial
DSMB: Data and Safety Monitoring Board
GCC: Global Coordinating Committee
GCP: Good clinical practice
GSC: Global Steering Committee
HD: Hemodialysis
ICH: International Committee for Harmonization
IEC: Independent Ethics Committee
ISPD: International Society for Peritoneal Dialysis
NHMRC: National Health and Medical Research Council of Australia
NZ: New Zealand
NZPD: New Zealand Peritoneal Dialysis Registry
PD: Peritoneal dialysis
PISCF: Patient Information Sheet and Consent Form
REDCap: Research Electronic Data Capture
SPIRIT: Standard Protocol Items: Recommendations for Interventional Trials
THN: The HOME Network
