# Supplementary material for: Multi-center, pragmatic, cluster-randomized, controlled trial of standardized peritoneal dialysis (PD) training versus usual care on PD-related infections (the TEACH-PD trial): trial protocol
Source: Trials. 2023 Nov 14;24:730. doi: 10.1186/s13063-023-07715-0 (PMC10647147; doi:10.1186/s13063-023-07715-0)
Supplement: Supplementary file 1 — Additional file 1. List of study sites. [file 13063_2023_7715_MOESM1_ESM.pdf]

TEACH-PD Trial: List of study sites

Auckland Hospital
Austin Hospital
Barwon Health
Blacktown Mt Druitt Hospital - Nepean Hospital
Cairns Hospital
Canberra Hospital
Christchurch Hospital
Dunedin Hospital
Fiona Stanley Hospital
Gold Coast University Hospital
Gosford Hospital
Hawke's Bay Hospital
Launceston Hospital
Lismore Base Hospital
Liverpool Hospital
Logan Hospital
Mackay Base Hospital
Middlemore Hospital
Monash Medical
North Shore Hospital & Waitakere Hospital
Orange Health Service
Palmerston North Hospital (includes Wanganui Hospital)
Prince of Wales Hospital
Princess Alexandra Hospital
Royal Adelaide Hospital
Royal Brisbane & Women's Hospital
Royal Darwin Hospital
Royal Hobart Hospital
Royal Melbourne Hospital
Royal North Shore Hospital
Royal Perth Hospital
Royal Prince Alfred Hospital – Concord Hospital
Sir Charles Gairdner Hospital
St George Hospital
St Vincent’s Hospital Melbourne
St Vincent's Health Sydney
Sunshine Coast University Hospital - Nambour Hospital
Tamworth Hospital
Taranaki Hospital
Toowoomba Hospital
The Tweed Hospital
Waikato Hospital (includes Gisborne Hospital)

Wellington Hospital (Nelson Hospital and Blenheim Hospital)
Whangarei Hospital
