# Supplementary material for: Multi-center, pragmatic, cluster-randomized, controlled trial of standardized peritoneal dialysis (PD) training versus usual care on PD-related infections (the TEACH-PD trial): trial protocol
Source: Trials. 2023 Nov 14;24:730. doi: 10.1186/s13063-023-07715-0 (PMC10647147; doi:10.1186/s13063-023-07715-0)
Supplement: Supplementary file 2 — Additional file 2. Funding documents. [file 13063_2023_7715_MOESM2_ESM.zip › Funding_HRC_19R1_20.pdf]

31 May 2019

Health Research Council of New Zealand

Professor Dr Suetonia Palmer
University of Otago
suetonia.palmer@otago.ac.nz

Dear Suetonia and Team

Project Application for Health Research Council Funding
HRC Reference: 19/290
Teaching to improve health outcomes for peritoneal dialysis: The TEACH-PD trial

The Health Research Council of New Zealand (HRC) has completed the assessment of all Project proposals for the 2019 funding round. I am very pleased to advise that your proposal has been successful. The Council has offered funding to the level set out on the attached draft contract Third Schedule.

Please note that budgetary changes to your original application may necessitate a change in your research objectives. These changes, or other enquiries relating to the administrative aspects of your funding, should be directed to your Research Office. Once all changes have been agreed, contract documents will be sent to your Research Office for signing. A contract will not be formed until the HRC receives a completed "Staff Declaration - HRC Contracts Form" (available from your Research Office), amended objectives and milestones (Fourth Schedule) and any special conditions or requirements set out in the draft Third Schedule have been met. The *HRC Rules*, which form part of the contract, is available on the HRC website.

Some key conditions of the contract include best efforts to complete the proposed research, fulfilment of reporting requirements noting problems or delays as soon as they occur, changes or significant absences of key staff and significant changes to research objectives/ milestones. In your acceptance of this offer please indicate for our records whether an ethical approval is required for the planned research. Regular reporting aims to identify any issues or concerns as well as highlight positive outcomes of the research. Please let us know directly of any newsworthy impacts of our funding. Contract variations, such as time extensions, must be submitted to the HRC by your Research Office. All research reports can be now submitted on the HRC Gateway.

Your Research Office has been requested to return the draft Third and Fourth Schedules and signed Staff Declaration form by 14 June 2019. The Staff Declaration form should also indicate time only staff and their FTE on the contract. Your Research Office is required to return the signed contract to the HRC by 2 August 2019. Unless your Research Office has received written authority from the HRC, your contract must commence no later than 1 September 2019. The funding may be withdrawn and returned to the HRC funding pool if this condition is not met.

Please note that the HRC will be making a media announcement about the outcome of this round in mid-June 2019. Media activities initiated by your institution may follow the HRC's announcement but must not be before this announcement. This includes posting any result details on your websites. Please contact the HRC if you would like us to provide comment for your institution's media funding announcements as we would be happy to do so.

Some of the points raised during the assessment of your application are outlined in the enclosed Review Summary. If you wish to discuss the result of your application please address your enquiry, in the first instance, to your host institution and request that they write to Dr Deming Gong, Manager Research Investment -Contracts, at the HRC.

Note that all investigators receiving contract funding from the HRC must make themselves available, as reviewers or assessing committee members whenever possible. Please update your HRC Gateway profile to nominate yourself for HRC assessing committee membership.

I would like to add a personal note of congratulations on your success and I look forward to hearing of the progress and outcomes of your research.

Ngā mihi

Kath (McPherson)

Well done! You are going to be busy for sure! K.

Professor Kathryn McPherson
Chief Executive

encls Review Summary
Draft Third Schedule
Draft Fourth Schedule

cc University of Otago Research Office

SAC Review Summary: Projects

**HRC Reference #**	19/290	**Applicant Surname**	Palmer
**Title of Research**	Teaching to improve health outcomes for peritoneal dialysis: The TEACH-PD trial		
**Host**	University of Otago, Christchurch		

With regard to the criteria for assessing and scoring research proposals:

1. **What issues were considered by the Science Assessing Committee as important enough to influence the scoring of this proposal? (200-300 words)**

- Well-designed important large pragmatic study with well-supported rationale and acknowledged major issue in the population generally and especially among Māori.
- Appropriate cluster intervention and primary composite endpoint, with important secondary endpoints.
- Data collection embedded within existing registries in New Zealand and Australia.
- Results will inform and be incorporated into clinical guidelines.
- Named primary investigator has outstanding track record relative to opportunity and has assembled an appropriate and well-credentialled multidisciplinary team.
- There has been significant consumer involvement including through feedback and subsequent modifications of the curriculum. Results of this study will be important for Māori and Māori consultation has occurred; with active Māori recruitment encouraged, planned involvement of Māori clinical staff and planned building of Māori health research capacity.
- The sample size and statistical analysis were clear, including the health economic analysis.

2. **Other Comments**

- Note was made that the pilot study was conducted in Newcastle, Australia, raising queries about relevance to New Zealand including Māori.

THIRD SCHEDULE SUMMARY - RESEARCH ACTIVITY DETAILS AND FUNDING

Research Provider: University of Otago

Contract Type: Project

Contract Number: 19/290

First Named Investigator Professor Dr Suetonia Palmer

Named Investigators: Professor Robert Walker, Dr Thu Nguyen, Associate Professor Rachael Walker, Dr Walaa Saweirs, Dr Yeoungjee Cho, Professor Josephine Chow, Professor David Johnson, Professor Neil Boudville, Professor Kirsten Howard, Ms Elaine Pascoe

DRAFT

Title: Teaching to improve health outcomes for peritoneal dialysis: The TEACH-PD trial

**Start Date:**	1/07/2019	**Completion Date:**	30/06/2024	**Term:**	60

Organisations Sharing in Funding:

Reporting Dates: Annually on the anniversary of the grant plus 1 month

Budget Note: Incl. $253,528 funds for subcontract with Australasian Kidney Clinical Trials to be released at contract commencement. Incl. $49,722 funds for subcontract with Eastern Institute of technology to be released at contract commencement. Incl. $41,328 funds for subcontract with ADHB to be released at contract commencement. Incl. $48,233 funds for subcontract with Northland DHB to be released at contract commencement. Five months payment retained subject to satisfactory end of contract report.

Budget Outline (GST Exclusive)

	$
**Total Salary:**	383,815.00
**Total W. Expenses:**	652,506.00
**Total Overheads:**	403,005.75
**Total Budget:**	1,439,326.75
**Administered by HRC:**	0.00
**Host Budget:**	1,439,326.75

Monthly Payment:

23,988.78

Payment Process:

monthly on the 20th day of the month

Investigators/Staff/Key Personnel

Professor Dr Suetonia Palmer	0.10
Professor Neil Boudville	0.03*
Dr Yeoungjee Cho	0.03*
Professor Josephine Chow	0.03*
Professor Kirsten Howard	0.05*
Professor David Johnson	0.03*
Dr Thu Nguyen	0.03
Ms Elaine Pascoe	0.05*
Dr Walaa Saweirs	0.03
Associate Professor Rachael Walker	0.05
Professor Robert Walker	0.03
Data Manager	0.05
Lead Researcher	0.20
Manager 1	0.03
Nurse	0.10
Research Associate	0.20
Research Nurse	0.80
Statistician	0.03
**Total FTE:**	**1.87**

Personnel marked * have a time commitment only

DRAFT

FOURTH SCHEDULE -
RESEARCH OBJECTIVES AND MILESTONES

Note that this page will form the basis of the contract for progress reports

#	Objectives
1	To conduct a registry-based, pragmatic, cluster-randomised controlled trial (RCT) to test the hypothesis that a standardised training curriculum for peritoneal dialysis (PD) nurse trainers and patients reduces PD-related infections compared to standard care.
2	To evaluate whether a standardised PD training curriculum improves utility-based quality of life compared to standard care.
3	To conduct an economic evaluation on the health-care resource use and cost-effectiveness of a standardised PD training curriculum compared to standard care.

Year # for Completion of Milestones for each Objective		
Year #	Milestones	Objective(s)
1	Set-up of NZ coordinating site including personnel (coordinator and data manager)	1,2,3
1	Completion of NZ ethics approvals	1,2,3
1	Commence trial governance processes at all sites	1,2,3
1	National investigator start-up meeting	1,2,3
1	NZ PD registry linked to ANZDATA registry	1,2
1	A stepped rollout of site initiations and staff training	1,2
1	Site initiation and recruitment commencement at 4 sites	1,2,3
1	Recruitment of first 50 patients	1,2
2	Site initiations and staff training at all sites completed	1,2
2	Interim recruitment target = 200	1,2,3
2	Follow-up, data entry and monitoring of enrolled patients	1,2,3
3	Recruitment completed for all sites	1,2
3	Follow-up, data entry and monitoring of all patients	1,2,3
4	Complete follow-up, data entry and monitoring of all patients	1,2,3
5	Site closeouts, data cleaning and database lock	1,2,3
5	Prepare and submit Statistical Analysis Plan for publication	1,2,3
5	Data analysis for primary outcome	1,2,3
5	Commence drafting of primary publication	1,2,3
5	Data linkage to the Ministry of Health Health Tracker program for economic analysis	3
5	Commence economic analyses	3

Contract Number:19/290 Date Printed: 21-May-2019

DRAFT
