# Supplementary material for: Multi-center, pragmatic, cluster-randomized, controlled trial of standardized peritoneal dialysis (PD) training versus usual care on PD-related infections (the TEACH-PD trial): trial protocol
Source: Trials. 2023 Nov 14;24:730. doi: 10.1186/s13063-023-07715-0 (PMC10647147; doi:10.1186/s13063-023-07715-0)
Supplement: Supplementary file 2 — Additional file 2. Funding documents. [file 13063_2023_7715_MOESM2_ESM.zip › Funding_MRFF_APP1170238R1.pdf]

The Hon. Greg Hunt MP
Minister for Health

MEDIA RELEASE

15 February 2019

$38.6 million for rare cancer and diseases clinical trials

The Liberal National Government will provide $38.6 million to support 23 new clinical trials to improve treatments and discover cures for debilitating and deadly rare cancers and rare diseases.

The Rare Cancers, Rare Diseases and Unmet Need Clinical Trials program is a competitive program that focuses on clinical trials that address areas of health burden and unmet need including brain cancer.

The program includes a trial to evaluate the effectiveness of different chemotherapy treatments for the highly fatal glioblastoma brain cancer, a trial to use precision medicine in treating relapsed high-grade Glioma, a trial to test the effectiveness of chemotherapy treatments for children and adolescents with Medulloblastoma brain tumours and a trial to use the a new combined chemotherapy treatment to target brain tumours in children.

This funding, through our Government's landmark Medical Research Future Fund (MRFF), aims to enhance clinical trial activity, treatments, cures, and extend and improve quality of life for Australians living with devastating conditions.

The funding announced today includes:

- $10.8 million for rare cancer clinical trials
- $18.3 million for rare diseases clinical trials
- $6.6 million for unmet needs clinical trials
- $4.5 million for brain cancer clinical trials. The Government will invest $2.9 million with an additional $1.6 being contributed by Carrie's Beanies 4 Brain Cancer and the Mark Hughes Foundation through our Government's Australian Brain Cancer Mission.

We have seen increased survival rates for cancers such as breast and bowel cancer. However, survival rates for rare cancers such as brain cancer and lymphoma have remained relatively unchanged for some time around the world.

Clinical trial programs like this will bring hope and save lives.

Authorised by Greg Hunt MP, Liberal Party of Australia, Somerville, Victoria.

The total $4.5 million funding for four brain cancer clinical trials comprises of Government and private funding will deliver four clinical trials that targets brain cancer in adults and children.

Our Government is partnering with Carrie's Beanies 4 Brain Cancer and the Mark Hughes Foundation to deliver four trials.

This is the third round of grants announced the under Rare Cancers, Rare Diseases and Unmet Need Clinical Trials program taking the total funding announced under this program to $75 million.

We hope and believe that these brain cancer trials will take Australia closer to leading the world in doubling survival rates for those with brain cancer over the next decade.

These brain cancer trials are on top of the $10 million already invested under the Australian Brain Cancer Mission to support the Zero Childhood Cancer Initiative and key brain cancer clinical trials groups for adults and children.

This latest funding builds on more than $36 million already announced under the Rare Cancers, Rare Diseases and Unmet Needs Clinical Trial program, which was launched in January 2018.

The $20 billion Medical Research Future Fund was established as an endowment fund to provide a sustainable source of funding for vital medical research.

It is the single largest boost in health and medical research funding in Australia's history.

Authorised by Greg Hunt MP, Liberal Party of Australia, Somerville, Victoria.

Rare Cancers

Chief Investigator	Institution	Title	Funding
Doctor Martin Campbell	Monash University	FaR-RMS: Frontline And Relapse study in RhabdoMyoSarcoma	$1,353,514.40
Professor Maher Gandhi	The University of Queensland	An Open Label, Multicentre, Phase One Study Incorporating Early Application of CAR T cells for Primary Refractory Aggressive Lymphoma	$3,596,966.90
Associate Professor Judith Trotman	University of Sydney	PETReA: Phase 3 evaluation of PET-guided, Response-Adapted therapy in patients with previously untreated, high tumour burden follicular lymphoma.	$787,068.85
Doctor Constantine Tam	University of Melbourne	The AIM2 Study: Genomically Guided Novel Combination Treatment of Mantle Cell Lymphoma	$2,005,391.40
Doctor Wen Xu	The University of Queensland	A Randomised Phase II Trial of Adjuvant Avelumab in Patients with Early Stage Merkel Cell Carcinoma	$1,632,095.75
Associate Professor Andrew Wei	Monash University	Novel Veneticlax Combinations to Improve Outcomes in Unfit Older Patients with Acute Myeloid Leukaemia	$1,380,297.60

Rare Diseases

Chief Investigator	Institution	Title	Funding
Professor Anne Chang	Menzies School of Health Research	Improving outcomes of children and young adults with primary ciliary dyskinesia (PCD): a multi-centre, double-blind, double-dummy, 2x2 factorial, randomised controlled trial (RCT)	$2,375,118.40
Associate Professor Craig French	Monash University	ErythroPOietin alfa to prevent mortality and reduce severe disability in critically ill TRAUMA patients: a multicentre, stratified, double blind, placebo randomised controlled trial. (The EPO-TRAUMA trial)	$3,509,303.10
Associate Professor Meg Jardine	University of New South Wales	BEAT-Calci (Better Evidence And Translation in Calciphylaxis)	$2,201,943.70
Professor David Johnson	The University of Queensland	The TEACH-PD study: a Targeted Education ApproaCH to improve Peritoneal Dialysis outcomes	$2,383,206.90
Professor Matthew Kiernan	University of Sydney	ALS Trials Australia (ALSTA) - to develop precision medicine	$1,704,432.20
Professor Kei Lui	University of New South Wales	Does WithHolding Enteral feeds Around blood Transfusion reduce the incidence of necrotising enterocolitis (NEC) in very preterm infants? The international WHEAT Study	$1,606,825.80
Associate Professor James McAuley	University of New South Wales	MEMOIR: A multi-site placebo-controlled trial of memantine and graded motor imagery for complex regional pain syndrome	$922,314.05
Professor Terence O'Brien	Monash University	Evaluating the effectiveness and safety of sodium selenate as a disease modifying treatment for patients with behavioural variant Frontotemporal Dementia (bvFTD)	$1,604,184.40
Professor Nora Shields	La Trobe University	Improving muscle strength in young people with Prader-Willi syndrome	$874,179.45
Associate Professor Germaine Wong	University of Sydney	NAVMAN TRIAL: A multi-centre, dynamic, waitlist randomised controlled trial of patient navigators in children with chronic kidney disease	$1,093,680.90

Unmet Need

Chief Investigator	Institution	Title	Funding
Professor Michael Berk	Deakin University	The Candesartan Adjunctive bipolar DEpression Trial - CADET	$2,428,397.10
Associate Professor Martin Gallagher	University of New South Wales	Aldosterone bloCkade for Health Improvement EValuation in End-stage renal disease (ACHIEVE) study	$2,850,898.40
Doctor Jackson Thomas	University of Canberra	Evaluation of a bush medicine-based treatment for scabies in Australian Aboriginal children	$1,294,542.05

Brain Cancer

Chief Investigator	Institution	Title	Funding
Doctor Craig Gedye	University of Sydney	MAGMA: Multi-Arm GlioblastoMa Australasia Trial	$2,421,993.95
Associate Professor Nicholas Gottardo	Monash University	SJ-ELiOT: St Jude - Phase 1 Evaluation of LY2606368, Molecularly-Targeted CHK1/2i Therapy, in Combination with Cyclophosphamide or Gemcitabine for Children and Adolescents with Refractory or Recurrent Medulloblastoma Brain Tumours	$452,567.20
Doctor Jordan Hansford	Monash University	COZMOS: Phase I/Ib trial of COmbined 5'-aZacitidine and carboplatin for recurrent/refractory paediatric brain and solid tuMOurs	$250,709.00
Hao-Wen Sim	University of Sydney	PICCOG: PARP and Immune Checkpoint inhibitor COmbination for relapsed IDH-mutant high-grade Glioma	$1,391,472.20

Authorised by Greg Hunt MP, Liberal Party of Australia, Somerville, Victoria.
