# Supplementary material for: Multi-center, pragmatic, cluster-randomized, controlled trial of standardized peritoneal dialysis (PD) training versus usual care on PD-related infections (the TEACH-PD trial): trial protocol
Source: Trials. 2023 Nov 14;24:730. doi: 10.1186/s13063-023-07715-0 (PMC10647147; doi:10.1186/s13063-023-07715-0)
Supplement: Supplementary file 2 — Additional file 2. Funding documents. [file 13063_2023_7715_MOESM2_ESM.zip › Funding_MSH Research Support SchemeR1.pdf]

Centres for Health Research

Enquiries To: Research Support Coordinator
Telephone: 07 3443 8057
Email: CHR-RSS@health.qld.gov.au
Date: 28 November 2018

Prof David W Johnson
Director
Department of Nephrology
Princess Alexandra Hospital

Dear David,

It gives us great pleasure to confirm that your 2019 Metro South Health Research Support Scheme Project Grant application for the study entitled "***The TEACH-PD study: a Targeted Education ApproaCH to improve Peritoneal Dialysis outcomesargeted Education ApproaCH to improve Peritoneal Dialysis outcomes***" has been successful.

- The total amount of the award is $99,995.32 to be expended within two years, commencing January 2019. The grant is funded by the Metro South Study, Education and Research Trust Account (SERTA).
- To receive this funding, you must accept the Conditions of Award provided on the following pages, and email the signed document to the Centres for Health Research at CHR-RSS@health.qld.gov.au **by midday Friday 14th December 2018**.
- You must notify the Centres for Health Research in writing of any additional funding obtained for this project soon as feasible.
- Subject to confirmation of acceptance by the due date, you will be able to access grant funds from January 2019 by submitting a Grant Payment Request form (*attached*) to your Business Manager or Finance Director along with a copy of this letter.
- A Final Report must be submitted to the MSH Research Support Scheme by 30 April 2021.

On behalf of the Metro South Health Research Committee and SERTA, I would like to congratulate you and your team on your success and wish you all the best for your research.

Yours sincerely,

Prof Tim Geraghty, MBBS FAFRM (RACP)
Acting Chair, Research Metro South Health

CONDITIONS OF AWARD: 2019 METRO SOUTH HEALTH RESEARCH SUPPORT SCHEME GRANT

- All research must comply with the ethical standards as set out by the National Health and Medical Research Council (NHMRC) *National Statement on Ethical Conduct in Human Research (2007) -updated 2018* and the Human Research Ethics Committee(s) (HREC) / Animal Ethics Committee(s) providing ethical clearances.
    - The grant recipient may be required to provide evidence of HREC approval, on request from the Centres for Health Research (CHR);
    - The grant recipient is required to submit an Annual Progress Report to the approving HREC as a condition of this grant and in accordance with the NHMRC Statement.
- All clinical research conducted at a Metro South Health (MSH) site must be authorised by the Metro South Research Governance Office.
    - The grant recipient may be required to provide evidence of Site Specific Assessment (SSA) authorisation, on request from the CHR;
- SERTA reserves the right to terminate funding in circumstances including, but not limited to the following:
    - inability to demonstrate appropriate HREC approval or SSA authorisation;
    - unsatisfactory progress as determined by the MSH Research Committee.
- Funding may only be applied to the work described within the Research Proposal submitted as part of the application. The grant funds may not be used to pay for university overhead costs.
- The majority of the research must be conducted at a MSH site unless otherwise agreed in writing.
- Award payments must be initiated by the Principal Investigator (applicant) via the submission of a completed Grant Payment Request (GPR) form, including signatures to SERTA.
- Upon receipt of a completed and signed GPR form the grant will be progressed for payment, in one instalment, according to the amount requested in the Budget Proposal submitted as part of the application.
- Funds must be expended by December 31st of the final year of funding unless a request for an extension has been made in writing to and approved by the Chair, Research Metro South Health.
- Unspent funds must be returned to SERTA.
- In the event of a period of absence for more than 30 working days (e.g. maternity leave, sabbatical, long service leave, interstate or overseas travel), the Principal Investigator (recipient) must apply in writing to the Chair, Research Metro South Health, for a pause in or extension to grant duration.
- The Principal Investigator must notify the CHR in writing of:
    - any additional funding received for this project;
    - any change in appointment or employment of the grant recipient;
    - or any change in location of the grant recipient or research project activity from a MSH site or MSH campus based academic partner university, school or research institute.
- Subject to prior notification of a change in employment circumstances or location of the Principal Investigator, the MSH Research Committee may consider requests for research to transfer to an appropriate alternate Principal Investigator and make a recommendation for transfer of the grant. SERTA may approve or reject a recommendation for transfer of grant at its discretion.
- Appropriate recognition of SERTA, and the MSH Research Support Scheme must be included in all publications and presentations resulting from the work supported by this grant.
- Recipients should be available upon request to present the outcomes of their MSH Research Support Scheme funded research at future events (e.g. PA Health Symposium, Grand Rounds, MSH Research Support Scheme Grants Announcement event).
- A Final Report must be submitted to the MSH Research Support Scheme by 30 April 2021.

The Principal Investigator, all Co-Investigators, and the Principal Investigator's Head of Department (or line manager) must sign the Conditions of Award to accept this grant. Please email the certified Conditions of Award to the Centres for Health Research (CHR-RSS@health.qld.gov.au) **by midday Friday 14th December 2018.**

CERTIFICATION BY THE INVESTIGATIVE TEAM

We accept and agree to adhere to the Conditions of Award for a 2019 Metro South Health Research Support Scheme Project Grant funded by SERTA for the research study *entitled **"The TEACH-PD study: a Targeted Education ApproaCH to improve Peritoneal Dialysis outcomesargeted Education ApproaCH to improve Peritoneal Dialysis outcomes".***

Signatures:

(CI – Co-Investigator)

Principal Investigator name: Prof David Johnson Director of Nephrology

Principal Investigator signature: [signature] Date: 29/11/18

CI1 name: CARMEL HAWLEY

CI1 signature: [signature] Date: 3/12/18

CI2 name: DAVID MUDGE

CI2 signature: [signature] Date: 30/11/18

CI3 name: YEOUNGJEE CHO

CI3 signature: [signature] Date: 3/12/18

CI4 name: JEREMY FRAZIER

CI4 signature: [signature] Date: 4/12/2018

CERTIFICATION BY THE HEAD OF DEPARTMENT/DIVISION (IF THE PI IS HEAD OF DEPARTMENT, THIS SECTION MUST BE SIGNED BY THE LINE-MANAGER, OR SUITABLE AUTHORITY WITHOUT CONFLICT OF INTEREST)

I certify that I am prepared to have the research study entitled ***"The TEACH-PD study: a Targeted Education ApproaCH to improve Peritoneal Dialysis outcomesargeted Education ApproaCH to improve Peritoneal Dialysis outcomes"*** carried out in my Department/Division by Prof David W Johnson.

HOD or authorised delegate name: JUDY FLORES

Signature: ______________________ Date: __________

Official Title: ______________________

Grant Payment Request Form

Instructions:

1. Complete this form to initiate your grant payment.
2. Forward to your Business Manager/institutional Finance Director
    a. *this completed Grant Payment Request form* **AND**
    b. *a copy of the Letter of Award* **AND**
    c. *a signed copy of the Conditions of Award including Certification page with signatures.*

GRANT PAYMENTS WILL ONLY BE MADE FOLLOWING RECEIPT OF A COMPLETED GRANT PAYMENT REQUEST FORM (INCLUDING SIGNATURES)

SERTA awarded funds:

- must be deposited into a Metro South Health (MSH) research cost centre
- via direct journal.

I require a payment request be raised for the following MSH Research Support Scheme grant:

Principal Investigator	Prof David W Johnson
Project Title	**The TEACH-PD study: a Targeted Education ApproaCH to improve Peritoneal Dialysis outcomesargeted Education ApproaCH to improve Peritoneal Dialysis outcomes**
Grant Type	Project Grant
Funding Start Year	2019
Funding end date	31 December 2020
Amount	$99,995.32
Cost Centre #	
