# Supplementary material for: Multi-center, pragmatic, cluster-randomized, controlled trial of standardized peritoneal dialysis (PD) training versus usual care on PD-related infections (the TEACH-PD trial): trial protocol
Source: Trials. 2023 Nov 14;24:730. doi: 10.1186/s13063-023-07715-0 (PMC10647147; doi:10.1186/s13063-023-07715-0)
Supplement: Supplementary file 2 — Additional file 2. Funding documents. [file 13063_2023_7715_MOESM2_ESM.zip › Funding_MSHHSR1.pdf]

FUNDING AGREEMENT

BETWEEN:

METRO SOUTH HOSPITAL AND HEALTH SERVICE (ABN 86 834 068 616) Building 5, 2404 Logan Road, Eight Mile Plains, Queensland 4113 (**MSHHS**)

AND: THE UNIVERSITY OF QUEENSLAND ABN 63 942 912 684 a body corporate established under the University of Queensland Act 1998 (Qld), acting through the Australasian Kidney Trials Network

The party listed in Schedule 1 (**the Recipient**).

BACKGROUND

A. MSHHS is a public Hospital and Health Service established under the *Hospital and Health Boards Act 2011* (Qld) to deliver public sector health services in Queensland.

B. MSHHS has invited applications for a grant program established by MSHHS to support the Project and/or Activities (**the Grant Program**).

C. The Recipient has successfully applied for funding under the Grant Program.

D. The Recipient and MSHHS shall now collaborate on the Project and/or Activities on the terms set out in this Agreement.

DEFINITIONS

Activities means the activities set out in Item 4 of Schedule 1.

Agreement means this document and all annexures, attachments and schedules incorporated by reference.

Approved Purpose means the purpose that the Grant Funds are provided for, as set out in Item XX of Schedule 1.

Background IP means any Intellectual Property created prior to the commencement of the Project or independently of the Project, and which a Party contributes for the purpose of carrying out the Project.

Commencement Date means the date set out in Item 7 of Schedule 1.

Commercialisation means the provision of rights in Intellectual Property or services including the exploitation of Intellectual Property in exchange for any benefit, whether monetary or otherwise, but which does not include carrying out future research making use of such Intellectual Property under a competitive grants or public good scheme.

Confidential Information means any information passed by one Party to the other Party that is, or ought to reasonably be known to be, secret but does not include information that is:

(a) in the public domain;

(b) the Parties agree is not confidential;

(c) independently discovered or received by the other party without reference to the information disclosed under this Agreement; or,

(d) provided to the Recipient in accordance with consent of a Clinical Subject.

Grant Funds means the funding contributions that MSHHS is providing to the Recipient for the Approved Purpose, set out in Item 6 of Schedule 1.

Intellectual Property means all intellectual property rights, including but not limited to:

(a) trade and service marks (including goodwill in those marks), patents, inventions, discoveries, copyright, rights in circuit layouts, designs, domain names, registrable plant varieties or processes;

(b) any application or right to apply for registration of any rights referred to in paragraph (a); and

(c) all rights of a similar nature to any of the rights in paragraph (a) and (b) which may subsist anywhere in the world (including Australia), whether or not such rights are registered or capable of being registered.

Moral Rights has the same meaning as set out in the *Copyright Act 1968* (Cth).

Party means MSHHS or the Recipient or both as the context dictates and Parties shall have a corresponding meaning.

Personal Information is information or an opinion, including information or an opinion forming part of a database, whether true or not, about an individual whose identity is apparent, or can reasonably be ascertained, from the information or opinion.

Project means the funded project set out in Item XX of Schedule 1 and detailed in Schedule 2.

Project IP means all Intellectual Property created in the course of the Project but does not include Background IP.

Publish/Publication means to publish by way of a paper, article, manuscript, report, poster, internet posting, conference presentation, abstract, outline, video, instruction material or other disclosure, in printed, electronic, oral or other form relating to the Project.

Relevant Privacy Laws means the *Information Privacy Act 2009* (Qld), the *Public Health Act 2005* (Qld), the *Hospital and Health Boards Act 2011* (Qld) and any other legislation (including delegated and subordinate legislation such as regulations), code or guideline which applies in the jurisdiction where the Project is to be conducted and which relates to the protection of Personal Information.

OPERATIVE PROVISIONS

1 Provision of Funding

1.1 The Recipient has successfully applied for funding under the Grant Program.

1.2 MSHHS agrees to provide the Grant Funds to the Recipient for the Approved Purpose, on the terms and conditions of this Agreement.

1.3 The Recipient must carry out each Project and/or Activity in accordance with this Agreement.

1.4 In the event of any amendments to the Project and/or Activities, funding expenditure or unspent Grant Funds, the Recipient must seek the prior written approval of MSHHS before proceeding with the amendment.

1.5 MSHHS shall advance the Grant Funds on the Instalment Dates set out in Item 9 of Schedule 1.

1.6 If any of the Grant Funds provided to the Recipient by MSHHS remain unspent at the conclusion of the Project, then the Recipient shall remit the remaining Grant Funds back to MSHHS.

2 Term and termination

2.1 This Agreement commences on the Commencement Date and shall continue for the Funding Period.

2.2 A Party may terminate for breach of this Agreement provided that it gives notice of the breach and that the breach is not rectified within thirty (30) days of that notice.

2.3 In the event that the Recipient ceases to perform the Project and/or Activities for any reason, the Recipient must:

(a) notify MSHHS in writing of the cessation of the Project and/or Activities within five (5) business days of the date of cessation; and

(b) repay the balance of the Grant Funds remaining unused as at the date the Recipient ceased to perform the Project and/or Activities).

3 The Project

3.1 The Recipient agrees and acknowledges that the Project and/or Activities must be performed in compliance with:

(a) Schedule 2 to this Agreement;

(b) the terms and conditions of this Agreement; and

(c) all applicable local, state and federal laws, legislation, regulations, rules, by-laws, including without limitation the Relevant Privacy Laws.

3.2 The Parties shall exercise due skill, care and attention in carrying out the Project.

3.3 The Recipient shall provide the following reports:

(a) a quarterly report every three months during the term of the Project;

(b) a final report at the conclusion of the Project.

3.4 Each Report shall state the progress of the Project against the relevant milestones.

3.5 The Parties shall perform evaluation of the Project in accordance with the requirements for project evaluation set out in Schedule 2.

4 Intellectual Property

4.1 Nothing in this Agreement shall affect the ownership rights of Intellectual Property of either Party.

4.2 Each Party grants to the other Party a perpetual, irrevocable, royalty-free, worldwide, non-exclusive licence to use the Intellectual Property listed at Item 13 of Schedule 1 for the purposes of the Project and/or Activities.

5 Confidentiality

5.1 Except as is expressly contemplated in this Agreement, each Party:

(a) must not make public or disclose to any person any Confidential Information of the other Party; and

(b) must not use the Confidential Information of the other Party other than for the purposes permitted under this Agreement,

without the express prior written approval of the other Party.

5.2 MSHHS may disclose:

(a) the terms of this Agreement; and/or

(b) any other document or information in connection with this Agreement,

to the extent required to comply with any request, direction or order of any Queensland Government Minister, the State, or any government agency and its officers provided that in disclosing such information it makes the Recipient aware of its confidential nature.

5.3 Despite any other provision of this Agreement, MSHHS may disclose and/or publish:

(a) the terms of this Agreement; and/or

(b) any other document or information in connection with this Agreement,

to a member of the public that makes a request pursuant to the *Right to Information Act 2009* (Qld) to access the documents and/or information detailed in sub-clauses (a) and (b).

5.4 Each Party must:

(a) where requested to do so by the other Party (**the Discloser**), upon breach of its obligations of confidentiality and/or the Relevant Privacy Laws; and/or

(b) when this Agreement is otherwise terminated;

promptly return, destroy or erase any documents or records that contain the Discloser's Confidential Information (whether in electronic or

hard copy and in any storage device), as requested by the Discloser, except in relation to the retention and storage copies as required to comply with any applicable statutory record keeping legislation.

5.5 Either Party may disclose the Confidential Information of the other Party as required by law.

6 Publication

6.1 To the extent applicable, the Parties shall comply with the *National Health and Medical Research Council's Code for the Responsible Conduct of Research* in relation to all publications and authorship matters.

6.2 The Recipient shall acknowledge the support of MSHHS in all Publications.

7 Liability

7.1 The Recipient is solely liable for its acts and omissions in relation to the conduct of the Project and/or Activities.

7.2 The Recipient indemnifies MSHHS and its officers, agents, employees and contractors against all actions, claims, charges, costs (including legal costs on a full indemnity basis), expenses, losses, damages and other liability that they may sustain or incur, directly or indirectly, as a result or as a consequence of:

(a) a breach by the Recipient of this Agreement; and

(b) any negligent, unlawful, wilful or fraudulent act or omission of the Recipient and/or its officers, agents, employees or contractors in connection with this Agreement.

8 Dispute Resolution

8.1 A Party must not commence legal proceedings relating to this Agreement unless the Party wishing to commence proceedings has complied with this clause 8. However, this clause 8 will not apply where a Party seeks urgent interlocutory relief from a court.

8.2 The Parties will co-operate with each other and use their best endeavours to resolve by mutual agreement any differences between them and all other difficulties which may arise from time to time relating to this Agreement.

8.3 Where a Party wishes to raise a dispute under this Agreement, it must give notice to the other Party and the Parties must within thirty (30) days convene a meeting of their representatives who have authority to resolve such a dispute to discuss and seek to resolve the dispute in good faith.

9 General

9.1 This Agreement:

(a) contains the entire agreement of the parties; and

(b) supersedes all prior representations, conduct and agreements, with respect to its subject matter.

9.2 The laws of Queensland, Australia apply to this Agreement and each party irrevocably submits to the exclusive jurisdiction of the courts of Queensland, Australia and courts competent to hear appeals from those courts.

9.3 Any Special Conditions set out in Item 15 of Schedule 1 shall be deemed to be Operative Terms of this Agreement. Where the terms of the Special Conditions conflict with a provision contained in the body of this Agreement, the Special Conditions shall prevail.

9.4 In this Agreement, a capitalised term shall have the meaning defined in this Agreement.

EXECUTED AS AN AGREEMENT:

SIGNED for **METRO SOUTH HOSPITAL AND HEALTH SERVICE** by its authorised officer:

Signature of Authorised Officer

Professor Timothy Geraghty
Chair
Centres for Health Research

Full Name and Position of Authorised Officer

10/04/19

Date

In the presence of:

Signature of Witness

MARIA WOJCIECHOWSKI

Full Name and Position of Witness ESO.

SIGNED for **THE RECIPIENT** by its authorised officer:

Signature of Authorised Officer

Joe McLean
Director (Research Partnerships)

Full Name and Position of Authorised Officer

4 April 2019

Date

In the presence of:

Signature of Witness

Constantin de Boisseson
Administrative assistant

Full Name and Position of Witness

Schedule 1 – Specific Project Details

Item		Details
1	**Administering Institution**	**Legal Name:** Metro South Hospital and Health Service<br>**ABN:** 86 834 068 616<br>**Department:** Transformation and Innovation Collaborative<br>**Address:** PO Box 4043, Eight Mile Plains QLD 4113<br>**Contact Person:**<br>**Telephone:**<br>**Email:**
2	**Recipient**	**Legal Name:** The University of Queensland<br>**Trading Name:** acting through the Australasian Kidney Trials Network<br>**ABN:** 63 942 912 684<br>**Address:** Director, Research Partnerships<br>Room 376, Level 3<br>Global Change Institute<br>Staff House Road<br>The University of Queensland<br>Brisbane, 4072<br>AUSTRALIA<br>**Contact Person:** Director, Research Partnerships<br>**Telephone:** : +61 7 3365 3559<br>**Email:** director.partnerships@research.uq.edu.au
3	**Project Details**	**Project Name: T**argeted **E**ducation **A**pproa**CH** to Improve **P**eritoneal **D**ialysis Outcomes - **C**luster **R**andomised Controlled Trial (TEACH-PD CRCT)<br>**Project Details:** A pragmatic, registry-based, multi-centre, international, cluster-randomised controlled trial examining the use of TEACH-PD training modules for PD trainers and incident PD patients versus existing practices on the rate of PD-related infections<br>**Project Outcomes:** To determine whether implementation of standardised training modules based on ISPD guidelines targeting both PD trainers and patients results in a longer time to the composite end-point of exit site infections, tunnel infections and peritonitis in incident PD patients compared with existing training practices
4	**Activities**	TEACH-PD is a registry-based, pragmatic, multi-centre, multinational, cluster-randomised controlled trial (CRCT), randomising PD units to implement TEACH-PD training modules targeted at PD trainers and incident PD patients versus standard existing practices.<br>The modules will be implemented at PD units in Australia and New Zealand to formally evaluate whether, compared with standard care, a standardised training curriculum will reduce the rate of PD-related infections and improve technique survival, resulting in better outcomes for patients receiving PD and significant cost-savings to the community
5	**Authorised Purpose**	Funds are to enable the establishment of the TEACH-PD study by MSHHS at the Princess Alexandra Hospital
6	**Grant Funds**	A non-recurrent allocation of $120,000 (ex GST) per annum

7	**Commencement Date**	01 September 2018
8	**Funding Period**	Two financial years 2018-19 and 2019-20
9	**Funding Payment Terms**	**Number of Instalments**: 3<br>**Instalment Date(s):**<br>Within 30 days of a fully executed agreement: $120,000<br>30th September 2019: $60,000<br>30th March 2020: $60,000
10	**Insurance Requirements**	(a) Each party must, for as long as any obligations remain arising from this Agreement, effect and maintain valid, enforceable and adequate:<br>(i) public liability insurance of $10,000,000 or more per claim;<br>(ii) workers compensation insurance as required by statute;<br>(iii) general insurance in respect of all property (in which the Service Provider has an insurable interest) for the full reinstatement value, that is used in connection with the Services, including all buildings, fixtures and fittings and contents contained thereon or therein, against all loss and damage caused by or resulting from accident, fire, theft, malicious damage or storms and any other insurable risk which property of a similar nature is commonly insured against; and<br>(iv) professional indemnity insurance of $5,000,000 per claim.<br>(b) Proof of adequate levels of self-insurance or other protection by a party are acceptable as an alternative to the insurances required under this clause 11(a)
11	**Recipient Bank Account Details**	**Account Name:** The University of Queensland – No. 1 Account<br>**Account Number:** 10897870<br>**BSB:** 064-158<br>**Name of Bank:** Commonwealth Bank of Australia<br>**Bank Branch:**St Lucia
12	**Reporting**	Annually as requested by MSHHS
13	**Licenced Intellectual Property**	Nil
14	**Project Milestones**	First Queensland site open June 2019<br>6 Queensland sites open September 2019<br>100 participants recruited September 2019<br>350 participants recruited March 2020
15	**Special Conditions**	Nil

Schedule 2 – Project and/or Activities Plan

Funding will enable the development of trial resources, translation of study materials, project initiation, ongoing training and competency assessment of unit nurses, recruitment and data collection for over 300 PD patients and 50 nurses, and follow-up of participants over 2 years until 2020.
