# Supplementary material for: Multi-center, pragmatic, cluster-randomized, controlled trial of standardized peritoneal dialysis (PD) training versus usual care on PD-related infections (the TEACH-PD trial): trial protocol
Source: Trials. 2023 Nov 14;24:730. doi: 10.1186/s13063-023-07715-0 (PMC10647147; doi:10.1186/s13063-023-07715-0)
Supplement: Supplementary file 2 — Additional file 2. Funding documents. [file 13063_2023_7715_MOESM2_ESM.zip › Funding_NHMRC_APP1092957R1.pdf]

This snapshot report produced at: 03/06/2014 02:47 PM

Australian Government
National Health and Medical Research Council

Program - Assessor Snapshot Report: APP1092957

2014_Program Grants_funding_commencing_2016

**Application Title**	Better evidence, better care and outcomes for people with chronic kidney disease

**Administering Institution**	University of Sydney

**Indigenous**	No
**Guide to Peer-Review Areas**	Nephrology, Clinical Trials, Epidemiology

Chief Investigator	Pro-A: Primary Institution	Pro-A: Affiliated Institution(s)
(CIA) Jonathan Craig	University of Sydney	The Children's Hospital at Westmead<br>University of Sydney
(CIB) David Johnson	The University of Queensland	Metro South Hospital and Health Service
(CIC) Jeremy Chapman	University of Sydney	Westmead Hospital
(CID) Carmel Hawley	The University of Queensland	Metro South Hospital and Health Service<br>Queensland Health
(CIE) Stephen McDonald	The University of Adelaide	Central Adelaide Local Health Network Incorporated trading as Royal Adelaide Hospital<br>Central Northern Adelaide Renal and Transplantation Service

Contents

B-GP: Grant Proposal

B-CD: Career Disruption

B-CP: Participation

B-CG1: Collaborative Gain Part 1

B-CG2: Collaborative Gain Part 2

B-PPRC: Publications, Papers, Reports & Contribution

B-RA: Research Achievements

CV-QAP: Qualifications, Awards and Prizes

CV-EH: Employment History (last 5 years only)

CV-A: Appointments (last 5 years only)

CV-PM: Professional Memberships

CV-TPP: Translation into Policy / Practice (last 5 years only)

CV-JR: Editorial Responsibilities (last 5 years only)

CV-CN: Contributions to NHMRC (last 5 years only)

CV-CP: Conference Participation (last 5 years only)

CV-CE: Community Engagement (last 5 years only)

CV-SM: Supervision and Mentoring (last 5 years only)

CV-RF: NHMRC Research Funding (last 5 years only)

CV-ORF: Other Research Funding (last 5 years only)

CV-P: Patents

CV-W: Workload (Current)

B-GP: Grant Proposal

See separate attachment titled: APP1092957_Craig_Grant Proposal.pdf

B-CD: Career Disruption

Is any member of the CI team claiming a Career Disruption (CD)?

1. No – no member claiming a CD

B-CP: Participation

Provide a brief summary of the participation of each Chief Investigator in the broad research plan proposed in this application.

Professor Jonathan Craig Professor Craig is an internationally recognised clinical epidemiologist in CKD, and will be the fiscal and organisational guarantor for the study, acting within the governance arrangements provided by the University of Sydney. He is the Coordinating Editor of the Cochrane Renal Group, is the operational leader of KHA-CARI, and is on the Scientific Committee of Australasian Kidney Trials Network (AKTN). He is a paediatric nephrologist, and has a broad range of research skills including epidemiology, health economics, diagnostic test evaluation, systematic reviews and qualitative methods. In addition to the overall responsibility he will oversee the training/mentoring component, and lead many methods (research prioritisation, core outcomes, systematic reviews), and applied areas (paediatric, Indigenous health), particularly the development of the implementation theme. He will Chair the Program Management Committee.

Professor David Johnson Professor Johnson is an internationally recognised expert in clinical trials, peritoneal dialysis and CKD. As current Deputy-Chair of the AKTN, Professor Johnson will continue to help facilitate the design and conduct of all multi-centre, multi-national randomised controlled trials conducted under the oversight of the Program, and make substantial contributions to the applied cohort studies and epidemiological research using the platform provided by the ANZDATA registry. As a leader in peritoneal dialysis research, policy, guidelines and implementation, he will oversee this component of this Program. He will make a major contribution to all aspects of the Program but will make a critical contribution to the implementation theme given his leadership in KHA-CARI, ISPD, the Australasian Primary Care Collaborative and the Kidney Check Australia Taskforce (KCAT). He will be Deputy-Chair of the Program Management Committee, and provide ad-hoc support to CIA in oversight of the Program, where required.

Professor Jeremy Chapman Professor Chapman is widely recognised as a key, global figure in Transplantation, and has played a major role in defining policy and practice internationally. He will take responsibility for the applied area of kidney transplantation in the Program, across the three themes of identification, evaluation and implementation, and will also provide a major input into mentoring and career advice for early and mid-career researchers. His very strong international linkages in Nephrology and Transplantation will facilitate the impact of the Program globally.

Associate Professor Carmel Hawley A/Prof Hawley, through her leadership of the AKTN, has become an internationally recognised expert in the design and conduct of randomised trials in CKD, and this, combined with her formal qualifications in biostatistics and specific content expertise and research track record in haemodialysis, establishes her as a critical component of the Program. Her primary role under this Program of work will be to continue to oversee the design, conduct and management of all AKTN-coordinated trials, including providing guidance and mentorship to Early Career Researchers involved in the design and conduct of these trials. She will also take responsibility for the financial management of this grant, taking primary responsibility to ensure the budget is distributed and expended efficiently, and in line with both the Program's stated aims and the NHMRC funding rules.

Professor Stephen McDonald Professor Stephen McDonald is a nephrologist with internationally recognised expertise in the design and conduct of complex observational studies, particularly studies arising from large scale Registries of patients with end-stage kidney disease. This has developed in his role as ANZDATA Executive Officer, and in the proposed Program, he will take responsibility for the observational studies, which are especially critical for the identification and implementation themes. Led by Professor McDonald, the Program will enable extended follow up of trials through ANZDATA, together with the development of risk prediction and variation in care studies. He will also contribute to the implementation theme via collaborations with policy makers (AIHW and Organ Donation and Transplantation Authority).

B-CG1: Collaborative Gain Part 1

1. Integration and synergy of the research teams and program.

1.1: Describe the integration of the research teams and the program as a whole.

The Program team have a common goal: to reduce the burden caused by CKD. The Program combines the complementary clinical and academic skills, resources, policy and practice influence, and translational capabilities of 5 world-leading nephrologists and their teams, to form the first integrated Australian research Program focused on the prevention, timely detection and cost-effective management of chronic kidney disease (CKD). The Program draws together a wealth of clinical expertise, underpinned by methodological expertise in the areas of cohort studies, disease registries, data linkage, biostatistics, epidemiology, Cochrane systematic reviews and meta-analyses, mathematical modelling, design and conduct of multinational, investigator-initiated randomised controlled trials, health economic analyses, quality of life and patient preference evaluations, and evidence-based clinical practice guideline development and implementation. Research prioritisation will be shared across all platforms necessary for the discovery to translation pathway [systematic reviews (Cochrane Renal Group), cohort studies (ANZDATA), randomised trials (AKTN) and clinical practice guidelines (KHA-CARI)], enabling a coherent, strategic approach, targeted on issues important to patients and their caregivers. Operationally, the Program will be managed as a single, pooled budget, with shared infrastructure and data sharing, enabling the efficient and clean transfer of individual patient data between platforms. The team also bring together a broad and complementary network of national and international collaborators (eg AIHW, Australasian Primary Care Collaboratives, ISN, ISPD, Transplantation Society, KDIGO, KCAT). The integration of the team is evident in the frequency of co-publications, as provided below.

Co-publications 2009 to date

	JCr	DJ	JCh	CH	SM
Craig	295	31	35	3	7
Johnson	31	317	0	76	42
Chapman	35	0	175	0	8
Hawley 3	76	0	107	38	
McDonald	7	42	8	38	97

1.2: Describe productivity gains, including synergies and objectives that would not be possible with separate projects.

Program grant funding would enable a shared, prioritised approach to the problem of CKD, using a broad range of research methods, across the complete discovery to translation pathway, across all research platforms and across the broad spectrum of CKD stages. Fragmentation by any of these elements will not realise the primary outcome of the Program, a comprehensive evidence matrix for CKD. Productivity gains include:

1. Investigator-initiated, large-scale trials with leveraged funding: NHRMC Enabling Grant funding for AKTN ceases at the end of 2015. Incorporation of the AKTN into the Program will allow its continuation, promote expansion into kidney transplantation, provide the capacity to link with ANZDATA to determine long-term outcomes, and strengthen international collaborations to improve generalisability and impact. A Program grant will provide capacity to leverage substantial additional funding required to support definitive large-scale studies. Enabling funding ($3.29M) has facilitated the conduct of 8 multicentre trials, and leveraged an additional $11.6M from State Governments, Research Foundations, NGOs, and Industry.

2. Innovation through pilot funding of new ideas: 15% of the budget will be allocated for innovation funding. Such flexibility will allow the formulation of a suite of promising new interventions for established outcomes, a core outcomes set based on OMERACT methods (including under-recognised, patient-relevant outcomes), pilot studies of intervention strategies, and Cochrane Reviews.
3. Enhanced collaboration, shared infrastructure and personnel. Strong collaborations across the team have been forged through individual linkages and with Program support will now also be structured; funded staff will work across all platforms and themes, and infrastructures shared.
4. A stronger workforce: Flexible, but long-term support involving the full team, will promote the development of a substantial, multi-skilled group of future leaders.

2. Resource Management

2.1: Describe how teams and resources will be managed, including previous strategies and new collaborative arrangements.

The Program provides not only a new highly scientifically credentialed team, but one that is strong operationally and organisationally. As custodians of these 4 platforms, through tight but judicious operational control, substantial leveraged funding, and the in-kind support of the Australian nephrology community, they have produced substantial research output. The Program will provide a new element of oversight across the 4 platforms to ensure organisational coherence as well as at the project level. If funding is secured, a meeting of all CIs will be held in early 2015 to generate a detailed plan for commencement, including allocation of funding, based upon the established principles (consensus-based decision making, equity across all themes and platforms, a single pool, 15% unallocated for innovation (expansion or new projects)) and priorities for allocation (support for early career researchers, especially multidisciplinary researchers, projects which integrate among platforms and themes, potential for leveraged funding, evidence of patient-relevance). This will be followed up with scheduled, quarterly CI meetings to re-evaluate decisions based upon external (newly published research or collaborations) and internal factors (productivity based on agreed deliverables). A more comprehensive evaluation will occur annually using the same principles and processes, but involving the entire team of CIs and APs. All CIs will receive a monthly report regarding the financial status of the entire Program and for their areas of responsibility.

The major functional groups of the Program will develop and be responsible for an annual operational plan including comprehensive budget and a strategic 5-year plan. Milestones for each functional group will be required to ensure overall success and goals of the Program are met. Governance decisions will be made by consensus wherever possible but final responsibility and executive power will lie with CIA, and CIB in his absence.

2.2: Describe performance measures and/or milestones, and how grant funds and other resources will be shared, deployed, and redeployed if required.

The management and governance processes planned include monthly teleconferences, quarterly investigator meetings, and an Annual Scientific (and business) meeting of collaborators and research teams. In addition, teleconferences and face-to-face meetings will be facilitated as required for individual projects. This plan will be revised as required to ensure maximum efficiency and outputs. A collaborative matrix, integrating data from all collaborators with appropriate timelines for publications and other critical milestones, will be reviewed at each quarterly meeting. The goals, progress outcomes and budget status of individual projects will also be reviewed on a quarterly basis. More comprehensive assessment of all projects will occur on an annual basis at our planned annual meeting, and such reporting will also be required as part of progress reports to the NHMRC. Allocation and relocation of funds will be made by the collaborative team, directed by functional group leaders, with outcomes and impacts of completed studies considered in the future allocation of funds.

Performance will be assessed by metrics including:

- Publications: number, impact factor of the journal, citations, narrative of impact
- Leveraged funding: grant applications and successes (NHMRC, Industry, Philanthropy, NGOs, Government)

- Collaboration within the team: co-publication among the CIs, and broader team, joint grant applications and student supervision.
- Awards and Honours: including scholarships and fellowships to students, academic promotions.
- Capacity development: PhD and post-doc supervision (domestic and international), theses completed, career progression
- Translation: influence and impact on clinical guidelines, health policy and heath service delivery, new collaborations with policy makers locally and internationally
- Milestones: meeting budgetary targets for revenue and expenditure, and recruitment targets for clinical trials.

B-CG2: Collaborative Gain Part 2

3. Team Skills

3.1: Describe the team's skills that will be present within the program.

The team are all practising nephrologists, with a spread of clinical speciality areas, covering the full spectrum of CKD, from paediatrics (CIA), early CKD (all), haemodialysis (CID), peritoneal dialysis (CIB), and transplantation (CIC). The team are geographically diverse, covering NSW (Children's Hospital at Westmead, and Westmead Hospital), Queensland (Princess Alexandra Hospital), and South Australia (Royal Adelaide Hospital). Four of the five are epidemiologists, with one (CIA) holding a Personal Chair in Clinical Epidemiology. All are skilled analysts, with one (CID) having formal qualifications in biostatistics. They are recognised internationally in the science and conduct of clinical trials (CIA, CIB, CID), systematic reviews of multiple types (CIA), qualitative research (CIA), observational studies (CIB, CIE), and evidence-based clinical practice guideline development and implementation (CIA, CIB). They lead the major research and implementation platforms for CKD research and translation in Australia (AKTN – CIB, CID), ANZDATA (CIE), Cochrane Renal Group (CIA), CARI (CIA). They have also had publication and grant success in health economics (CIA, CIC). All have substantial linkages with policy makers, in transplantation (CIC, CIE – Organ and Tissue Authority), in kidney health (KHA – CIA, CIB), clinical networks (all), government (AIHW–CIB, CIE, PBAC and MSAC–CIA, Queensland Health–CIB, CID, NSW Health–CIA, CIC, SA Health-CIE)). They have different but overlapping national and international collaborations and research and clinical networks, including leadership positions of major international groups demonstrating international standing and organisational and operational skills (Co-Chair of the Cochrane Collaboration – CIA, International Society of Nephrology Councillor – CIB, International Society of Peritoneal Dialysis Councillor – CIB, President of the Transplant Society – CIC). They have all led or played major roles on studies with global impact.

4. Training, career development & mentoring

4.1: Briefly describe the track records of the CI's in the provision of research training, career development and mentoring within the broader research team and future opportunities for this to be provided in the Program proposal.

The team have an outstanding record in research training and career development, especially in the context of substantial clinical commitments. Collectively they have supervised 35 doctoral students to completion (17 current), 16 postdoctoral students, and many Masters/elective/honours students. They have trained multiple clinical research fellows from Canada (4), Italy (2), China (2), the UK (2), Belgium (1), Romania (1), France (1), Singapore (1), Thailand (1), Philippines (1), India (1), Jamaica (1), Norway (1), and the Netherlands (1). The European Clinical Practice guideline Program in CKD routinely send their research fellows to Australia for training in evidence based medicine and guideline development. CIA leads a formal program in Clinical Epidemiology that has 200 prevalent students. Demonstrating the success of the team in developing high quality researchers with a sustained commitment to research, many of the named APs having been mentored by the CI team, and many others have received post-doctoral support, and obtained senior clinical academic positions around Australia and Internationally. Many students have also won national (12) or international (3), young investigator awards. CIA was made an honorary member of the Italian Society Nephrology for capacity building. The effectiveness of these strategies is evident by the career trajectories of the mentored researchers (APs). For example, Dr Palmer has won multiple awards including the prestigious Rutherford Discovery Fellowship 2014-2019 from the Royal Society of New Zealand, and L'Oreal Women in Science award. Prof Strippoli was recently the youngest ever recipient of a Professorship in Italy, and A/Prof Tong was promoted from level B to D for her contribution to qualitative research. Capacity Development of early- and mid-career researchers will be a joint responsibility for all team members, but CIA and CIC will oversee this portfolio (for research training, and career progression respectively).

4.2: Describe the training and mentoring strategies that will be adopted in the Program.
In keeping with the track record of the Program team to date, training and mentoring will continue to be prioritised, but will be enriched because of the coordinated approach across the entire Program, across multiple sites, across multiple platforms, using the full skill set of the full Program team, and with shared supervision. It will be practically facilitated by personnel and project support, supplemented with formal (through University courses and units) and informal training and advice regarding question formulation, study design and analysis, grants, publication strategies, interviews, presentations, and promotions. All researchers will be encouraged to expand their skill set, incorporating all of the opportunities the Program will offer across the full range of clinical research designs on offer, including implementation. All CIs will be responsible for supervision and mentoring, with CIA taking overall responsibility for research training, CID for career advice and mentoring and CIC and CIE for methodological and statistical advice. All CIs will provide input in grant/promotion and fellowship applications, and will be available for input at the manuscript stage for all projects supported by the Program. Each participating centre provides weekly journal and research meetings that provide exposure to current methods and approaches. University coursework training will be offered and funded, and the full team, including the APs, are committed to providing a rich, multidisciplinary environment afforded by the Program. Specialised training will be offered where existing courses are not available (e.g. Jeremy Grimshaw for theory and practice of Implementation Research), and the strong multi-organisational international collaborations will be used for short and medium term placements of early and mid-career researchers.

5. Intellectual Exchange

5.1: Describe how intellectual exchange will be facilitated, within and beyond the program.
There is an established but largely project-based intellectual exchange between the CIs. Such exchange exists more formally in various organisations, including cross-membership in committees for the AKTN, CARI guidelines and ANZDATA Registry. This will be enhanced within the Program by:

- Regular meetings of the CI and full team. Monthly teleconferences will be conducted to discuss research directions, coordinate activities and identify areas for collaboration
- Utilise IT platforms to create a forum for informal collaboration among CIs and APs. This includes creating a website specific to the grant collaborators to allow sharing of results and presentations (within and outside of the monthly meetings). The public component of this website will outline the current research directions and specific projects, together with an invitation to comment.
- Annual Scientific meeting. An annual one-day meeting for CIs and APs will be conducted to review results arising from the grant, areas of interaction and plan strategies for the forthcoming year, including existing and new collaborations, and opportunities to influence policy and practice. This will incorporate a keynote lecture from an international collaborator focussing on a potential area of innovation.
- Within the first year of the Program, we will convene a specific one-day meeting with stakeholders (nephrology, government, industry) to identify areas for where existing or planned research can inform current and future policy.
- Strengthen existing international collaborations (groups and individuals) and target formal international scientific meetings for presentation of results, and ad-hoc, Program specific meetings, to promote discussion in the broader research environment and the exchange of ideas into the policy sector.

B-PPRC: Publications, Papers, Reports & Contribution

Provide comment on the most significant publications, papers, reports and other contributions.

Team Member: **(CIA) Jonathan Craig**

Last 5 years: • Craig JC et al. Prevention of Recurrent Urinary Tract Infection in Children with Vesicoureteric Reflux and Normal Renal Tracts (PRIVENT) Investigators. Antibiotic prophylaxis and recurrent urinary tract infection in children. New England Journal of Medicine, 2009; 361(18):1748-59 [194 cites] The first adequately powered, placebo-controlled trial that demonstrated that antibiotic prophylaxis provides real, but small benefits, irrespective of underlying renal tract malformations.

• Cooper BA, Branley P, Bulfone L…Craig JC...Johnson DW et al. A randomized, controlled trial of early versus late initiation of dialysis. New England Journal of Medicine 2010; 363(7):609-19. [303 cites]. The only trial of this nature conducted, showing the early dialysis is not beneficial for CKD patients, which has enormous clinical, policy and financial implications.

• Baigent C, Landray MJ, Reith C…Craig J et al. The effects of lowering LDL cholesterol with simvastatin plus ezetimibe in patients with chronic kidney disease (Study of Heart and Renal Protection): a randomised placebo-controlled trial. The Lancet 2011; 377(9784): 2181-92 [704 cites] showed that lipid lowering improves cardiovascular outcomes for people with CKD

• Palmer SC, Hayen A, Macaskill P, Craig JC et al, Elder GJ, Strippoli GF. Serum levels of phosphorus, parathyroid hormone, and calcium and risks of death and cardiovascular disease in individuals with chronic kidney disease: a systematic review and meta-analysis. JAMA 2011; 305(11):1119-27. [174 cites] A complex prognostic review showing that these biomarkers are not associated with important clinical outcomes, contrary to widespread opinion.

• Palmer SC, Navaneethan SD, Craig JC, Johnson DW et al. Meta-analysis: erythropoiesis-stimulating agents in patients with chronic kidney disease. Annals of Internal Medicine 2010; 153(1):23-33. [147 cites] This conclusively demonstrated the hazards of normalization of haemoglobin values in people with CKD.

Over the course of your career: • Tong A, Sainsbury P, Craig JC. Consolidated criteria for reporting qualitative research: a 32-item checklist for interviews and focus groups. International Journal for Quality in Health Care 2007; 19:349-57. [567 cites] has become the standard method for reporting qualitative research across all disciplines.

• Schünemann HJ, Oxman AD, Brozek J... Craig JC et al GRADE: grading quality of evidence and strength of recommendations for diagnostic tests and strategies. British Medical Journal 2008; 336:1106-10. [400 cites] GRADE has become the standard approach to guideline development and this is the paper that outlines the approach to diagnosis.

• McDonald S, Craig JC. Long-term survival of children with end-stage renal disease. New England Journal of Medicine 2004; 350(26):2654-62 [331 cites] become the standard reference for describing the outcome for children with ESKD.

• Strippoli GFM, Craig JC, Schena FP. The number, quality, and coverage of randomized controlled trials in nephrology. Journal of the American Society of Nephrology. 2004;15(2):411-419. [180 cites] The first to describe the poor state of trials in nephrology, underpinning the development of new networks and strategies.

• Hodson E…Craig JC. Antiviral medications to prevent cytomegalovirus disease and early death in recipients of solid-organ transplants: a systematic review of randomised controlled trials. The Lancet 2005; 365(9477):2105-15 [248 cites] demonstrated that antivirals are effective and safe

• Caldwell P..Craig JC. Clinical trials involving children. The Lancet 2004; 364:803-11 [215 cites] Invited review outlining the key differences about clinical trials in children.

Team Member: **(CIB) David Johnson**

Last 5 years: • Cooper BA...Craig JC...Johnson DW et al. A Randomized, Controlled Trial of Early versus Late Initiation of Dialysis. NEJM, 2010; 363(7): 609-19 [303 cites]. Found no advantage to commencing dialysis early and is widely regarded as one of the most influential trials in Nephrology.

- Li PK...Johnson DW et al. Peritoneal dialysis-related infections recommendations: 2010 update. Peritoneal Dialysis International, 2010; 30(4): 393-423 [399 cites]. This is the peak global clinical guideline for the management of peritonitis in peritoneal dialysis patients, endorsed by the International Society of Peritoneal Dialysis. It has been translated into Mandarin, French, Spanish and Portuguese. CIB has published more original PD clinical research articles in the last 5 years than any other researcher in the world, as well as those on PD outcomes and ISPD guidelines.
- McDonald SP...Johnson DW et al. Relationship between dialysis modality and mortality. JASN, 2009; 20(1):155-63 [145 cites]. Examines a classic nephrological question using mutiple statistical methods, and showed that the comparison of modalities is not as simple as often portrayed, but in fact depends on patient comorbdities and followup window. Frequently cited as a methodological reference.
- Palmer S, Craig JC, Johnson DW et al. Meta-analysis: Erythropoiesis-stimulating agents in patients with chronic kidney disease. Annals of Internal Medicine, 2010; 153(1): 23-33 [147 cites]. Conclusively demonstrated the hazards of normalization of haemoglobin values in people with CKD.
- Johnson DW et al. Effects of biocompatible versus standard fluid on peritoneal dialysis outcomes – the balANZ trial. JASN, 2012; 23(6): 1097-107 [9 cites]. RCT showing that biocompatible PD fluids preserved renal and peritoneal membrane function and reduced peritonitis rates and severity. It has been referred to in various editorials as "the best randomized trial of biocompatible PD fluids" and "a game-changer."

Over the course of your career: In addition to those during the last 5 years:

- Strippoli G,...Johnson DW,...Craig JC et al. Effects of statins in patients with chronic kidney disease: meta-analysis and meta-regression of randomised controlled trials. BMJ, 2008; 336(7645): 645-51 [301 cites]. Demonstrated the lipid concentration and cardiovascular endpoint reductive benefits of statins in a CKD population.
- Vesey D,...Johnson DW. Erythropoietin protects against ischaemic acute renal injury. NDT, 2004; 19(2): 348-55 [257 cites]. The first to demonstrate that erythropoietin exhibits novel and profound renoprotection against ischaemic acute kidney injury.
- Mathew T;...Johnson D et al. Chronic kidney disease and automatic reporting of estimated glomerular filtration rate: A position statement. MJA, 2005; 183(3): 138-41 [132 cites], and the revision MJA, 2007; 187(8) [96 cites]. Resulted in the universal adoption of automated laboratory reporting of eGFR, making it one of the most successful opportunistic CKD screening programs in the world.
- Johnson DW et al. A randomized controlled trial of topical exit site mupirocin application in patients with tunnelled, cuffed haemodialysis catheters. Nephrology Dialysis Transplantation, 2002; 17(1): 1802-7 [151 cites]. Based on this work, exit site mupirocin prophylaxis is now recommended as the standard of care by many international guidelines.
- Johnson DW,…Hawley CM et al. Randomized, controlled trial of topical exit-site application of honey (Medihoney) versus mupirocin for the prevention of catheter-associated infections in hemodialysis patients. JASN, 2005; 16(5): 1456-62 [118 cites]. Demonstrated topical exit site honey was safe, cheap and as effective as mupirocin prophylaxis for preventing catheter-related bacteraemia, without antimicrobial resistance. Many units globally have now adopted MediHoney prophylaxis.

Team Member: (CIC) Jeremy Chapman

Last 5 years: • Kasiske BL,...Chapman JR, Craig JC et al. KDIGO clinical practice guideline for the care of kidney transplant recipients: a summary. Kidney international 2010; 77 (4), 299-311 [120 cites]. This is a set guidelines developed by the collaborative effort of the international transplant society, which aim to improve the care of kidney transplant recipients.

- Webster AC,...Chapman JR, Craig JC et al. Interleukin 2 receptor antagonists for kidney transplant recipients. Cochrane Database Syst Rev 2010 [110 cites]. This systematic review conclusively demonstrates IL-2 receptor antagonists are effective as an induction therapy in preventing acute rejection in kidney transplantation compared to T-cell depleting agents. This has led to change in clinical practice and guideline recommendations in Australia and worldwide.
- Kuypers DRJ,...Chapman JR et al. Consensus report on therapeutic drug monitoring of mycophenolic acid in solid organ transplantation. CJASN 2010; 5(2), 341-358 [88 cites]. Provides important information to transplant practitioners on the clinically relevant pharmacokinetic characteristics of mycophenolic acid and to rationalize their use and target exposure ranges in varies types of organ transplantation.
- Wong G, Hayen A,…,Chapman JR, Craig JC. Association of CKD and cancer risk in older people. JASN 2009; 20(6): 1341-1350 [71 cites]. This is the first observational study that cancer risk is increased in people with mild to moderately reduced kidney function.

• van Leeuwen M, Webster AC,...Chapman JR et al. Effect of reduced immunosuppression after kidney transplant failure on risk of cancer: population based retrospective cohort study. BMJ 2010; 11(340):c570 [39 cites]. The first observational study that demonstrates the risk of cancer reduces after immunosuppression withdrawal in kidney transplant recipients who failed their transplants.

Over the course of your career: • Nankivell B,...Chapman, JR. The natural history of chronic allograft nephropathy. New England Journal of Medicine 2003; 349(24): 2326-2333 [1315 cites]. The first observational study demonstrating the trajectory and the risk factors that predict the decline in renal function among kidney transplant recipients.

• Vajdic C, McDonald SP,…Chapman JR. Cancer incidence before and after kidney transplantation. JAMA 2006; 296 (23): 2823–2831 [416 cites]. The first observational study that shows the relative increased risk of cancer among transplanted, dialysis and pre-dialysis patients compared to the general population in Australia and New Zealand.

• Nankivell B,...Chapman J. Calcineurin inhibitor nephrotoxicity: longitudinal assessment by protocol histology. Transplantation 2004; 78(4): 557-565 [365 cites]. The first prospective observational study that shows calcinuerin inhibitors toxicity is one of the major primary causes of long-term kidney graft dysfunction.

• Webster A,...Chapman, JR, Craig JC. Tacrolimus versus ciclosporin as primary immunosuppression for kidney transplant recipients: meta-analysis and meta-regression of randomised trial data BMJ 2005; 331(7520): 810 [311 cites]. Provides confirmatory evidence that tacrolimus is more effective in preventing acute rejection and longer-term graft loss among recipients of kidney transplant.

• Chapman JR et al. Reversibility of cyclosporine nephrotoxicity after three months' treatment. Lancet 1985; 1(8421): 128-130. [136 cites] The first RCT that demonstrated that the effects of Cyclosporine on both the GFR and tubular functions are largely reversible after withdrawal of the treatment.

• Nankivell B,...Chapman JR. Predicting glomerular filtration rate after kidney transplantation. Transplantation 1995; 59(12): 1683–1689 [226 cites]. Provides an accurate formula that estimates the GFR in transplanted patients.

Team Member: (CID) Carmel Hawley

Last 5 years: • Johnson DW,…Hawley CM., HONEYPOT Study Collaborative Group. Antibacterial honey for the prevention of peritoneal dialysis related infections (HONEYPOT): a randomised trial. Lancet Infect Dis 2014; 14(1): 23-30 [1 cite]. A potentially practice-changing publication, and also the first AKTN trial to be completed and published.

• Brimble KS,…Hawley CM,…Macdonald SP, et al. Estimated GFR reporting influences recommendations for dialysis initiation. J Am Soc Nephrol 2013; 24(11): 1737-1742. Will play an important role in influencing clinical practice; this paper examined the role of reporting of GFR in decision making by clinicians, which provides an important message for clinical practice.

• Morrish AT, Hawley CM, Johnson DW, et al. Establishing a kidney trials network in Nephrology: the Australasian Kidney Trials Network. Kidney Int 2014; 85(1); 23-30. An important article as it highlights AKTN procedures and progress, and put the Network on the international stage.

• McMahon EJ,…Hawley CM,…Johnson DW, et al. A randomized trial of dietary sodium restriction in CKD. J Am Soc Nephrol 2013; 24(12): 2096-2103 [6 cites]. It importantly re-energises the argument for sodium restriction as a therapy in improving outcomes in Chronic Kidney Disease.

• Muir CA,…Hawley CM, et al. Buttonhole cannulation: clinical outcomes in a home haemodialysis cohort and systematic review. CJASN 2014; 9(1): 110-119 [1 cite]. Reviewed and appraised the published data on the hazards associated with buttonhole cannulation, and therefore has the potential to influence clinical practice.

Over the course of your career: • Rigby RJ, Hawley CM. Sclerosing peritonitis: the experience in Australia. Nephrol Dial Transplant 1998; 13(1):154-159 [287 cites]. A landmark paper in defining epidemiology of sclerosing peritonitis.

• Johnson DW,…Hawley CM. A comparison of dialysis versus renal transplantation on the survival of elderly uraemic patients. Transplantation 2000; 69(5): 794-9 [107 cites]. First paper to define the magnitude of the increased risk of death in remaining on dialysis as compared with proceeding to transplantation.

• Roger SD,…Hawley CM et al. Effects of early and late intervention with epoetin α on left ventricular mass among patients with chronic kidney disease (stage 3 or 4): results of a randomized clinical trial. 2004; 15(1): 148-156 [212 cites]. This study did not demonstrate an effect of anaemia correction on left ventricular mass and was the first study to examine this end-point with this therapy.

- Ramsay M, Fryer AA, CM Hawley et al. Non melanoma skin cancer risk in the Queensland renal transplant population. British Journal of Dermatology 2002; 147(5): 950-956 [162 cites]. This was a seminal paper defining the skin cancer risk associated with renal transplant recipients.
- Johnson DW,…Hawley CM et al. Randomized, controlled trial of topical exit-site application of honey (Medihoney) versus mupirocin for the prevention of catheter-associated infections in hemodialysis patients. JASN, 2005; 16(5): 1456-62 [118 cites]. Demonstrated topical exit site honey was safe, cheap and as effective as mupirocin prophylaxis for preventing catheter-related bacteraemia, without antimicrobial resistance. Many units globally have now adopted MediHoney prophylaxis.

Team Member: **(CIE) Stephen McDonald**

Last 5 years: • McDonald SP, Marshall MR, Johnson DW, Polkinghorne KR. Relationship between Dialysis Modality and Mortality. J Am Soc Nephrol 2009;20:155-63 [145 cites]. Examines a classic nephrological question using mutiple statistical methods, and showed that the comparison of modalities depends on patinet comorbdities and followup window. Frequently cited as a methodological reference.
- Sparke C,….McDonald S. Estimating the total incidence of kidney failure in Australia including individuals who are not treated by dialysis or transplantation. Am J Kidney Dis 2013; 61, 413-9 [8 cites]. Using linkage between the ANZDATA Registry and the national death index, demonstrated that many people with end-stage kidney disease do not receive dialysis or transplantation, but die of untreated ESKD.
- Marshall MR, Hawley CM,…McDonald SP. Home hemodialysis and mortality risk in Australian and New Zealand populations. Am J Kidney Dis 2011; 58: 782-793, 2011 [41 cites]. Used a technique not previously applied in nephrology (marginal structural models) to deal with the complex causal relationships between dialysis comorbidity and time-varying confounders.
- Grace BS,…McDonald SP. Transplantation rates for living- but not deceased-donor kidneys vary with socioeconomic status in Australia. Kidney Int 2013; 83:138-45 [7 cites]. Explores access to kidney transplantation by socio-economic status, for the first time in Australia. Despite a universal health care system, there is a clear gradient in living donor transplantation.
- Grace BS,…McDonald SP. Socioeconomic Differences in the Uptake of Home Dialysis. Clin J Am Soc Nephrol 2014; 9(5):929-35 [1 cite]. Describes systematic differences in the uptake of home therapy modalities. Despite the advantage of home-based modalities, uptake of peritoneal dialysis was inversely related to socio-economic status.

Over the course of your career: These papers describe the use of Registry (ANZDATA) data, in particular utilising data linkage and long term follow-up.
- McDonald SP, Russ GR. Survival of recipients of cadaveric kidney transplants compared with those receiving dialysis treatment in Australia and New Zealand, 1991-2001. Nephrol Dial Transplant 2002; 17: 2212-9 [103 cites]. This paper is an early application of more sophisticated statistical techniques to the ANZDATA Registry, describing benefits of transplantation over dialysis.
- McDonald SP, Collins J, Johnson, DW. Obesity is associated with worse peritoneal dialysis outcomes in the Australian and New Zealand patient populations. J Am Soc Nephrol 2003; 14: 2894-2901 [116 cites]. Uses ANZDATA Registry data to contradict the paradigm of better survival (among dialysis patients) associated with obesity.
- McDonald SP, Craig JC. Long term survival of children with end-stage renal disease. N Engl J Med 2004; 350: 2654-2662 [331 cites]. The definitive paper describing long term outcomes of children after treatment with dialysis and transplantation.
- Vajdic CM, McDonald SP,…Chapman JR et al. Cancer Incidence Before and After Kidney Transplantation. JAMA 2006; 296: 2823-2831 [463 cites]. Outcome of data linkage between the ANZDATA Registry and National Cancer Registry Clearing House. This is the definitive manuscript about risk of cancer post-transplant, an example of the benefits of linkage.
- Marshall MR,…McDonald SP. Associations of hemodialysis dose and session length with mortality risk in Australian and New Zealand patients. Kidney Int 2006; 69: 1229–1236 [103 cites]. Demonstrates increased mortality with haemodialysis session lengths <4 hours.
- Polkinghorne KR, McDonald SP et al. Vascular access and all-cause mortality: a propensity score analysis. J Am Soc Nephrol 2004; 15: 477-486 [170 cites]. Early demonstration of increased mortality risk associaetd with graft and central catheter use in haemodiaylsis patients.

B-RA: Research Achievements

See separate attachment titled: APP1092957_Craig_Research Achievements.pdf

CV-QAP: Qualifications, Awards and Prizes

Team Member	Type	Title	Institution/ Organisation	Year
(CIA) Jonathan Craig	PhD		University of Sydney	1997
	Masters	Clinical Epidemiology	University of Sydney	1997
	Graduate Diploma	Child Health	University of Otago	1989
	Degree	Bachelor of Medicine, Bachelor of Surgery	University of Otago	1986
	Award	National Kidney Foundation International Distinguished Medal for 2010.	National Kidney Foundation of the US	2010
	Award	Antecedents of Renal Disease in Aboriginal Children	National Health and Medical Research Council	2009
	Award	TJ Neale Award for Outstanding Contribution to Nephrological Science	Australian & New Zealand Society of Nephrology	2009
	Award	Kidney Health Australia Clinical Research Award	Kidney Health Australia	2008
	Award	Honorary Membership awarded by the Italian Society of Nephrology for Excellence in Capacity Building	Italian Society of Nephrology	2004
	Award	The Royal Alexandra Hospital for Children Young Investigator’s Award	The Royal Alexandra Hospital for Children, Sydney	1994
	Prize	Sir Thaddeus McNaughton Surgical prize	University of Otago	1986
	Other	Fellow	Royal Australasian College of Physicians	1994
(CIB) David Johnson	PhD	PhD - Medicine	University of Sydney	1998
	Degree	Doctor of Medicine (Research)	University of Queensland 2013	2013
	Degree	MB, BS (First Class Honours, University Gold Medal)	University of Queensland	1987
	Award	International Distinguished Medal	National Kidney Foundation, New York, USA	2014

	Award	Kidney Health Australia Best Clinical Science Award	Australian and New Zealand Society of Nephrology	2013
	Award	Public Service Medal for outstanding public service to Queensland Health, particularly to research into the early detection and management of chronic kidney disease.	Governor-General of the Commonwealth of Australia	2011
	Award	Australian of the Year Award (Queensland Finalist)	Australia Day Council	2009
	Award	TJ Neale Award for Outstanding Contribution to Nephrological Science	Australian and New Zealand Society of Nephrology	2005
	Award	KHA Clinical Nephrology Research Award	Kidney Health Australia & The Australia and New Zealand Society of Nephology	2005
	Award	Amgen Basic Science Research Award	Australia and New Zealand Society of Nephrology	2005
	Award	Clinical Nephrology Research Awards	Australian Kidney Foundation & The Australia and New Zealand Society of Nephrology	2001
	Award	Australian Kidney Foundation Clinical Nephrology Research Award	Australian and New Zealand Society of Nephrology	2000
	Award	Excellence Award for Contribution to Nephrology Service	Queensland Health	2000
	Award	Young Investigator Award	Australian and New Zealand Society of Nephrology	1998
	Award	Young Investigator -XIVth International Congress of Nephrology	Australian and New Zealand Society of Nephrology	1997
	Award	Best Oral Presentation - Annual General Meeting	Royal North Shore Hospital and University of Technology Sydney	1997
	Award	Beryl and Jack Jacobs Postgraduate Research Award	Royal North Shore Hospital	1997
	Award	Best Oral Presentation Award - Annual General Meeting	Royal North Shore Hospital and University of Technology Sydney	1996
	Award	Best Poster ANZSN Annual Scientific Meeting	Australian and New Zealand Society of Nephrology	1996
	Award	New Investigator Award	Royal North Shore Hospital	1996
	Award	Young Investigator Awards	Australian and New Zealand Society of Nephrology	1996

	Award	Best Poster - Annual General Meeting	Royal North Shore Hospital and University of Technology	1995
	Award	Royal Australasian College of Physicians' Advanced Trainee Research Awards	Royal Australasian College of Physicians	1995
	Award	Australian and New Zealand Society of Nephrology Young Investigator Awards	Australian and New Zealand Society of Nephrology	1995
	Award	Royal Australasian College of Physicians' Advanced Trainee Research Awards	Royal Australasian College of Physicians	1994
	Award	University Gold Medal	University of Queensland	1987
	Prize	Peter Bancroft Prize for best PhD thesis	University of Sydney	2000
	Prize	The Elsie Jamieson Memorial Prize for excellence in medical research	Queensland Branch of Royal Australasian College of Physicians	1993
	Prize	The Royal Brisbane Hospital Medical Staff Association Prize for Medical Research	Queensland Health	1993
	Prize	The Modecon Prize in Radiology	The University of Queensland Medical School	1987
	Prize	The Kenneth Wilson Prize (Child Health)	The University of Queensland Medical School	1987
	Prize	The John F. Dunkley Memorial Prize (Obstetrics and Gynaecology)	The University of Queensland Medical School	1987
	Prize	The Elsie Butler Wilkinson Memorial Prize in Obstetrics and Gynaecology	The University of Queensland Medical School	1987
	Prize	The H.G. Windsor Prize (Surgery)	The University of Queensland Medical School	1986
	Prize	The Neville Sutton Bursary (Surgery & Anatomy)	The University of Queensland Medical School	1986
	Prize	John Wienholt Memorial Scholarship in Anatomy and Physiology	The University of Queensland Medical School	1984
	Other	Fellowship of The Royal Australasian College of Physicians	The Royal Australasian College of Physicians	1994
(CIC) Jeremy Chapman	Masters	MA	Cambridge University	1979
	Degree	MB BCh	Cambridge University	1978

	Degree	BA	Cambridge University	1975
	Award	David Hume Award, National Kidney Foundation of America	National Kidney Foundation of America	2011
	Award	2010 Asturias Foundation Award: International Cooperation	Prince of Asturias Foundation	2010
	Award	Order of Australia medal (OAM)	Australian Government	2003
	Other	Honorary Membership	TSANZ	2011
	Other	FRCP	Royal College of Physicians	1995
	Other	FRACP	The Royal Australasian College of Physicians	1989
	Other	MD	Cambridge University	1987
	Other	MRCP(UK)	Membership of the Royal Colleges of Physicians of the United Kingdom	1981
(CID) Carmel Hawley	Masters	M. Med Sci (Medical Statistics)	University of Newcastle	2002
	Degree	Bachelor of Medicine & Surgery with 1st Class Honours (MBBS)	University of Queensland	1980
	Award	Award for services to the management of patients with kidney disease	Brisbane Electorate Australia Day Award	2000
	Award	University Medal for MBBS degree	University of Queensland	1981
	Award	Jean & Joyce Stobo Memorial prize	University of Queensland	1981
	Award	Charles Mitford Lilley Memorial Prize in Surgery	Univeristy of Queensland	1981
	Award	William Nathaniel Robertson Medal	Univeristy of Queensland	1981
	Award	Dux of School	Our Lady of Good Counsel College, The Range, Rockhampton, Queensland	1976
	Award	Dux of School	Marion Secondary School for Girls, Rockhampton, Queensland	1974

	Other	Fellowship of The Royal Australasian College of Physicians - Nephrology	Royal Australasian College of Physician	1988
	Other	Research Scholarship	Australian Kidney Foundation Research Scholarship	1988
(CIE) Stephen McDonald	PhD	Renal Disease, Cardiovascular Disease and Shared Risk Factors in Remote Aboriginal Communities	Flinders University of SA	2004
	Degree	MBBS(Hons)	University of Adelaide	1991
	Diploma	Fellow, Royal Australasian College of Physicians		1997
	Prize	KHA award for best clinical research presentation, ANZSN Annual Scientific Meeting	Kiodney Health Australia	2006
	Prize	The Robert and Lynda Stamp Prize (Community Medicine)	University of Adelaide	1990
	Prize	The Ian Furler Prize in Obstetrics & Gynaecology	University of Adelaide	1989
	Prize	The Keith Sheridan Prize (Overall 5th year MBBS results)	University of Adelaide	1989
	Prize	The Ruth Heighway Prize (Obstetrics)	University of Adelaide	1989

CV-EH: Employment History (last 5 years only)

Team Member	Employer	Job Title	Type	Period	Current
(CIA) Jonathan Craig	Royal Prince Alfred Hospital, Sydney	Visiting Medical Practitioner (Honorary Consultant Medical Officer)		2011 -	Yes
	The University of Sydney	Director, Clinical Epidemiology Program	Part time	2008 -	Yes
	The University of Sydney	Professor in Paediatrics and Child Health	Part time	2007 -	Yes
	University of Sydney	Professor of Clinical Epidemiology (Personal Chair), School of Public Health	Part time	2007 -	Yes
	Royal Prince Alfred Hospital	Visiting medical practitioner (Honorary Consultant Medical Officer)	Part time	2004 -	Yes
	The Children's Hospital at Westmead	Senior Staff Specialist in Nephrology	Part time	2002 -	Yes
	Westmead Hospital	Visiting Medical Practitioner, Renal Medicine	Part time	1998 -	Yes
	The Children's Hospital at Westmead	Head of Clinical Research, Centre for Kidney Research	Part time	1998 -	Yes
(CIB) David Johnson	Diamantina Health Partners	Theme Leader, Chronic Disease and Ageing	Full time	2013 -	Yes
	Queensland Health	Chair, Statewide Renal Clinical Network	Part time	April 2008 - August 2013	No

	Queensland Health	Chair, Division of Medicine, Princess Alexandra Hospital	Part time	May 2006 - April 2009	No
	Royal Australian College of Physicians	Lecturer Basic Physician Trainees Course governed by Queensland State Committee Branch	Part time	1998 - 2011	No
	Queensland Health	Medical Director, Queensland Renal Transplant Service	Full time	July 2011 -	Yes
	University of Queensland	Deputy Chair, Australasian Kidney Trials Network	Part time	January 2009 -	Yes
	University of Queensland	Director of Research, Centre for Kidney Disease Research	Part time	2009 -	Yes
	University of Queensland	Professor of Population Health	Full time	2007 -	Yes
	University of Queensland	Professor of Medicine	Full time	October 2003 -	Yes
	Queensland Health	Director, Metro South and Ipswich Nephrology and Transplant Services	Full time	November 2002 -	Yes
	Queensland Health	Director of Renal Medicine, Princess Alexandra Hospital, Brisbane, Australia	Full time	2002 -	Yes
	Queensland Health	Consultant Renal Physician (Full-Time), Princess Alexandra Hospital, Brisbane, Qld.	Full time	1998 -	Yes
	Queensland Health	Head of Renal Research Laboratory, Princess Alexandra Hospital	Part time	1998 -	Yes

(CIC) Jeremy Chapman	Division of Medicine and Cancer, Western Sydney Local Health District, Westmead Hospital	Clinical Director	Full time	2013 -	Yes
	Acute Interventaionl medicine, Sydney West Area health Service, Westmead Hospital	Network Director	Full time	2005 - 2013	No
	University of Sydney, Faculty of Medicine	Clinical Professor	Full time	2003 -	Yes
	Tissue Typeing Laboratory, Australian Red Cross Blood Service	Clinical Medical Director	Full time	1998 -	Yes
	Australian Bone Marrow Donor Registry	Chairman	Full time	1990 -	Yes
	Children's Hospital at Westmead	Honorary Renal Physician	Full time	1988 -	Yes
	National Pancreas Transplant Unit, Westmead Hospital	Physician	Full time	1987 -	Yes
(CID) Carmel Hawley	School of Medicine, University of Queensland	Chair, Operations Secretariat, Australasian Kidney Trials Network	Full time	2005 -	Yes
	School of Population Health, University of Queensland	Associate Professor	Part time	2004 - 2010	No
	Diabetes Australia	Grant Reviewer	Part time	1999 - 2009	No
	University of Queensland	Associate Professor of Medicine	Full time	January 2004 -	Yes

	University of Queensland	Examiner	Part time	2000 -	Yes
	Kidney Health Australia	Grant Reviewer	Part time	1999 -	Yes
	National Health and Medical Research Council of Australia	Grants Reviewer	Part time	1999 -	Yes
	Princess Alexandra Hospital	Supervisor	Full time	1998 -	Yes
	Princess Alexandra Hospital	Convenor	Part time	1998 -	Yes
	Royal Australasian College of Physicians	Lecturer	Part time	1998 -	Yes
	Princess Alexandra Hospital, Brisbane	Clinical Director of Haemodialysis	Full time	1997 -	Yes
	Princess Alexandra Hospital	Consultant Nephrologist	Full time	January 1989 -	Yes
	University of Queensland	Lecturer/tutor	Part time	1989 -	Yes
	Princess Alexandra Hospital	Tutor	Part time	1989 -	Yes
	Royal Australasian College of Physicians	Supervisor of Advanced Trainees	Full time	1989 -	Yes

(CIE) Stephen McDonald	Central Northern Adelaide Renal and Transplantation Service (SA Health)	Head, dialysis	Part time	June 2014 -	Yes
	Country Health SA LHN	Clinical Director, Renal Services	Part time	October 2009 -	Yes
	Central Northern Adelaide Renal and Transplantation Service (SA Department of Health)	Senior Staff Nephrologist	Full time	January 2005 -	Yes

CV-A: Appointments (last 5 years only)

Team Member	Type	Organisation	Title	Role	Period	Current
(CIA) Jonathan Craig	Appointment	Cochrane Collaboration	Funding Arbitration Panel	Member	2014 -	Yes
	Appointment	Cancer Council NSW	Advisory Committee for Internal Research	Committee Member	2014 -	Yes
	Appointment	NSW Health	Health and Medical Research Governance Project Expert Group	Member	2013 - 2014	No
	Appointment	Pediatric Trials Network Australia	Steering Committee member	Contribute to the direction ,focus and expansion of the trial network	2012 -	Yes
	Appointment	NSW Better Treatments for Children Research Network	Chair	xxxxxx	2012 -	Yes
	Appointment	Kidney Health Australia	Director	Contribute to the strategic direction of research, education and policies around kidney health	December 2011 -	Yes
	Appointment	KDIGO (International Guidelines on Chronic Kidney Disease)	Executive	Member	2010 - 2013	No
	Appointment	Department of Health and Ageing	Protocol Advisory Sub-Committee of Medical Services Advisory Committee	Member	2010 -	Yes
	Appointment	World Health Organisation	Advisory Group on Clinical Trials in Children	Member	2009 - 2012	No
	Appointment	Cochrane Collaboration	Co-Chair	N/A	2009 - 2013	No
	Appointment	Australasian Kidney Trials Network	Scientific Committee	Members	2008 -	Yes
	Appointment	Cochrane Collaboration	Steering Committee	Member	2007 - 2013	No

	Appointment	Department of Health and Ageing	Economics Sub-Committee of the Pharmaceutical Benefits Advisory Committee	Member	2001 - 2011	No
	Appointment	Cochrane Renal Group, Cochrane Collaboration	Coordinating Editor		2000 -	Yes
(CIB) David Johnson	Appointment	International Society of Nephrology	Councillor	Councillor	2013 - 2019	Yes
	Appointment	International Society of Nephrology	Councillor	Set directions, supervise the activities of the ISN Committees and establish policies and procedures that outline how ISN will operate and the leadership to accomplish its mission	May 2013 - June 2019	No
	Appointment	International Society of Nephrology Global Outreach (GO) Committee	Member	Member	2013 -	Yes
	Appointment	International Society of Peritoneal Dialysis Peritoneal Dialysis Outcomes and Practice Patterns Study Steering Committee(PDOPPS)	Co-Chair	Co-Chair	2012 -	Yes
	Honorary Appointment	ISPD Guideline Group on "Cardiovascular and Metabolic Recommendations in Peritoneal Dialysis	Member	Member	2012 -	Yes
	Appointment	ISPD PDOPPS Infection Management and Prevention Workgroup	Chair	Chair	2012 -	Yes
	Appointment	International Society of Peritoneal Dialysis Guidelines for Managing Cardiovascular Risk	Member	Guideline writer	2012 -	Yes
	Honorary Appointment	NHMRC Research Translation Faculty	Member	Member	2012 -	Yes

	Appointment	National Renal-Specific Disease Registry Steering Committee	Member		2012 -	Yes
	Appointment	Australian Indigenous Clinical InfoNet	Content Reviewer	Content Reviewer	2012 -	Yes
	Appointment	Caucus, Translational Research Institute	Executive Member		2012 -	Yes
	Appointment	National Blood Authority Iron Supplementation Advisory Group	Member		2012 -	Yes
	Appointment	NHMRC Academy	Member		2011 -	Yes
	Appointment	International Home Dialysis Symposium	Deputy Chair		2010 -	No
	Appointment	Australian Primary Care Collaborative in Indigenous Healthcare	Member	Bridging the Gap Expert Reference Panel	2010 -	Yes
	Appointment	Shire	Member	Consultant Advisory Board	2010 - 2012	No
	Appointment	International Society of Peritoneal Dialysis	Councillor	Set directions, supervise the activities of the ISPD Committees and establish policies and procedures that outline how ISPD will operate and the leadership to accomplish its mission	September 2009 - September 2017	No
	Appointment	Singapore National Medical Research Grants	Grant Reviewer		2009 - 2010	No
	Appointment	CARI Guidelines Group for the Management of Early Chronic Kidney Disease in Primary Healthcare.	Convener		2009 -	Yes
	Appointment	International Society of Peritoneal Dialysis Guideline Group on "Peritonitis Treatment"	Member		2009 -	Yes

	Appointment	National Vascular Disease Prevention Alliance	Member		2009 -	Yes
	Appointment	International Society of Peritoneal Dialysis Guideline Group on "Access Care"	Member		2009 -	Yes
	Appointment	International Society of Peritoneal Dialysis Guideline Group on "Peritonitis Prevention"	Member		2009 -	Yes
	Appointment	International Society of Peritoneal Dialysis Clinical Research Taskforce	Co-chair		2009 -	Yes
	Appointment	National Absolute Cardiovascular Risk Guidelines	Member		2009 -	Yes
	Appointment	Baxter Extramural Grants Program	Reviewer	Reviewed and scored research applications	2009 - 2012	No
	Appointment	Journal of Infection	Reviewer		2009 -	Yes
	Appointment	Open Drug Discovery Journal	Reviewer		2009 -	Yes
	Appointment	Australian Iron-Deficiency Guidelines Steering Committee	Member		2009 -	Yes
	Appointment	Australasian Proteinuria Consensus Working Party	Co-chair		2009 -	Yes
	Appointment	Australian Primary Care Collaborative in Chronic Disease Prevention and Self-Management Expert Reference Panel	Member		2009 -	Yes
	Appointment	Kidney Health Australia and Australian and New Zealand Society of Nephrology Committee for the National Chronic Kidney Disease Summit 2009	Member		2009 - 2009	No

	Appointment	National Clinical Guidelines of Cardiovascular Disease Risk	Member		2009 -	Yes
	Appointment	Queensland Clinical Senate	Executive Member		2009 -	Yes
	Appointment	Queensland Health	Statewide Clinical Networks Council		2008 - 2009	No
	Appointment	Queensland Statewide Renal Clinical Network	Chair	Chair	2008 - 2013	Yes
	Appointment	Renal Replacement Therapy Steering Committee	Member		2008 -	Yes
	Appointment	Queensland Health Private-Public Partnerships Steering Committee	Member	Privided advice regarding PPP	2008 -	Yes
	Appointment	Queensland End of Life Care Steering Committee	Member		2008 -	Yes
	Appointment	Clinical Services Capability Framework Executive Steering Committee	Member		2008 -	Yes
	Appointment	PAH Emergency Department, Helipad & Radiation Oncology Project Steering Committee	Member		2008 - 2009	No
	Appointment	Queensland Clinical Services Capability Framework Executive Steering Committee	Member		2008 -	Yes
	Appointment	Clinician Performance Support Service	Peer Assessor		2008 -	Yes
	Appointment	Statewide Clinical Networks Council	Member		2008 - 2009	Yes
	Appointment	International Society of Peritoneal Dialysis Council	Member		2008 -	Yes
	Appointment	Sandoz Binocrit Advisory Board	Member	Provided advice re ESA therapy	2008 - 2010	No
	Appointment	Pfizer Binocrit Consultant Advisory Board	Member	Provided advice re ESA therapy	2008 - 2010	No

	Appointment	Gambro Peritoneal Dialysis Advisory Board	Member	Provided advice re peritoneal dialysis	2008 - 2010	No
	Appointment	Genzyme Chronic Kidney Disease Advisory Board	Chair	Provided advice in relation to kidney disease and phosphate management	2008 - 2010	No
	Appointment	Queensland Statewide Renal Clinical Network	Qld Clinical Senator	Qld Clinical Senator	2008 - 2013	Yes
	Appointment	Australian Primary Care Collaborative in Diabetes Mellitus Expert Reference Panel	Member		2007 -	Yes
	Appointment	Australian Pimary Care Collaborative in Coronary Heart Disease Expert Reference Panel	Member		2007 -	Yes
	Appointment	International Society of Peritoneal Dialysis ISPD Guideline Group on "Time on PD - when to transfer"	Member		2007 -	Yes
	Appointment	International Society of Peritoneal Dialysis Standards and Guidelines Committee	Member	on "Time on PD - when to transfer"	2007 -	Yes
	Appointment	Astra Zeneca Advisory Board	Member	Provided advice in relation to kidney disease and lipid management	2007 - 2010	No
	Appointment	Roche CERA Advisory Board	Member		2007 -	Yes
	Appointment	Statewide Aminoglycoside Dosing Guidelines Working Party	Member		2007 -	Yes
	Appointment	University of Queensland	Professor of Population Health		2007 -	Yes

	Appointment	National Chronic Kidney Disease Strategy & Policy Working Group	Member	Providing high level advice to the kidney sector and Federal government on the direction and content of programs addressing CKD early detection and prevention of progression.	2007 -	Yes
	Appointment	International Society of Peritoneal Dialysis Membership Committee	Member		2006 -	Yes
	Appointment	Princess Alexandra Hospital Clinical Council	Executive Member		2006 - 2009	No
	Appointment	Queensland Health	Chair of Division of Medicine, Princess Alexandra Hospital		2006 - 2009	No
	Appointment	Princess Alexandra Hospital, Brisbane, Qld	Chair of Medicine	Administrative head to 19 departments in the Division of Medicine at Princess Alexandra Hospital	2006 - 2009	No
	Appointment	ANZSN CARI Guidelines for Infection Control in Dialysis Units Working Party	Member		2006 -	Yes
	Appointment	Asia-Pacific Dialysis Consultant Advisory Board (Baxter Healthcare)	Australian Representative		2005 -	Yes
	Appointment	National Chronic Kidney Disease Strategy Early Kidney Disease Working Group	Chair		2004 -	Yes
	Appointment	Council of Healthcare Advisors (Gerson Lehrman)	Member		2004 -	Yes
	Appointment	ANZSN CARI Guidelines for Evaluation of Renal Function	Convenor		2004 -	Yes
	Appointment	Operations Secretariat, Australasian Clinical Trials Network in Nephrology	Deputy Chair		2004 -	Yes

	Appointment	Australasian Kidney Trials Network	Founding Member		2004 -	Yes
	Appointment	Australasian Creatinine and eGFR Consensus Working Party	Co-chair	convened by Kidney Health Australia, Australian and New Zealand Society of Nephrology, Australasian Association of Clinical Biochemists and Royal College of Pathologists of Australasia	2004 -	Yes
	Appointment	Baxter Dialysis Unit Consultant Review Board	Member		2004 -	Yes
	Appointment	Australasian Kidney Trials Network	Deputy Chair of Operations Secretariat		2004 -	Yes
	Appointment	University of Queensland	Professor of Medicine		2004 -	Yes
	Appointment	Kidney Check Australia Taskforce	Chair		2004 -	Yes
	Appointment	Pure Red Cell Aplasia Global Scientific Advisory Board	Australian Representative		2003 -	Yes
	Appointment	CARI Guidelines for Peritoneal Dialysis Adequacy	Convenor		2003 -	Yes
	Appointment	Baxter Healthcare, Global Dialysis Advisory Board	Member	Provided advice re peritoneal dialysis	2003 - 2012	No
	Appointment	Amgen Aranesp Advisory Board	Member	Provided advice re ESA therapy	2002 - 2012	No
	Appointment	Princess Alexandra Hospital, Brisbane, Qld	Director of Renal Medicine,		2002 -	Yes
	Appointment	Qld Royal Australasian College of Physicians College Lecture Series (Renal)	Convenor		2001 -	Yes
	Appointment	Kidney Check Australia Taskforce	Member		2001 -	Yes

	Appointment	Australian and New Zealand Clinical Practice Guidelines on Icodextrin Usage in Peritoneal Dialysis	Working Party Member		2001 -	Yes
	Appointment	Australian Cancer Network National Clinical Practice Guidelines for Post-Transplant Lymphoproliferative Disorder and Other Immunodeficiency-Related Lymphomas	Working Party Member		2001 -	Yes
	Appointment	ANZDATA Registry	Peritoneal Dialysis Project Manager		2001 - 2009	No
	Appointment	National Early Renal Impairment Task Force	Australian and New Zealand Society of Nephrology Representative		2001 -	Yes
	Appointment	ANZDATA Registry Peritoneal Dialysis Advisory Committee	Member		2000 -	Yes
	Appointment	Australian and New Zealand Dialysis and Transplant Association (ANZDATA) Registry Management Committee	Member		2000 -	Yes
	Appointment	13th Asian Colloquium in Nephrology	International Faculty Member		2000 -	No
	Appointment	ANZSN CARI Guidelines for Prevention of Renal Failure Progression	Working Party Member		2000 -	Yes
	Appointment	Diabetes Australia	Research Grants Reviewer		1999 -	Yes
	Appointment	ANZSN CARI Guidelines for Peritoneal Dialysis Adequacy	Working Party Member		1999 -	Yes
	Appointment	Janssen-Cilag Consultant Advisory Board	Member		1999 -	Yes

	Appointment	Weekly Renal Educational Meetings and Renal Journal Club, Princess Alexandra Hospital	Convenor		1998 -	Yes
	Appointment	National Health and Medical Research Council	Reviewer of Research Grant Applications		1998 -	Yes
	Appointment	Princess Alexandra Hospital Renal Research Laboratory	Head of Renal Research Laboratory		1998 -	Yes
	Appointment	Australian Kidney Foundation / Kidney Health Australia	Reviewer of Research Grant Applications		1998 -	Yes
(CIC) Jeremy Chapman	Appointment	Western Sydney Local Health District Board	Member - Western Sydney Local Health District Board	Board Member	2011 - 2016	Yes
	Appointment	Clinical Advisory Committee, AOTD	Chair - Clinical Advisory Committee, AOTD		April 2010 - April 2010	No
	Appointment	Transplant Australia	Medical Director - Transplant Australia		April 2010 - April 2010	No
	Appointment	The Transplantation Society	Past President	Past President	2010 - 2012	Yes
	Appointment	DonateLife Network Forum Organising Committee	Chair - DonateLife Network Forum Organising Committee	chair	2010 - 2011	Yes
	Appointment	The World Health Organisation Expert Advisory Panel on Human Cell Tissue and Organ Transplantation	Advisory Member - The World Health Organisation Expert Advisory Panel on Human Cell Tissue and Organ Transplantation		2010 - 2014	Yes
	Appointment	DonateLife Network Forum Organising Committee	Committee Member - DonateLife Network Forum Organising Committee	Committee member	2010 - 2011	No
	Appointment	Declaration of Istanbul Custodian Group	Chair - Declaration of Istanbul Custodian Group	Chair of custodian group	2009 -	Yes
	Appointment	Declaration of Istanbul Custodian Group	Chair - Declaration of Istanbul Custodian Group	chair	2009 - 2014	Yes
	Appointment	Advisory Council National Organ & Tissue Authority	Member - Advisory Council National Organ & Tissue Authority	Advisory	2009 - 2016	No

	Appointment	The Transplantation Society	President - The Transplantation Society		2008 - 2010	No
	Appointment	General World Marrow Donor Assoc	Secretary-General World Marrow Donor Assoc		2008 - 2010	No
	Appointment	General World Marrow Donor Association	Secretary	Secretary of association	2008 - 2010	No
	Appointment	Therapeutic Goods Committee, Subcommittee on Biologicals	Member - Therapeutic Goods Committee, Subcommittee on Biologicals		2008 -	Yes
	Appointment	SWAHS Area Medical Staff Executive council	Chairman - SWAHS Area Medical Staff Executive council	Chairman	2006 - 2009	No
	Appointment	SWAHS Area Medical Staff Executive Council	Chairman SWAHS Area Medical Staff Executive Council	Chair	2006 - 2009	No
	Appointment	Westmead Research Hub Council	Member - Westmead Research Hub Council		2001 -	Yes
	Appointment	Asian Society of Transplantation	Advisory Council, Asian Society of Transplantation		2000 -	Yes
	Appointment	NSW Clinical Strategy Steering Committee	Member - NSW Clinical Strategy Steering Committee		1999 -	Yes
	Appointment	NSW Sterile Fluids Contract Committee	Member - NSW Sterile Fluids Contract Committee		1996 -	Yes
	Appointment	Australasian Donor Awareness Program	Chairman - Australasian Donor Awareness Program		1995 -	Yes
	Appointment	Westmead Hospital	FRACP course		1991 -	No
	Appointment	Westmead	General Clinical Training Committee		1991 -	No
	Appointment	NSW Transplant Advisory Committee	Member - NSW Transplant Advisory Committee		1991 -	Yes
	Appointment	National Pancreas Transplant Registry	Manager - National Pancreas Transplant Registry		1990 -	Yes
	Appointment	ANZ Organ Donor Registry Committee	Member - ANZ Organ Donor Registry Committee		1989 -	Yes
	Appointment	Renal Transplant Advisory Committee	Member - Renal Transplant Advisory Committee		1988 -	Yes
	Appointment	National Organ Matching Service	Manager, National Organ Matching Service		1988 -	Yes

	Appointment	University of Sydney	Final Year tutor/Year 4 tutor		1987 -	No
	Appointment	Cambridge, Manchester and Oxford Universities	Elective Student Supervisor		1987 -	No
	Appointment	University of Sydney	GMP lecturer & Tutor		1987 -	No
	Appointment	University of Sydney	Year 3 introductory lecturer		1987 -	No
	Appointment	University of Sydney	Year 4 Renal Medicine lecturer		1987 -	No
(CID) Carmel Hawley	Appointment	Australian Clinical Trials Alliance (ACTA)	Founding Director	Founding Director	2013 -	Yes
	Appointment	Dialysis Nephrology Transplant Subcommittee	Committee member	Committee member	2012 -	Yes
	Appointment	Canadian Trials network	Advisory Board member	Advisory Board member	2010 -	Yes
	Appointment	International Quotidian Registry	Scientific Committee Member	Scientific Committee Member	2009 -	Yes
	Appointment	Queensland Health	Service Level Mapping Review Committee Member	Service Level Mapping Review Committee Member	2008 - 2009	No
	Appointment	Home Dialysis Advisory Committee	Inaugural Chair	Inaugural Chair	2007 - 2012	No
	Appointment	KDIGO (international society of nephrology kidney disease guidelines)	Inaugural member	Inaugural member	2005 -	No
	Appointment	The University of Queensland, School of Population Health	Associate Professor	N/A	2005 - 2009	No
	Appointment	Australasian Kidney Trials Network, The University of Queensland	Inaugural chair, Operations Secretariat	Inaugural chair, Operations Secretariat Australasian Kidney Trials Network	2005 -	Yes
	Appointment	Kidney Health Australia	Board Member	Board Member, Kidney Health Australia	2004 - 2009	No
	Appointment	The University of Queensland, School of Medicine	Associate Professor	N/A	2004 -	Yes

	Appointment	Australians with Renal Impairment (CARI)	Co-Chair of KHA-CARI working group for guidelines on biochemical and haematological targets	Co-Chair of for Australians with Renal Impairment (CARI) working group for guidelines on biochemical and haematological targets	2002 -	Yes
	Appointment	Caring for Australians with Renal Impairment (KHA-CARI)	Member of the KHA-CARI working group for guidelines on bone disease	Member of the Caring for Australians with Renal Impairment (CARI) working group for guidelines on bone disease in the pre-dialysis patient	1999 -	Yes
	Appointment	Princess Alexandra Hospital, Brisbane, Australia	Director of Haemodialysis Services, Princess Alexandra Hospital	Director of Haemodialysis Services, Princess Alexandra Hospital	1997 -	Yes
(CIE) Stephen McDonald	Honorary Appointment	SA Health Clinical Senate	Member	Attend meetings and provide advice on relevant matters	October 2014 -	Yes
	Appointment	The University of Adelaide	Clinical Professor	Research and teaching	January 2014 -	Yes
	Honorary Appointment	Australian Organ and Tissue Donation and Transplantation Authority	Electronic Donor Record Oversight Committee	Member	June 2012 - March 2014	No
	Honorary Appointment	Royal Australasian College of Physicians	Member, Senior Examination Panel	involvement in RACP clinical examination as national moderating panel member.	January 2011 -	Yes
	Honorary Appointment	Australian Commission on Safety and Quality in Healthcare	Member, Clinical Quality Registries Advisory Committee	Committee member -- tasked with providing Commission with advice abot implementation of their agenda regarding clinical quality registries	June 2011 - December 2012	No
	Honorary Appointment	National Organ and Tissue Donation and Transplantation Authority	Member, National Clinial Information System Committee	Provide advice on clinial information systems for Authority in field of organ donation and transplantation	June 2009 - December 2010	No

	Honorary Appointment	Australia Institute of Health and Welfare	Member, CKD Data Monitoring Centre Medical Advisory Committee	Provide expert advice to the AIHW DMC for CKD	January 2008 - June 2013	No
	Honorary Appointment	University of Adelaide	Clinical Associate Professor	Reseach & teaching	November 2008 - December 2013	No
	Honorary Appointment	Australasian Kidney Trials Network	Member, Scientific Advisory Committee		January 2007 -	No
	Honorary Appointment	SA Renal Clinical Network	Member, Steering Committee		July 2007 -	No
	Honorary Appointment	SA Renal Clinical Network	Member of Steering Committee; Chair of Country Renal Services Working Group	Develop and implement policy and strategy for county renal services in SA	May 2007 -	Yes
	Honorary Appointment	Australia & New Zealand Dialysis & Transplant Registry	Executive Officer	Reponsible for oversight of all operations of this major outcomes Registry	January 2005 -	Yes
	Honorary Appointment	Royal Australiasian College of Physicians	Member, National Examination Panel	Be expert examiner and moderator of RACP clinical examination	June 2004 - June 2010	No

CV-PM: Professional Memberships

Team Member	Organisation	Duration	Current
(CIA) Jonathan Craig	Academy of Surgical Educators	2013 -	Yes
	The Transplantation Society	2007 -	Yes
	Italian Society of Nephrology (Honorary)	2005 -	Yes
	American Society of Nephrology	2004 -	Yes
	European Dialysis and Transplant Association-European Renal Association	2004 -	Yes
	Australasian Epidemiological Association	1999 -	Yes
	Australia and New Zealand Paediatric Nephrology Association	1998 -	Yes
	The International Society of Nephrology	1995 -	Yes
	The International Paediatric Nephrology Association	1995 -	Yes
	Australia and New Zealand Society of Nephrology	1994 -	Yes
	The Royal Australasian College of Physicians	1994 -	Yes
	Paediatric Research Society of Australia	1994 -	Yes

(CIB) David Johnson	American Journal of Kidney Disease - Editorial Board Member	2014 - 2019	No
	Australian Primary Care Collaborative in Chronic Kidney Disease Expert Reference Panel (run by Improvement Foundation)	2013 -	Yes
	American Society of Nephrology (Fellow)	2013 -	Yes
	European Renal Association	2008 -	Yes
	Together Union	2007 -	Yes
	Royal Australasian College of Physicians	1994 -	Yes
	Australian Medical Association	1988 -	Yes
	International Society of Nephrology-Councillor	2013 - 2019	Yes
	Royal North Shore Hospital, St. Leonards, Sydney, NSW.	1995 - 1997	No
(CIC) Jeremy Chapman	Asian Pacific Society of Nephrology	2000 -	Yes
	Asian Transplantation Society	1997 -	Yes
	The Transplantation Society	1993 -	Yes
	Transplantation Society of Australia and New Zealand	1993 -	Yes

(CID) Carmel Hawley	International Society for Hemodialysis	2008 -	Yes
	Society for Clinical Trials Inc.	2006 -	Yes
	Australian Medical Association	2001 -	Yes
	International Society of Nephrology	2000 -	Yes
	American Society of Nephrology	1995 -	Yes
	Transplantation Society of Australia and New Zealand	1993 -	Yes
	Australian Salaried Medical Officers Federation	1989 -	Yes
	Australian and New Zealand Society of Nephrology	1989 -	Yes
	Royal Australasian College of Physicians	1988 -	Yes
(CIE) Stephen McDonald	Australasian Association of Consultant Physicians	2013 -	Yes
	International Society of Peritoneal Dialysis	2012 -	Yes
	Transplantation Society of Australia and New Zealand	2007 -	Yes
	Australasian Epidemiological Association	2007 -	Yes

	International society of nephrology	2005 -	Yes
	Royal Australasian College of Physicians	1997 -	Yes
	Australian Medical Association	1991 -	Yes

CV-TPP: Translation into Policy/Practice (last 5 years only)

(CIA) Jonathan Craig, **Type:** Policy, **Year of Research Results:** 2014
Funding Source: NHMRC: Yes, Other Australian Source: No, International Source: No
Research: Best-practice approach to the conduct and reporting of qualitative studies was used to formulate editorial policy for these manuscript types (P001836818 and P001835467)
Organisations Affected: National Kidney Foundation/Authors, editors, and referees of the American Journal of Kidney Disease, **Year of Change:** 2014
Changes: Introduction of an editorial policy for all qualitative studies
Outcomes: Improved conduct and reporting of qualitative studies

(CIA) Jonathan Craig, **Type:** Policy, **Year of Research Results:** 2010
Funding Source: NHMRC: Yes, Other Australian Source: No, International Source: No
Research: A series of studies about the validity of monitoring response to a variety of drugs (P9973038, P9973039, P9973040, P9973037,P9973042) resulted in a policy relevant approach to the use of response rules by regulators (P9973043)
Organisations Affected: Department of Health and Ageing (Pharmaceutical benefits branch), **Year of Change:** 2013
Changes: Application of the framework for Australian health policy regarding continuation rules for high cost biologicals
Outcomes: Appropriate use and cessation of high cost biologicals for people with a diverse range of conditions (lower cost, more effective use)

(CIA) Jonathan Craig, **Type:** Policy, **Year of Research Results:** 2013
Funding Source: NHMRC: Yes, Other Australian Source: No, International Source: No
Research: A large observational, prospective study of over 15,000 children (FEVER), published in the BMJ (P1001451894) demonstrated that the 'traffic light' system, implemented by NICE for the differentiation of serious bacterial infection in young children, was only moderately accurate but could be improved by the addition of a urinalysis (to detect UTI)
Organisations Affected: UK National Institute for Health and Care Excellence (NICE), clinicians worldwide caring for children with febrile illnesses, **Year of Change:** 2013
Changes: The NICE guidance incorporated urinalysis as we recommended
Outcomes: Improved diagnosis and care for children with febrile illnesses (around 500,000 in Australia alone

(CIA) Jonathan Craig, **Type:** Policy, **Year of Research Results:** 2012
Funding Source: NHMRC: Yes, Other Australian Source: Yes, International Source: No
Research: We developed and evaluated a novel method for involving patients and their caregivers, effectively, in the development of clinical practice guidelines (P10011441030). This approach, which is based upon a parallel process for consumers, resulted in substantial changes to the original (an expansion in scope), and provided the template for future guidelines
Organisations Affected: KHA-CARI and all other guideline groups (CKD and non CKD), **Year of Change:** 2012
Changes: Effective participation by consumers in guidelines
Outcomes: Improved relevance for all stakeholders of guidelines

(CIA) Jonathan Craig, **Type:** Policy, **Year of Research Results:** 2011
Funding Source: NHMRC: Yes, Other Australian Source: No, International Source: No
Research: This systematic review of prognostic studies demonstrated that the accepted surrogates for bone and mineral disorders in CKD were not associated with clinically important outcomes, contrary to the established view. Published in JAMA (P001072941), this study also demonstrated an methodological approach to the systematic review of prognostic studies which has been widely replicated
Organisations Affected: CKD guideline groups, clinicians and patients with CKD, **Year of Change:** 2012
Changes: Loosening up of bone and mineral disease targets
Outcomes: Less frequent and aggressive management of biochemical targets for people with CKD

(CIA) Jonathan Craig, **Type:** Policy, **Year of Research Results:** 2010
Funding Source: NHMRC: Yes, Other Australian Source: Yes, International Source: No
Research: Our mixed methods research was undertaken to evaluate the impact of CARI guidelines. These studies showed (P9973167 and P9973165) demonstrated the high regard that the majority of clinicians viewed CARI but some practical and local issues impaired local uptake. This research was done at a time when there was increasing concern about the role of Industry in guideline development.
Organisations Affected: ANZSN,, **Year of Change:** 2011
Changes: Development of a series of implementation projects for CARI guidelines
Outcomes: Realignment of practice with guidelines and evidence, resulting in improved care

(CIA) Jonathan Craig, **Type:** Policy, **Year of Research Results:** 2007
Funding Source: NHMRC: Yes, Other Australian Source: No, International Source: No
Research: Our synthesis and critical evaluation of approaches to the conduct and reporting of qualitative studies resulted in the publication of COREQ, a standardised checklist for the reporting of this study type
Organisations Affected: EQUATOR, many journals (editors, authors, referees), **Year of Change:** 2009
Changes: COREQ has become the standard approach for the reporting of qualitative studies. As of May 2014 it has been used > 500 times.
Outcomes: Improved conduct and reporting of qualitative studies

(CIA) Jonathan Craig, **Type:** Policy, **Year of Research Results:** 2008
Funding Source: NHMRC: Yes, Other Australian Source: No, International Source: No
Research: Empiric and methodological studies of diagnostic test evaluation led to the formulation of an approach to conceptualising the evaluation of new test and how it should be evaluated (BMJ P9973047), and how published test results should be evaluated an incorporated into clinical practice guidelines (P9973231).
Organisations Affected: NHMRC, HTA organisations, diagnostic test researchers, guideline organisations, **Year of Change:** 2009
Changes: Improvements in how diagnostic tests are evaluated and reported, and a methodological approach used by guideline developers for the formulation of recommendations based upon test accuracy data

Outcomes:

(CIA) Jonathan Craig, **Type:** Policy, **Year of Research Results:** 1996
Funding Source: NHMRC: Yes, Other Australian Source: No, International Source: No
Research: I conducted a case-control study of circumcision demonstrating this reduces UTI in boys beyond the infant age range, contrary to prevailing beliefs (P9973097). This was followed up with a systematic review (P9973232) which confirmed these findings.
Organisations Affected: AAP, RACP. Clinicians caring for children and children themselves, **Year of Change:** 2009
Changes: The RACP statements on circumcision reflected the findings of our study, and did not recommend routine use, but limited to boys with recurrent symptomatic infections
Outcomes: More appropriate, targeted approach to circumcision leading to a reduction in unnecessary harms (infection and bleeding), and fewer UTI

(CIA) Jonathan Craig, **Type:** Practice, **Year of Research Results:** 2010
Funding Source: NHMRC: Yes, Other Australian Source: Yes, International Source: No
Research: SEARCH, a large-scale community-based cohort study of 1700 urban Aboriginal children and their caregivers involving five Aboriginal Community Controlled Healthcare Organisations, demonstrated high rates of middle ear disease and speech and language delay (P9973316).
Organisations Affected: Aboriginal Community Controlled Healthcare Organisations, urban Aboriginal children, **Year of Change:** 2014
Changes: Consistent with a policy of ‘no research without service’ for Aboriginal people, we were able to leverage an additional $1.8M from the State and Federal Governments to provide ENT operations to 150 children and speech and language therapy to 450 children (8000 individual sessions) who would not received such timely care (and delivered very efficiently over two 3-month periods). This efficient and effective model of research-policy-practice we are now formally evaluating (including an economic evaluation), as a possible model for scale-able interventions across this entire health sector.
Outcomes: Better hearing and language for 450 Aboriginal children

(CIA) Jonathan Craig, **Type:** Practice, **Year of Research Results:** 2010
Funding Source: NHMRC: Yes, Other Australian Source: Yes, International Source: No
Research: We conducted the only large-scale randomised trial to evaluate the effects of early versus later start dialysis. This study, published in NEJM (P9973065) demonstrated no improved outcomes with earlier start
Organisations Affected: Nephrologists and patients with stage V CKD worldwide, **Year of Change:** 2011
Changes: Progressive change in practice so that people are commenced upon dialysis according to symptoms and biochemical safety rather than based upon a biochemical threshold for GFR
Outcomes: Delayed commencement of dialysis resulting in fewer complications and substantial cost savings

(CIA) Jonathan Craig, **Type:** Practice, **Year of Research Results:** 2009
Funding Source: NHMRC: Yes, Other Australian Source: Yes, International Source: No
Research: We completed the largest ever randomised, placebo-controlled trial of prophylactic antibiotics in children following urinary tract infection in the world. The trial (PRIVENT), published in the NEJM (P000888383) demonstrated that cotrimoxazole reduced recurrent symptomatic infection by 6% (in absolute terms) across all sub-groups of children, and without serious adverse events.
Organisations Affected: American Academy of Pediatrics, RACP (Paediatrics), UK NICE. Paediatric clinicians world-wide, **Year of Change:** 2010
Changes: Antibiotics are selectively prescribed for young children at high risk of recurrence, or at high risk of a serious recurrence requiring hospitalisation, and regardless of underlying renal tract malformation
Outcomes: More selective and appropriate use of prophylactic antibiotics in children after a first UTI. Reduction in invasive imaging following UTI

(CIB) David Johnson, **Type:** Policy, **Year of Research Results:** 2013
Funding Source: NHMRC: No, Other Australian Source: Yes, International Source: Yes
Research: Johnson DW, Atai E, Chan M, Phoon RK, Scott C, Toussaint ND, Turner G, Usherwood T, Wiggins KJ. KHA-CARI Guideline: Early chronic kidney disease: detection, primary prevention and management. Nephrology 18:340-350, 2013.
Organisations Affected: Caring for Australasians with Renal Insufficiency (KHA-CARI)
Kidney Check Australia Taskforce
Royal Australasian College of General Practitioners
Australian and New Zealand Society of Nephrology, **Year of Change:** 2013
Changes: These Guidelines make recommendations for the screening, detection, prevention and management of early chronic kidney disease and were primarily directed towards primary healthcare (general practitioners, indigenous healthcare workers, practice nurses, community pharmacists). They were the first KHA-CARI Guidelines to be informed by patient focus groups.
Outcomes: The results have been incorporated into the Royal Australasian College of General Practitioners "Green Book" (guidelines for prevention), the Kidney Check Australia Taskforce Guidelines for Management of Chronic Kidney Disease in General Practitioners and Kidney Health Australia Patient Information Brochures about CKD.

(CIB) David Johnson, **Type:** Policy, **Year of Research Results:** 2012
Funding Source: NHMRC: No, Other Australian Source: Yes, International Source: No
Research: Johnson DW, Hayes B, Gray N, Hawley CM, Hole J, Mantha M. Renal services disaster planning: Lessons learnt from the 2011 Queensland floods and North Queensland cyclone experiences. Nephrology (In press; Acceptance date 15 October 2012).
Organisations Affected: Queensland Health, **Year of Change:** 2012
Changes: This evaluation of the renal services disaster response to the 2011 Queensland disasters (Queensland floods and Cyclone Yasi crisis) led to the development of the Queensland Health Statewide Renal Services Disaster Plan (http://qheps.health.qld.gov.au/srcn/html/disaster.htm)
Outcomes: The Statewide Renal Services Disaster Plan was recently enacted for the 2013 Cyclone Oswald disaster and performed exceptionally well.

(CIB) David Johnson, **Type:** Policy, **Year of Research Results:** 2010
Funding Source: NHMRC: No, Other Australian Source: Yes, International Source: No
Research: Fahim M, Hawley CM, McDonald SP, Brown FG, Rosman JB, Wiggins KJ, Bannister K, Johnson DW. Culture-negative peritonitis in peritoneal dialysis patients in Australia: predictors, treatment and outcomes in 435 cases. Am J Kidney Dis 2010; 55:690–7. Findings of the study were incorporated into the ISPD GUIDELINES/RECOMMENDATIONS: PERITONEAL DIALYSIS-RELATED INFECTIONS RECOMMENDATIONS: 2010 UPDATE
Organisations Affected: International Society of Peritoneal Dialysis and International Dialysis Units, **Year of Change:** 2010
Changes: This study was the largest ever report on the associations, and outcomes of culture negative peritoneal dialysis peritonitis and demonstrated that this entity had favourable outcomes and informed the ISPD guidelines on this favourable outcome and the importance of assessing culture technique if culture negative peritonitis rates were high.
Outcomes:

(CIB) David Johnson, **Type:** Policy, **Year of Research Results:** 2009
Funding Source: NHMRC: No, Other Australian Source: Yes, International Source: No
Research: Barraclough K, Hawley CM, McDonald SP, Brown FG, Rosman JB, Wiggins KJ, bannister K, Johnson DW. Corynebacterium peritonitis in Australian peritoneal dialysis patients: predictors, treatment and outcomes in 82 cases. Nephrol Dial Transplant 2009; 24:3834–9.
Organisations Affected: International Society of Peritoneal Dialysis, **Year of Change:** 2010
Changes: This study was the largest ever report on the associations, and outcomes of corynebactium related peritoneal dialysis peritonitis and demonstrated that this entity had generally favourable outcomes and informed the ISPD guidelines on this favourable outcome and the importance of adequate antibiotic therapy.
Outcomes: Findings of the study were incorporated into the ISPD GUIDELINES/RECOMMENDATIONS: PERITONEAL DIALYSIS-RELATED INFECTIONS RECOMMENDATIONS: 2010 UPDATE

(CIB) David Johnson, **Type:** Policy, **Year of Research Results:** 2010
Funding Source: NHMRC: No, Other Australian Source: Yes, International Source: No
Research: Govindarajulu S, Hawley CM, McDonald SP, Brown FG, Rosman JB, Wiggins KJ, Bannister K, Johnson DW. Staphylococcus aureus peritonitis in Australian peritoneal dialysis patients: predictors, treatment and outcomes in 503 cases. Perit Dial Int 2010; 30:311–19. Findings of the study were incorporated into the ISPD GUIDELINES/RECOMMENDATIONS: PERITONEAL DIALYSIS-RELATED INFECTIONS RECOMMENDATIONS: 2010 UPDATE
Organisations Affected: International Society of Peritoneal Dialysis, **Year of Change:** 2010
Changes: This study was the largest ever report on the associations, and outcomes of Staphylococcus aureus related peritoneal dialysis peritonitis and informed the ISPD guidelines about poorer outcomes with MRSA vs. MSSA and that the use of Vancomycin vs. Cephazolin in the initial regimen was not predictive of outome.
Outcomes:

(CIB) David Johnson, **Type:** Policy, **Year of Research Results:** 2006
Funding Source: NHMRC: No, Other Australian Source: Yes, International Source: No
Research: Johnson DW, Wong J, Wiggins KJ, Kirwan R, Griffin A, Preston J, Wall D, Campbell SB, Isbel NM, Mudge DW, Hawley CM, Nicol DL. A randomized, controlled trial of coiled versus straight swan neck Tenckhoff catheters in peritoneal dialysis patients. American Journal of Kidney Disease 48(5):812-21, 2006.
Organisations Affected: International Society of Peritoneal Dialysis. Perit0oneal dialysis units around the world., **Year of Change:** 2009
Changes: This research demonstrated that coiled Tenckhoff catheters are associated with a higher risk of technique failure compared with straight catheters.
Outcomes: The ISPD Peritoneal Dialysis Access Guidelines have recommended caution with the use of colied PD catheters. Coiled catheters have not been used at Princess Alexandra Hospital since 2006.

(CIB) David Johnson, **Type:** Policy, **Year of Research Results:** 2008
Funding Source: NHMRC: No, Other Australian Source: Yes, International Source: No
Research: Wiggins KJ, Johnson DW, Craig JC, Strippoli GFM. Treatment of peritoneal dialysis-associated peritonitis: a systematic review of randomized controlled trials (Cochrane Review). Cochrane Database of Systematic Reviews 2008, Issue 1. Art. No.: CD005284. DOI: 10.1002/14651858.CD005284.pub2.
Organisations Affected: International Society of Peritoneal Dialysis, **Year of Change:** 2009
Changes: These guidelines provided the best available evidence for treatment of peritoneal dialysis-associated peritonitis. Importantly, the study demonstrated that no agent was superior to any other, but that the intraperitoneal route was superior to the oral route.
Outcomes: The recommendations of our systematic review have now been incorporated into the ISPD Peritonitis Guidelines.

(CIB) David Johnson, **Type:** Practice, **Year of Research Results:** 2013
Funding Source: NHMRC: No, Other Australian Source: Yes, International Source: Yes
Research: Johnson DW, Badve SV, Pascoe EM, Beller E, Cass A, Clark C, de Zoysa J, Isbel NM, McTaggart S, Morrish AT, Playford EG, Scaria A, Snelling P, Vergara LA, Hawley CM. Antibacterial honey for the prevention of peritoneal-dialysis-related infections (HONEYPOT): a randomised trial. The Lancet Infectious Diseases 14(1):23-30, 2014.
Organisations Affected: International Society of Peritoneal Dialysis (ISPD)
Caring for Australasians with Renal Insufficiency (CARI)
Australian and New Zealand PD Units, **Year of Change:** 2013
Changes: This RCT of 371 PD patients found that daily topical exit-site application of antibacterial honey was not superior to nasal mupirocin prophylaxis for the prevention of PD-associated infection, may have been inferior in patients with diabetes, and resulted in local skin reactions in 6%and study withdrawal in 29%. The results of this trial, published in Lancet Infectious Diseases with an accompanying editorial, indicated that honey cannot be routinely recommended for prevention of PD-associated infections.
Outcomes: The findings have resulted in general abandonment of this infection control strategy for both PD and haemodialysis catheters in Australia and other parts of the world (particularly UK) and will be incorporated into the next round of ISPD Infection Prevention Guidelines and the KHA-CARI Guidelines.

(CIB) David Johnson, **Type:** Practice, **Year of Research Results:** 2013
Funding Source: NHMRC: No, Other Australian Source: Yes, International Source: No
Research: McMahon E, Bauer J, Hawley CM, Isbel N, Stowasser M, Johnson DW, Campbell KL. Effect of sodium restriction on ambulatory blood pressure and other cardiovascular risk factors in chronic kidney disease: results of the LowSALT CKD study, a double-blind placebo-controlled randomized-crossover trial. Journal of the American Society of Nephrology 24(12):2096-130, 2013.
Organisations Affected: Kidney Health Australia Caring for Australasians with Renal Insufficiency (KHA-CARI)
Kidney Check Australia Taskforce, **Year of Change:** 2013
Changes: This double-blind, placebo-controlled, randomized-crossover trial found that salt restriction (60-80 vs 180-200 mmol/day) resulted in statistically significant and clinically important reductions in blood pressure, extracellular fluid volume and albuminuria/proteinuria in patients with moderate-to-severe CKD. The magnitude of change was more pronounced than reported in patients without CKD, suggesting CKD patients may be particularly salt-sensitive. The trial results provided compelling evidence that sodium restriction should be emphasized in the management of patients with CKD as a means to reduce cardiovascular risk and risk for CKD progression.
Outcomes: Salt restriction (60-80 mmol/day) has been advocated in the 2013 KHA-CARI Guidelines for Early Chronic Kidney Disease and in the 2013 Management of Chhronic Kidney Disease in General Practice Guidelines issued by the Kidney Check Australia Taskforce.

(CIB) David Johnson, **Type:** Practice, **Year of Research Results:** 2013
Funding Source: NHMRC: No, Other Australian Source: Yes, International Source: Yes
Research: Palmer SC, Navaneethan SD, Craig JC, Perkovic V, Johnson DW, Nigwekar SU, Hegbrant J, Strippoli GFM. HMG CoA reductase inhibitors (statins) for kidney transplant recipients. Cochrane Database of Systematic Reviews 2014, Issue 1. Art. No.:CD005019. DOI: 10.1002/14651858.CD005019.pub4
Organisations Affected: Kidney Disease Improving Global Outcomes (KDIGO), **Year of Change:** 2013
Changes: This Cochrane systematic review and meta-analysis of 22 randomised controlled trials (3465 participants) compared the effects of statins with those of placebo, no treatment, standard care, or statins on mortality, cardiovascular events, kidney function and toxicity in kidney transplant recipients. The key findings were that statins may reduce cardiovascular events in kidney transplant recipients, although treatment effects are imprecise. Statin treatment has uncertain effects on overall mortality, stroke, kidney function, and toxicity outcomes in kidney transplant recipients.
Outcomes: Based on the findings of our systematic review, the 2013 KDIGO Guidelines on Lipid Management in Chronic Kidney Disease state "In adult kidney transplant recipients, we suggest treatment with a statin. (2B)"

(CIB) David Johnson, **Type:** Practice, **Year of Research Results:** 2012
Funding Source: NHMRC: Yes, Other Australian Source: No, International Source: No
Research: National Vascular Disease Prevention Alliance. Guidelines for the management of absolute cardiovascular disease risk. National Stroke Foundation, Melbourne, 2012.
Organisations Affected: RACGP, RACP, Kidney Health Australia, National Heart Foundation, National Stroke Foundation, Australian Diabetes Society,
Year of Change: 2012
Changes: This NHMRC-funded and endorsed guidelines by the National Vascular Disease Prevention Alliance (of which I am a member) made new and important recommendations for managing adults based on absolute cardiovascular risk

Outcomes: Endorsed by NHMRC

(CIB) David Johnson, **Type:** Practice, **Year of Research Results:** 2012
Funding Source: NHMRC: No, Other Australian Source: Yes, International Source: No
Research: Johnson DW, Jones G, Chadban S, Colagiuri S, Jerums G, Ludlow M, Macisaac R, Martin H, Mathew T, Polkinghorne K, Usherwood T. Chronic kidney disease and measurement of albuminuria/proteinuria: A Position Statement. Medical Journal of Australia 197(4): 224, 2012
Organisations Affected: Australian and New Zealand Pathology Laboratories, RCPA, AACB, RACGP, ASCEPT, Kidney Health Australia, Australian Diabetes Society, ANZSOM, ANZSN, **Year of Change:** 2012
Changes: This position statement by the Australasian Proteinuria Consensus Working Group, which I chair, made important recommendations for screening for chronic kidney disease with urine albumin:creatinine ratio measurements on a spot urine, preferably an early morning void, in individuals with at least one of 8 recongnised risk factors for kidney disease. It also made recommendations for standardisation and performance requirements of albuminuria assays, as well as reporting units and definitions of normo-, micro- and macro-albuminuria using gender-specific cut-points.
Outcomes: The recommendations have been incorporated into the CARI Guidelines and the Kidney Health Australia Management Guidelines for General Practice.

(CIB) David Johnson, **Type:** Practice, **Year of Research Results:** 2012
Funding Source: NHMRC: No, Other Australian Source: Yes, International Source: No
Research: Johnson DW, Jones G, Doogue M, Jose MD, Langham R, Lawton P, Ludlow M, Mathew T, McTaggart S, Peake M, Polkinghore K. Chronic kidney disease and automatic reporting of estimated glomerular filtration rate: New developments and revised recommendations. Medical Journal of Australia 197(4): 222-3, 2012
Organisations Affected: Australian and New Zealand Pathology Laboratories, RCPA, AACB, RACGP, ASCEPT, Kidney Health Australia, Australian Diabetes Society, ANZSOM, ANZSN, **Year of Change:** 2012
Changes: This position statement by the Australasian Creatinine and eGFR Consensus Working Group, which I chair, led to important changes, including the use of the CKD-EPI eGFR formula (instead of the MDRD formula) by all Australian and New Zealand laboratories for all adults in whom a serum creatinine measurement is requested. Important recommendations were also made in relation to acceptable creatinine assay performance, reporting limits for eGFR, creatinine measurements in children and pregnant women, use of eGFR for drug dosing, and use of CKD-EPI eGFR in indigenous racial groups.
Outcomes: The vast majority of pathology laboratories in Australia and New Zealand have enacted the Working Group's recommendations. The recommendations have also been incorporated into the CARI Guidelines and the Kidney Health Australia Management Guidelines for General Practice.

(CIB) David Johnson, **Type:** Practice, **Year of Research Results:** 2012
Funding Source: NHMRC: No, Other Australian Source: No, International Source: Yes
Research: Johnson DW, Brown FG, Clarke M, Boudville N, Elias TJ, Yin MFW, Jones B, Kulkarni H, Langham R, Ranganathan D, Schollum J, Suranyi M, Tan SH, Voss D. Biocompatible versus standard peritoneal dialysis fluid - the balANZ trial. The Journal of the American Society of Nephrology 23(6):1097-1107, 2012.
Organisations Affected: Peritoneal dialysis units, **Year of Change:** 2012
Changes: This landmark multi-centre, multi-national randomised controlled trial demonstrated that neutral pH, low glucose degradation product peritoneal dialysis solutions provided important clinical benefits relative to conventional solutions, including better preservation of residual renal function, reduction in peritonitis rates and severity, and better preservation of peritoneal membrane function and ultrafiltration without any demonstrated harms.
Outcomes: The trial has resulted in a significant increase in prescription of biocompatible fluids in Australia, New Zealand and elsewhere.

(CIB) David Johnson, **Type:** Practice, **Year of Research Results:** 2010
Funding Source: NHMRC: Yes, Other Australian Source: Yes, International Source: Yes
Research: Harris A, Cooper BA, Li JJ, Bulfone L, Branley P, Collins JF, Craig JC, Fraenkel MB, Johnson DW, Kesselhut J, Luxton G, Pilmore A, Rosevear M, Tiller DJ, Pollock CA, Harris DC. Cost effectiveness of initiating dialysis early: A randomized controlled trial. American Journal of Kidney Disease 57(5):707-15, 2011.
Organisations Affected: European Best Practice Management Guidelines, **Year of Change:** 2011
Changes: This sub-study of the IDEAL trial demonstrated that planned, early initiation of dialysis in patients with end-stage kidney failure did not improve quality of life, but did significantly increase costs.
Outcomes: The results of this trial have led to a reversal of the global trend for early dialysis initiation and have been incorporated into the EBPM Guidelines

(CIB) David Johnson, **Type:** Practice, **Year of Research Results:** 2010
Funding Source: NHMRC: No, Other Australian Source: No, International Source: Yes
Research: Palmer SC, Navaneethan SD, Craig JC, Johnson DW, Tonelli M, Garg A, Johnson DW, Pellegrini F, Ravani P, Jardine M, Perkovic V, Graziano G, McGee R, Nicolucci A, Tognoni G, Strippoli GFM. Erythropoiesis-stimulating agents in people with chronic kidney disease: Systematic review and meta-analysis. Annals of Internal Medicine 153:23-33, 2010.
Organisations Affected: Renal units around the world., **Year of Change:** 2010
Changes: This systematic review, which incorporated the results of the TREAT trial, has led to a global reduction in haemoglobin targets and erythropoiesis stimulating agent prescription due to the findings that higher targets and/or ESA doses, promote harm (increased cardiovascular event risk).
Outcomes: Global usage of ESAs has reduced. The FDA has issued a black box warning advising against pushing haemoglobin values above 120 g/L on ESA therapy. The CARI, KDIGO and KDOQI guidelines all recommend against pushing haemoglobin values above 120 g/L on ESA therapy.

(CIB) David Johnson, **Type:** Practice, **Year of Research Results:** 2010
Funding Source: NHMRC: No, Other Australian Source: Yes, International Source: No
Research: van Eps CL, Jones M, Ng T, Johnson DW, Campbell SB, Isbel NM, Mudge DW, Beller E, Hawley CM. (2010). The Impact of extended hours home hemodialysis and buttonhole canjnulation technique on hospitalization rates for septic events related to dialysis access. Hemodialysis International 14(4):451-63, 2010.
Organisations Affected: Queensland Statweide Renal Clinical Network, **Year of Change:** 2010
Changes: This trial demonstrated that the buttonhole technique was associated with a significantly increased risk of bacteraemia.
Outcomes: A Queensland Health communique was circulated strongly recommending against buttonholing. This has changed practice throughout Queensland away from buttonholing to the ropeladder technique.

(CIB) David Johnson, **Type:** Practice, **Year of Research Results:** 2010
Funding Source: NHMRC: No, Other Australian Source: No, International Source: Yes
Research: Figuiredo A, Goh B, Jenkins S, Johnson DW, Mactier R, Ramalakshmi S, Shrestha B, Struijk D, Wilkie M. International Society of Peritoneal Dialysis Guidelines: Clinical practice guidelines for peritoneal access. Peritoneal Dialysis International 30:424-9, 2010.
Organisations Affected: International Society of Peritoneal Dialysis. Peritoneal dialysis units around the world., **Year of Change:** 2010
Changes: These guidelines have provided the best available evidence to guide management of peritoneal dialysis access.
Outcomes: These guidelines are used by the vast majority of peritoneal dialysis patients throughout the world.

(CIB) David Johnson, **Type:** Practice, **Year of Research Results:** 2009
Funding Source: NHMRC: No, Other Australian Source: No, International Source: Yes
Research: Brown EA, van Biesen W, Finkelstein F, Hurst H, Johnson DW, Kawanishi H, Pecoits-Filho R, Woodrow G. Length of time on peritoneal dialysis and encapsulating peritoneal sclerosis: Position paper for ISPD. Peritoneal Dialysis International 29(6):595-600, 2009.
Organisations Affected: International Society of Peritoneal Dialysis. Peritoneal dialysis units around the world., **Year of Change:** 2009
Changes: These guidelines provided advice regarding diagnosis, screening and management of encapsulating peritoneal sclerosis. They specifically advised against pre-emptively transferring PD patients to HD on the basis of time spent on PD.
Outcomes: These guidelines are used by the majority of peritoneal dialysis units throughout the world.

(CIB) David Johnson, **Type:** Practice, **Year of Research Results:** 2009
Funding Source: NHMRC: No, Other Australian Source: Yes, International Source: No
Research: McDonald SP, Marshall MR, Johnson DW, Polkinghorne K. Relationship of dialysis modality with mortality. Journal of the American Society of Nephrology 20(1):155-63, 2009.
Organisations Affected: ANZ Renal Units, **Year of Change:** 2009
Changes: This research demonstrated that selection of young (<60 years) patients without comorbidities for peritoneal dialysis was associated with improved outcomes.
Outcomes: Many units are reviewing their dialysis modality selection practices and attempting to preferentially steer young patients without comorbidities to PD.

(CIB) David Johnson, **Type:** Practice, **Year of Research Results:** 2007
Funding Source: NHMRC: No, Other Australian Source: Yes, International Source: No
Research: Badve SV, Hawley CM, McDonald SP, Mudge DW, Rosman JB, Brown FG, Johnson DW. A comparison of outcomes for automated versus continuous ambulatory peritoneal dialysis: Analysis of ANZDATA Registry. Kidney International 73(4): 480-8, 2007.
Organisations Affected: International Society of Peritoneal Dialysis, **Year of Change:** 2009
Changes: This research demonstrated that there were no differences in patient or technique survival rates in ANZ PD patients treated with APD versus CAPD.
Outcomes: Recommendations for use of APD have been softened to primarily lifestyle considerations and patient preference.

(CIB) David Johnson, **Type:** Practice, **Year of Research Results:** 2008
Funding Source: NHMRC: No, Other Australian Source: Yes, International Source: No
Research: Nicol DL, Preston JM, Wall DR, Griffin AD, Campbell SB, Isbel NM, Hawley CM, Johnson DW. Kidneys from patients with small renal tumours – a novel source of kidneys for transplantation. British Journal of Urology 102(2):188-92, 2008.
Organisations Affected: Queensland, Western Australia and Japanese renal transplant units., **Year of Change:** 2009
Changes: This research demonstrated that kidneys removed because of small (<3 cm) renal cell carcinomas were a safe and effective source of live donor kidneys for renal transplantation (1 recurrence in 50 kidneys transplanted).
Outcomes: The excellent results of our program has led to the establishment of this program in Western Australia and some Japanese Renal Transplant Units, thereby expanding the pool of available donor kidneys.

(CIB) David Johnson, **Type:** Practice, **Year of Research Results:** 2009
Funding Source: NHMRC: No, Other Australian Source: Yes, International Source: No
Research: O'Shea S, Duffull S, Johnson DW. Aminoglycosides in hemodialysis patients: is the current practice of post-dialysis dosing appropriate? Seminars in Dialysis 22(3):225-30, 2009.
This study demonstrated that pre-dialysis aminoglycoside dosing results in improved AUC characteristics compared with post-dialysis dosing.
Organisations Affected: ANZ Renal Units, **Year of Change:** 2009
Changes: A number of renal units, including RPA, are moving towards pre-dialysis gentamicin dosing of haemodialysis patients with infections.
Outcomes:

(CIB) David Johnson, **Type:** Practice, **Year of Research Results:** 2008
Funding Source: NHMRC: No, Other Australian Source: Yes, International Source: No
Research: Strippoli GFM, Navaneethan SD, Johnson DW, Perkovic V, Pellegrini F, Nicolucci A, Pansini F, Craig JC. Effects of statins in patients with chronic kidney disease: meta-analysis and meta-regression of randomised controlled trials. British Medical Journal 336(7645):645-51, 2008.
This systematic review supported an ongoing role for statins in reducing cardiovascular risk in patients with chronic kidney disease (especially early stages).
Organisations Affected: International Nephrology Clinical Practice Guidelines., **Year of Change:** 2009
Changes: Many international clinical practice guidelines (eg CARI, KDOQI, CSN, UK NICE, etc) continue to recommend statins for cardiovascular risk mitigation in CKD patients (especially stages 1-3).
Outcomes: The study's findings were incorporated into the KDIGO guidelines on lipid lowewring therapy.

(CIC) Jeremy Chapman, **Type:** Practice, **Year of Research Results:** 2011
Funding Source: NHMRC: Yes, Other Australian Source: No, International Source: No
Research: Management of kidney transplant recipients
Organisations Affected: KDIGO guidelines, CARI guidelines, **Year of Change:** 2011
Changes: Our research has demonstrated that induction with monoclonal IL2 blockers, when combined with standard triple-therapy immunosuppression, reduces acute rejection and graft loss, for up to 3 years post transplant, and is as effective and less harmful (lower risk of viral related complications), than polyclonal T-cell induction. Il-2 blockers have become standard therapy in Australia and Europe (90%). Similarly we showed that tacrolimus was superior to the alternative calcineurin-inhibitor, cyclosporine, leading to improved graft survival, with NODAT (new onset diabetes after transplantation) minimized if doses were minimized. Again, tacrolimus has become the standard of care. Finally, we also showed that routine prophylactic antivirals given after kidney transplantation, and not pre-emptive anti-viral use (administration only after a rise in viral load), reduces Cytomegalovirus (CMV) infection, CMV disease, and improves graft and patient survival. Again this has become standard care.
Outcomes: Substantial improvement in the care and outcomes.

(CIC) Jeremy Chapman, **Type:** Practice, **Year of Research Results:** 2010
Funding Source: NHMRC: Yes, Other Australian Source: No, International Source: No
Research: KDIGO guidelines on cancer and immunosuppression
Organisations Affected: KDIGO guidelines, **Year of Change:** 2010
Changes: Our research has convincingly demonstrated that cancer risk is increased patients with end-stage kidney disease and with kidney transplants. Through advancing on collaborative linkages both for data and for biostatistical and economic modelling, we have opened up new knowledge in the development of cancer. Prior to our work, the increased risk of cancer in CKD are considered solely through chemical immunosuppression. It is now clear that uraemia is a potent risk factor for cancer. New interventions have also been developed to prevent and reduce the risk of cancer in people with CKD. We have shown that the standard screening practices for cancers in the general population may not be applicable to patients with reduced kidney function.
Outcomes: Adaptation of the findings of this program of work into clinical practice guidelines (KDIGO) has substantially improved the efficiency and effectiveness of the care of kidney transplant recipients.

(CID) Carmel Hawley, **Type:** Policy, **Year of Research Results:** 2011
Funding Source: NHMRC: No, Other Australian Source: No, International Source: Yes
Research: Nesrallah, G. E.; Suri, R. S.; Moist, L. M.; Cuerden, M.; Groeneweg, K. E.; Hakim, R.; Ofsthun, N. J.; McDonald, S. P.; Hawley, C.; Caskey, F. J.; Couchoud, C.; Awaraji, C.; Lindsay, R. M. International Quotidian Dialysis Registry: annual report 2009 Hemodial Int (2009) 13 3 240-9
Organisations Affected: International dialysis units, **Year of Change:** 2011
Changes: This report and guideline informed practice on risks, benefits, and technical conduct of long hours dialysis
Outcomes:

(CID) Carmel Hawley, **Type:** Policy, **Year of Research Results:** 2010
Funding Source: NHMRC: No, Other Australian Source: Yes, International Source: No
Research: Govindarajulu S, Hawley CM, McDonald SP, Brown FG, Rosman JB, Wiggins KJ, Bannister K, Johnson DW. Staphylococcus aureus peritonitis in Australian peritoneal dialysis patients: predictors, treatment and outcomes in 503 cases. Perit Dial Int 2010; 30:311–19. Findings of the study were incorporated into the ISPD GUIDELINES/RECOMMENDATIONS: PERITONEAL DIALYSIS-RELATED INFECTIONS RECOMMENDATIONS: 2010 UPDATE,
Organisations Affected: International Society of Peritoneal Dialysis, **Year of Change:** 2010
Changes: This study was the largest ever report on the associations, and outcomes of Staphylococcus aureus related peritoneal dialysis peritonitis and informed the ISPD guidelines about poorer outcomes with MRSA vs. MSSA and that the use of Vancomycin vs. Cephazolin in the initial regimen was not predictive of outome.
Outcomes:

(CID) Carmel Hawley, **Type:** Policy, **Year of Research Results:** 2009
Funding Source: NHMRC: No, Other Australian Source: Yes, International Source: No
Research: Barraclough K, Hawley CM, McDonald SP, Brown FG, Rosman JB, Wiggins KJ, bannister K, Johnson DW. Corynebacterium peritonitis in Australian peritoneal dialysis patients: predictors, treatment and outcomes in 82 cases. Nephrol Dial Transplant 2009; 24:3834–9. Findings of the study were incorporated into the ISPD GUIDELINES/RECOMMENDATIONS: PERITONEAL DIALYSIS-RELATED INFECTIONS RECOMMENDATIONS: 2010 UPDATE
Organisations Affected: International Society of Peritoneal Dialysis, **Year of Change:** 2010
Changes: This study was the largest ever report on the associations, and outcomes of corynebactium related peritoneal dialysis peritonitis and demonstrated that this entity had generally favourable outcomes and informed the ISPD guidelines on this favourable outcome and the importance of adequate antibiotic therapy
Outcomes:

(CID) Carmel Hawley, **Type:** Policy, **Year of Research Results:** 2009
Funding Source: NHMRC: No, Other Australian Source: Yes, International Source: No
Research: Fahim M, Hawley CM, McDonald SP, Brown FG, Rosman JB, Wiggins KJ, Bannister KM, Johnson DW. Culture-negative peritonitis in peritoneal dialysis patients in Australia: predictors, treatment, and outcomes in 435 cases. Am J Kidney Dis. 2010; 55:690-7
Organisations Affected: International Society of Peritoneal Dialysis, **Year of Change:** 2010
Changes: Research findings incorporated into "ISPD GUIDELINES/RECOMMENDATIONS - PERITONEAL DIALYSIS-RELATED INFECTIONS RECOMMENDATIONS: 2010 UPDATE" Perit Dial Int 2010; 30:393–423.
These guidelines are used internationally as the primary guide to the management of peritoneal dialysis related infections. Our cohort study provided contemporary information on the outcome of culture negative peritonitis and areas that should be explored/modified to limit the rate of this entity. This information was incorporated into the ISPD guidelines.
Outcomes:

(CID) Carmel Hawley, **Type:** Policy, **Year of Research Results:** 2006
Funding Source: NHMRC: Yes, Other Australian Source: No, International Source: No
Research: Johnson DW, Wong J, Wiggins KJ, Kirwan R, Griffin A, Preston J, Wall D, Campbell SB, Isbel NM, Mudge DW, Hawley CM, Nicol DL. A randomized, controlled trial of coiled versus straight swan neck Tenckhoff catheters in peritoneal dialysis patients. American Journal of Kidney Disease 48(5):812-21, 2006.
This research demonstrated that coiled Tenckhoff catheters are associated with a higher risk of technique failure compared with straight catheters
Organisations Affected: International Society of Peritoneal Dialysis, **Year of Change:** 2009
Changes: The ISPD Peritoneal Dialysis Access Guidelines have recommended caution with the use of colied PD catheters
Outcomes:

(CID) Carmel Hawley, **Type:** Practice, **Year of Research Results:** 2014
Funding Source: NHMRC: No, Other Australian Source: Yes, International Source: No
Research: Muir CA, Kotwal SS, Hawley CM, Polkinghorne K, Gallagher MP, Snelling P, Jardine MJ. Buttonhole cannulation: clinical outcomes in a home haemodialysis cohort and systematic review. CJASN 2014; 9(1): 110-119
Organisations Affected: All ANZ renal units, **Year of Change:** 2014
Changes: Reviewed and appraised the published data on the hazards associated with buttonhole cannulation, and therefore has the potential to influence clinical practice.
Outcomes:

(CID) Carmel Hawley, **Type:** Practice, **Year of Research Results:** 2013
Funding Source: NHMRC: No, Other Australian Source: No, International Source: Yes
Research: Brimble KS, Mehrotra R, Tonelli M, Hawley CM, Castledine C, McDonald SP, Levidiotis V, Gangji AS, Treleaven DJ, Margetts PJ, Walsh M. Estimated GFR reporting influences recommendations for dialysis initiation. J Am Soc Nephrol 2013; 24(11): 1737-1742.
Organisations Affected: Global renal units, **Year of Change:** 2013
Changes: Will play an important role in influencing clinical practice; this paper examined the role of reporting of GFR in decision making by clinicians, which provides an important message for clinical practice.
Outcomes:

(CID) Carmel Hawley, **Type:** Practice, **Year of Research Results:** 2010
Funding Source: NHMRC: No, Other Australian Source: Yes, International Source: No
Research: Barraclough, K. A.; Wiggins, K. J.; Hawley, C. M.; van Eps, C. L.; Mudge, D. W.; Johnson, D. W.; Whitby, M.; Carpenter, S.; Playford, E. G. Intradermal versus intramuscular hepatitis B vaccination in hemodialysis patients: a prospective open-label randomized controlled trial in nonresponders to primary vaccination Am J Kidney Dis (2009) 54 1 95-103
Organisations Affected: Australian and New Zealand Dialysis units, **Year of Change:** 2010
Changes: This landmark reserach showed that intradermal Hepatitis B vacination in dialysis patients who did not respond to an initial intramuscular vaccination was more effective than persistent intramuscular boosters and has changed practices across units for this important infection control practice
Outcomes:

(CID) Carmel Hawley, **Type:** Practice, **Year of Research Results:** 2008
Funding Source: NHMRC: No, Other Australian Source: Yes, International Source: No
Research: Nicol DL, Preston JM, Wall DR, Griffin AD, Campbell SB, Isbel NM, Hawley CM, Johnson DW. Kidneys from patients with small renal tumours – a novel source of kidneys for transplantation. British Journal of Urology 102(2):188-92, 2008.
This research demonstrated that kidneys removed because of small (<3 cm) renal cell carcinomas were a safe and effective source of live donor kidneys for renal transplantation (1 recurrence in 50 kidneys transplanted).
Organisations Affected: Queensland, Western Australia and Japanese renal transplant units, **Year of Change:** 2009
Changes: The excellent results of our program has led to the establishment of this program in Western Australia and some Japanese Renal Transplant Units, thereby expanding the pool of available donor kidneys
Outcomes:

(CID) Carmel Hawley, **Type:** Practice, **Year of Research Results:** 2007
Funding Source: NHMRC: No, Other Australian Source: Yes, International Source: No
Research: Badve SV, Hawley CM, McDonald SP, Mudge DW, Rosman JB, Brown FG, Johnson DW. A comparison of outcomes for automated versus continuous ambulatory peritoneal dialysis: Analysis of ANZDATA Registry. Kidney International 73(4): 480-8, 2007.
This research demonstrated that there were no differences in patient or technique survival rates in ANZ PD patients treated with APD versus CAPD.,
Organisations Affected: International Society of Peritoneal Dialysis, **Year of Change:** 2009
Changes: Recommendations for use of APD have been softened to primarily lifestyle considerations and patient preference
Outcomes:

(CIE) Stephen McDonald, **Type:** Policy, **Year of Research Results:** 2014
Funding Source: NHMRC: No, Other Australian Source: Yes, International Source: No
Research: Outcomes of work (including a number of analyses specifically created for this publication) into recent trends (i.e.stabilisation of) in incidence rates for end-stage kidney disease and practice patterns (waning of peritoneal dialysis rates in some sectors) for dialysis underpin the "State of the Nation" report from Kidney Health Australia.
Organisations Affected: Kidney Health Australia, **Year of Change:** 2014
Changes: Creation of document summarising recent trends
Advocacy for increased funding for home dialysis and transplantation
Outcomes:

(CIE) Stephen McDonald, **Type:** Policy, **Year of Research Results:** 2013
Funding Source: NHMRC: Yes, Other Australian Source: Yes, International Source: No
Research: Grace, BS, Clayton, PA, Cass, A, McDonald, SP: Transplantation rates for living- but not deceased-donor kidneys vary with socioeconomic status in Australia. Kidney Int, 83: 138-145, 2013
Organisations Affected: Commonwealth government; Kidney Health Australia, **Year of Change:** 2013
Changes: Results used by KHA to successfully advocate for introduction of scheme to reimburse living donors for out of pocket expenses.
Outcomes: Introduction of reimbursement scheme

(CIE) Stephen McDonald, **Type:** Policy, **Year of Research Results:** 2013
Funding Source: NHMRC: No, Other Australian Source: Yes, International Source: No
Research: Sparke, C, Moon, L, Green, F, Mathew, T, Cass, A, Chadban, S, Chapman, J, Hoy, W, McDonald, S: Estimating the total incidence of kidney failure in Australia including individuals who are not treated by dialysis or transplantation. Am J Kidney Dis, 61: 413-419, 2013.
Organisations Affected: AIHW, **Year of Change:** 2012
Changes: Methodology described in this paper of total burden of kidney disease developed & introduced to for Comonwealth "Reducing the Gap" reporting
Outcomes:

(CIE) Stephen McDonald, **Type:** Policy, **Year of Research Results:** 2010
Funding Source: NHMRC: No, Other Australian Source: Yes, International Source: No
Research: van Leeuwen, MT, Webster, AC, McCredie, MRE, Stewart, JH, McDonald, SP, Amin, J, Kaldor, JM, Chapman, JR, Vajdic, CM, Grulich, AE: Effect of reduced immunosuppression after kidney transplant failure on risk of cancer: population based retrospective cohort study. BMJ, 340: c570-, 2010.
Organisations Affected: CARI guideline (Australian renal guidelines), **Year of Change:** 2011
Changes: FIndings of outcomes of reduced immunosupression on cancer risk incorporated into guidelines for care of cancer post transplantation
Outcomes:

(CIE) Stephen McDonald, **Type:** Policy, **Year of Research Results:** 2008
Funding Source: NHMRC: No, Other Australian Source: Yes, International Source: No
Research: van Leeuwen, MT, Grulich, AE, McDonald, SP, McCredie, MRE, Amin, J, Stewart, JH, Webster, AC, Chapman, JR, Vajdic, CM: Immunosuppression and other risk factors for lip cancer after kidney transplantation. Cancer Epidemiology, Biomarkers and Prevention, 2008.
Organisations Affected: CARI guidelines (Australia renal guidelines), **Year of Change:** 2011
Changes: FIndings of high rates of lip cancer post transplant incorporated into recommendatins for post-transplant care
Outcomes:

(CIE) Stephen McDonald, **Type:** Policy, **Year of Research Results:** 2002
Funding Source: NHMRC: No, Other Australian Source: No, International Source: No
Research: McDonald, SP, Russ, GR: Survival of recipients of cadaveric kidney transplants compared with those receiving dialysis treatment in Australia and New Zealand, 1991-2001. Nephrol Dial Transplant, 17: 2212-2219, 2002.
Organisations Affected: Transplantation Society of Australia and NZ, **Year of Change:** 2011
Changes: Results incorporated into national consensus guidelines eligibility for kidney transplantation
Outcomes:

(CIE) Stephen McDonald, **Type:** Policy, **Year of Research Results:** 2011
Funding Source: NHMRC: No, Other Australian Source: Yes, International Source: No
Research: Stewart, JH, Vajdic, CM, van Leeuwen, MT, Amin, J, Webster, AC, Chapman, JR, McDonald, SP, Grulich, AE, McCredie, MRE: The pattern of excess cancer in dialysis and transplantation. Nephrol Dial Transplant, 24: 3225-3231, 2009. AND Vajdic, CM, McDonald, SP, McCredie, MRE, van Leeuwen, MT, Stewart, JH, Law, M, Chapman, JR, Webster, AC, Kaldor, JM, Grulich, AE: Cancer Incidence Before and After Kidney Transplantation. JAMA, 296: 2823-2831, 2006.
Organisations Affected: CARI guidelines (Australian renal guidelines), **Year of Change:** 2011
Changes: Findings of patterns of cancer post kidney transplantation informed recommendations about cancer screening post transplantation
Outcomes:

(CIE) Stephen McDonald, **Type:** Policy, **Year of Research Results:** 2010
Funding Source: NHMRC: No, Other Australian Source: Yes, International Source: No
Research: Govindarajulu, S, Hawley, CM, McDonald, SP, Brown, F, Rosman, J, Wiggins, KJ, Bannister, K, Johnson, DW: Staphylococcus Aureus Peritonitis in Australian Peritoneal Dialysis Patients: Predictors, Treatment, and Outcomes in 503 Cases. Perit Dial Int, 30: 311-319, 2010
Organisations Affected: International Society of Peritoneal Dialysis, **Year of Change:** 2010
Changes: Findings of the study (the largest on associations and outcomes of Staph Aureus PD peritonitis) in particular poorer outcome of MRSA vs MSSA and good results with cephazolin were incorporated into the ISPD guidelines on treatment of peritonitis
Outcomes: Evidence base for international guideline

(CIE) Stephen McDonald, **Type:** Policy, **Year of Research Results:** 2010
Funding Source: NHMRC: No, Other Australian Source: Yes, International Source: No
Research: Fahim, M, Hawley, CM, McDonald, SP, Brown, FG, Rosman, JB, Wiggins, KJ, Bannister, KM, Johnson, DW: Culture-Negative Peritonitis in Peritoneal Dialysis Patients in Australia: Predictors, Treatment, and Outcomes in 435 Cases. Am J Kidney Dis, 55: 690-697, 2010. Demonstrated favourable outcomes of the type of PD related peritonitis
Organisations Affected: International Society of Peritoneal Dialysis, **Year of Change:** 2010
Changes: Informed guidelines on the favourable outcome of this form of peritonitis with antibiotic therapy and the importance of assessing culture technique if culture negative rates were high
Outcomes: Incorporation of results into ISPD guidelines

(CIE) Stephen McDonald, **Type:** Policy, **Year of Research Results:** 2010
Funding Source: NHMRC: No, Other Australian Source: Yes, International Source: No
Research: Barraclough, K, Hawley, CM, McDonald, SP, Brown, FG, Rosman, JB, Wiggins, KJ, Bannister, KM, Johnson, DW: Corynebacterium peritonitis in Australian peritoneal dialysis patients: predictors, treatment and outcomes in 82 cases. Nephrol Dial Transplant, 24: 3834-3839, 2009.
Organisations Affected: International Society of Peritoneal Dialysis, **Year of Change:** 2010
Changes: Demonstration of favourable outcomes of Corynebacterium peritonitis informed ISPD guidelines on need for adequate antibiotic therapy but not necessarily catheter removal
Outcomes: Findings incorporated into international ISPD guidelines "Recommendations: peritoneal dialysis-related infections: 2010 update"

(CIE) Stephen McDonald, **Type:** Policy, **Year of Research Results:** 2008
Funding Source: NHMRC: No, Other Australian Source: Yes, International Source: No
Research: As part of a consortium, developed the Statewide Renal Services Plan for Tasmania. Specific responsibility of Dr McDonald was development of the projections of kidney disease for Tasmania. This project was performed under contract for Tasmanian government. the final report included several dozen recommendation under 4 main aims: 1) Provide a coordinated and responsive approach to service delivery 2) Integrate renal services across the continuum of care 3) Develop a sustainable and equitable statewide renal service and 4) Maintain an Efficient and Effective Service.
Organisations Affected: Tasmanian Ministry of Health, Hobart and Launceston Hospitals, **Year of Change:** 2010
Changes: Changes recommended to planning of future renal services and location of dialysis services. These include increase in numebr of nephrologists, the increase in availability of forms of home dialysis, and promotion of transplantation. Implementation of other changes is still underway, as fuinding and staffing resources become available.
Outcomes: Progressive implementation of changes has been undertaken by the Tasmanian government. In part this has been limited by funding availability. Important elements of progress include increasing emphasis of avaialbility of home dialysis and transplant numbers, although these proportions (especially home haemodialyis) still remain low compared to some othe states.

(CIE) Stephen McDonald, **Type:** Policy, **Year of Research Results:** 2010
Funding Source: NHMRC: No, Other Australian Source: Yes, International Source: No
Research: Howard, K, Salkeld, G, White, S, McDonald, S, Chadban, S, Craig, JC, Cass, A: The cost-effectiveness of increasing kidney transplantation and home-based dialysis. Nephrology, 14: 123-132, 2009.
Organisations Affected: Commonwealth government; Kidney Health Australia, **Year of Change:** 2009
Changes: This (and associated sections of ANZDATA Reports) underpin the policy changes and injections of funding into increased home dialysis and transplantation
Outcomes: Increased rates of kidney transplantation and home dialysis in 2010-12

(CIE) Stephen McDonald, **Type:** Practice, **Year of Research Results:** 2012
Funding Source: NHMRC: No, Other Australian Source: Yes, International Source: No
Research: Collins, MG, Teo, E, Cole, SR, Chan, C-Y, McDonald, SP, Russ, GR, Young, GP, Bampton, PA, Coates, PT: Screening for colorectal cancer and advanced colorectal neoplasia in kidney transplant recipients: cross sectional prevalence and diagnostic accuracy study of faecal immunochemical testing for haemoglobin and colonoscopy. BMJ, 345, 2012
Organisations Affected: SA/NT Renal Transplant Service, **Year of Change:** 2013
Changes: Introduction of screening colonoscopy for transplant recipients > 50 years
Outcomes:

(CIE) Stephen McDonald, **Type:** Practice, **Year of Research Results:** 2011
Funding Source: NHMRC: No, Other Australian Source: Yes, International Source: No
Research: Badve, SV, Hawley, CM, McDonald, SP, Brown, FG, Boudville, NC, Wiggins, KJ, Bannister, KM, Johnson, DW: Use of aminoglycosides for peritoneal dialysis-associated peritonitis does not affect residual renal function. Nephrol Dial Transplant, 27: 381-387, 2011
Organisations Affected: CNARTS and other Australian renal units, **Year of Change:** 2012
Changes: Reincorporation of gentamicin into PD treatment algorithms after demonstration that intraperitoneal use for treatment of peritonitis does not affect residual renal function
Outcomes:

(CIE) Stephen McDonald, **Type:** Practice, **Year of Research Results:** 2012
Funding Source: NHMRC: No, Other Australian Source: Yes, International Source: No
Research: Lim, WH, Boudville, N, McDonald, SP, Gorham, G, Johnson, DW, Jose, M: Remote indigenous peritoneal dialysis patients have higher risk of peritonitis, technique failure, all-cause and peritonitis-related mortality. Nephrol Dial Transplant, 26: 3366-3372, 2011
Organisations Affected: Austalian renal units (especially in SA, NT and WA), **Year of Change:** 2012
Changes: Findings have led to preference home haemodialysis (rather than peritoneal dialysis) as a strategy for providing treatment for remote Aboriginal people with ESKD by the units that traet the bulk of people in this group
Outcomes:

(CIE) Stephen McDonald, **Type:** Practice, **Year of Research Results:** 2011
Funding Source: NHMRC: No, Other Australian Source: Yes, International Source: No
Research: Ghali, JR, ...Johnson, DW, McDonald, SP: Microbiology and Outcomes of Peritonitis in Australian Peritoneal Dialysis Patients. Perit Dial Int, 31: 651-662, 2011.
Organisations Affected: Aust nephrology community, **Year of Change:** 2010
Changes: Documentation of high peritonitis rates in Australia; led to progam to increase awareness and reduced national peritonitis rates. Change began prior to publication (with presentation & discussion of abstract results)
Outcomes: Reduction in reported rates of PD peritonitis in Australia
Introduction of peritonitis as part of dialysis KPI program

(CIE) Stephen McDonald, **Type:** Practice, **Year of Research Results:** 2008
Funding Source: NHMRC: No, Other Australian Source: Yes, International Source: No
Research: Badve, SV, Hawley, CM, McDonald, SP, Mudge, DW, Rosman, JB, Brown, FG, Johnson, DW: Automated and continuous ambulatory peritoneal dialysis have similar outcomes. Kidney Int, 73: 480-488, 2008.
Organisations Affected: International Society of Peritoneal Dialysis, **Year of Change:** 2009
Changes: Recommendations for use of APD softened to primarily lifestyle considerations / patient preference.
Outcomes:

CV-JR: Editorial Responsibilities (last 5 years only)

Team Member	This entry type	Journal/Publication Name	Role	Duration	Number of Articles (for Peer Review only)
(CIA) Jonathan Craig	Member of an Editorial Board - Journal/Book	Nephrology	Editorial Board Member	01/01/2010 -	
	Member of an Editorial Board - Journal/Book	Nephrology	Associate Editor	01/01/2010 -	4
	Peer Reviewer - Journal Article	Evidence Based Paediatrics and Child Health	Associate Editor	01/01/2008 - 31/12/2010	12
	Member of an Editorial Board - Journal/Book	American Journal of Kidney Diseases	Associate Editor	01/01/2007 -	
	Member of an Editorial Board - Journal/Book	Journal of Paediatrics and Child Health	Editorial Board Member	01/01/2004 - 31/12/2010	35
	Member of an Editorial Board - Journal/Book	Nephrology	Associate Editor	01/01/2003 - 31/12/2010	80
	Member of an Editorial Board - Journal/Book	Cochrane Renal Group	Chief Editor	01/01/2001 -	120
(CIB) David Johnson	Member of an Editorial Board - Journal/Book	American Journal of Kidney Disease	Editorial Board Member	25/04/2014 - 30/06/2019	6
	Peer Reviewer - Journal Article	Kidney Research and Blood Pressure	Guest	01/10/2012 -	1
	Peer Reviewer - Journal Article	Open Drug Discovery Journal	Guest	08/07/2009 -	2
	Peer Reviewer - Journal Article	Journal of Infection	Guest	17/06/2009 -	2
	Peer Reviewer - Journal Article	Future Medicines	Guest	13/11/2008 -	2
	Peer Reviewer - Journal Article	Nature Clinical Practice Nephrology	Guest	15/10/2008 -	5

	Peer Reviewer - Journal Article	Nature Reviews Nephrology	Guest	24/06/2008 -	4
	Member of an Editorial Board - Journal/Book	Journal of the American Board of Family Medicine	Editorial Board Member	19/05/2008 -	
	Member of an Editorial Board - Journal/Book	Open Urology Nephrology Journal	Editorial Board Member	12/05/2008 -	3
	Peer Reviewer - Journal Article	Clinical Journal of the American Society of Nephrology		31/12/2007 -	11
	Peer Reviewer - Journal Article	Blood Purification	Guest	12/04/2005 -	3
	Peer Reviewer - Journal Article	Lancet	Other	14/02/2005 -	4
	Member of an Editorial Board - Journal/Book	Hemodialysis International	Editorial Board Member	17/01/2005 -	3
	Member of an Editorial Board - Journal/Book	Evidence-Based Nephrology	Associate Editor	03/01/2005 -	
	Peer Reviewer - Journal Article	Medical Science Monitor	Guest	19/09/2005 -	3
	Peer Reviewer - Journal Article	Cytokine	Guest	08/06/2004 -	2
	Peer Reviewer - Journal Article	American Journal of Kidney Diseases	Guest	09/02/2004 -	12
	Member of an Editorial Board - Journal/Book	Peritoneal Dialysis International	Editorial Board Member	10/02/2003 -	21
	Peer Reviewer - Journal Article	Journal of the American Society of Nephrology	Guest	03/04/2000 -	17
	Peer Reviewer - Journal Article	Kidney International	Guest	03/01/2000 -	40
	Peer Reviewer - Journal Article	Nephrology Dialysis and Transplantation	Guest	04/10/1999 -	60
	Peer Reviewer - Journal Article	Australian and New Zealand Journal of Medicine / Internal Medicine Journal	Guest	18/01/1999 -	25

	Peer Reviewer - Journal Article	Experimental Nephrology	Guest	23/08/1999 -	10
	Peer Reviewer - Journal Article	Nephrology	Guest	08/03/1999 -	40
	Peer Reviewer - Journal Article	Nephron	Other	17/08/1998 -	12
(CIC) Jeremy Chapman	Member of an Editorial Board - Journal/Book	Transplantation	Chief Editor	01/01/2014 - 01/01/2020	
	Member of an Editorial Board - Journal/Book	Transplantation	Associate Editor	01/01/2013 - 01/01/2014	
	Member of an Editorial Board - Journal/Book	Transplantation Proceedings	Editorial Board Member	01/01/2010 -	
	Member of an Editorial Board - Journal/Book	Experimental and Clinical Transplantation	Editorial Board Member	01/01/2009 - 31/12/2009	
	Member of an Editorial Board - Journal/Book	Clinical Transplantation	Editorial Board Member	01/01/2008 -	
	Member of an Editorial Board - Journal/Book	Journal of the American Society of Nephrology (Clinical)	Editorial Board Member	01/01/2008 -	
	Member of an Editorial Board - Journal/Book	Hong Kong Journal of Nephrology	Editorial Board Member	01/01/2006 -	
	Member of an Editorial Board - Journal/Book	Tranplant International	Editorial Board Member	01/01/2005 -	
	Member of an Editorial Board - Journal/Book	Nephrology	Editorial Board Member	01/01/2004 -	
	Member of an Editorial Board - Journal/Book	American Journal of Transplantation	Editorial Board Member	01/01/2003 -	
	Member of an Editorial Board - Journal/Book	Australian and New Zealand Journal of Medicine	Editorial Board Member	01/01/2001 -	
	Member of an Editorial Board - Journal/Book	Transplantation Reviews	Chief Editor	01/01/1999 - 01/01/2015	
	Member of an Editorial Board - Journal/Book	Marrow Donors Worldwide	Editorial Board Member	01/01/1994 -	

	Member of an Editorial Board - Journal/Book	Transplantation	Editorial Board Member	01/01/1989 - 01/01/2013	
(CID) Carmel Hawley	Peer Reviewer - Journal Article	American Journal of Kidney Diseases	Guest	01/01/2013 -	
	Peer Reviewer - Journal Article	Clinical Nephrology	Guest	01/01/2010 -	1
	Peer Reviewer - Journal Article	Hemodialysis International	Guest	01/01/2009 -	2
	Peer Reviewer - Journal Article	Medical Journal of Australia Reviews	Other	01/01/2004 - 16/03/2010	2
	Member of an Editorial Board - Journal/Book	Nephrology	Associate Editor	01/01/2003 - 31/12/2013	20
	Peer Reviewer - Journal Article	Nephrology Dialysis Transplantation	Guest	01/01/2002 -	
	Peer Reviewer - Journal Article	Australian and New Zealand Journal of Medicine	Guest	01/01/1999 -	
	Peer Reviewer - Journal Article	Nephrology	Guest	01/01/1999 -	
(CIE) Stephen McDonald	Peer Reviewer - Journal Article	International Journal of Evidence-based Healthcare		20/01/2013 -	1
	Peer Reviewer - Journal Article	Pediatrics		14/11/2012 -	1
	Peer Reviewer - Journal Article	Postgraduate Medical Journal		04/09/2012 -	1
	Peer Reviewer - Journal Article	American Journal of Transplantation		01/07/2012 -	4
	Peer Reviewer - Journal Article	PLoS one		21/06/2012 -	1
	Peer Reviewer - Journal Article	Transplantation	Other	01/01/2012 -	3
	Peer Reviewer - Journal Article	Kidney International		01/01/2012 -	5

	Peer Reviewer - Journal Article	Lancet	Other	01/01/2009 -	1
	Peer Reviewer - Journal Article	JAMA		01/01/2009 -	4
	Peer Reviewer - Journal Article	Australian and New Zealand Journal of public Health		02/02/2008 -	1
	Peer Reviewer - Journal Article	American Journal of Kidney Diseases		01/01/2008 -	5
	Peer Reviewer - Journal Article	Nephrology Dialysis & Transplantation	Other	01/01/2006 -	5
	Member of an Editorial Board - Journal/Book	Nephrology	Associate Editor	31/01/2005 -	

CV-CN: Contribution to NHMRC (last 5 years only)

Team Member	Contribution Role	Year	Number of times
(CIA) Jonathan Craig	Peer Review Panel - As a Member	2014	1
	Research Translation Faculty	2014	1
	Peer Review Panel - As a Chair/Deputy Chair	2014	1
	Peer Review Panel - As a Chair/Deputy Chair	2013	1
	Peer Review Panel - As a Chair/Deputy Chair	2012	1
	Peer Review Panel - As a Chair/Deputy Chair	2010	1
	External Assessment - External Assessor	2010	1
	Peer Review Panel - As a Chair/Deputy Chair	2009	1
	External Assessment - External Assessor	2009	1
(CIB) David Johnson	External Assessment - External Assessor	2014	2
	Research Translation Faculty	2014	1
	External Assessment - External Assessor	2013	6
	External Assessment - External Assessor	2012	6
	External Assessment - External Assessor	2011	1
	Selector/Academy	2011	1
	Selector/Academy	2010	
	External Assessment - External Assessor	2010	6
	Grant Advisory Group - As a Member	2009	
(CIC) Jeremy Chapman	Research Committee Member	2012	1
	Other Principal Committee Chair	2011	1
	Research Committee Member	2011	1
	Other Principal Committee Chair	2010	2
	Research Committee Member	2010	1

	Research Committee Member	2009	1
(CID) Carmel Hawley	Research Translation Faculty	2014	1
	External Assessment - External Assessor	2013	1
	External Assessment - External Assessor	2012	1
	External Assessment - External Assessor	2011	1
	External Assessment - External Assessor	2010	1
	External Assessment - External Assessor	2009	1

CV-CP: Conference Participation (last 5 years only)

(CIA) Jonathan Craig

Asian Congress of Pediatric Nephrology; International; India
Role: Keynote Speaker; **Year:** 2014
Conference Presentation Summary: Synthesizing evidence: Cochrane Renal Group

Asian Congress of Paediatric Nephrology; International; India
Role: Keynote Speaker; **Year:** 2014
Conference Presentation Summary: Steroid sensitive nephrotic syndrome: Evidence based therapy

The Royal College of Pathologists of Australasia Pathology Update Meeting; National; Australia
Role: Invited Speaker; **Year:** 2013
Conference Presentation Summary: Cardiac testing in chronic kidney disease: more good than harm

Australian and New Zealand Society of Nephrology Annual Scientific Meeting; National; Australia
Role: Invited Speaker; **Year:** 2013
Conference Presentation Summary: Systematic reviews and clinical decision making

2013 Australasian Conference Symposium; National; Australia
Role: Plenary Speaker; **Year:** 2013
Conference Presentation Summary: Cochrane structures 20 years on: a 747 in an era of A380s

16th Congress of the International Paediatric Nephrology Association; International; China
Role: Panel Chair; **Year:** 2013
Conference Presentation Summary: Keynote trials in Paediatric Nephrology

16th Congress of the International Paediatric Nephrology Association (IPNA); International; China
Role: Invited Speaker; **Year:** 2013

Conference Presentation Summary: Nephrotic Syndrome: Progress in Therapy.

International Pediatric Association Congress; International; China
Role: Conference Committee; **Year:** 2013
Conference Presentation Summary: Scientific Program Committee Member

African Cochrane Indaba - Global Evidence Local Application; International; South Africa
Role: Keynote Speaker; **Year:** 2013
Conference Presentation Summary: 20 years on: The contribution of the Cochrane Collaboration to healthcare worldwide

General Paediatric Update; National; Australia
Role: Keynote Speaker; **Year:** 2012
Conference Presentation Summary: Keynote presentation: “Prophylactic Antibiotics in Children Following a Urinary Tract Infection”

Workshop Program: “Antenatally Detected Kidney Problems”

Cochrane Canada Symposium; International; Canada
Role: Plenary Speaker; **Year:** 2012
Conference Presentation Summary: Progress towards equity in the evidence

3rd Annual International STaR Symposium; International; Canada
Role: Invited Speaker; **Year:** 2012
Conference Presentation Summary: The Next Steps for STaR Child Health

KDIGO Controversies Conference in Acute Kidney Injury; International; China
Role: Invited Speaker; **Year:** 2012
Conference Presentation Summary: KDIGO Clinical practice guideline on CKD

Cochrane Colloquium; International; New Zealand
Role: Conference Committee; **Year:** 2012
Conference Presentation Summary: Member, Scientific Program Committee

20th Cochrane Colloquium; International; New Zealand
Role: Plenary Speaker; **Year:** 2012
Conference Presentation Summary: Almost 20 years of global evidence – did it help?

The Paediatric Society of New Zealand 64th Annual Scientific Meeting; International; New Zealand
Role: Keynote Speaker; **Year:** 2012
Conference Presentation Summary: Trials in Children

The Paediatric Society of New Zealand 64th Annual Scientific Meeting; International; New Zealand
Role: Plenary Speaker; **Year:** 2012
Conference Presentation Summary: Nephrotic syndrome: evidence based update

The 3rd CRIAH Aboriginal Health Research Conference; National; Australia
Role: Keynote Speaker; **Year:** 2011
Conference Presentation Summary: Theme Session: "Effective Partnerships Between Aboriginal Communities and Researchers: Child Health"

The 7th World Congress of the World Society for Pediatric Infectious Diseases; International; Australia
Role: Invited Speaker; **Year:** 2011
Conference Presentation Summary: 1. Invited speaker to present on "Antibiotic prophylaxis for paediatric UTI" at the Consensus Symposium
2. Pediatric UTI – Management and Prevention in 2011

World Congress of Nephrology; International; Canada
Role: Conference Committee; **Year:** 2011
Conference Presentation Summary: Member, Scientific Program Committee

International Society of Nephrology and the Canadian Society of Nephrology, World Congress of Nephrology; International; Canada
Role: Keynote Speaker; **Year:** 2011
Conference Presentation Summary: Theme symposia session: Use of clinical evidence in dialysis: where to go from here?

Croatian Cochrane Symposium; International; Croatia
Role: Keynote Speaker; **Year:** 2011
Conference Presentation Summary: The role of the Cochrane Collaboration in modern medical practice and health care

KDIGO Controversies Conference on Haemodialysis; International; France
Role: Invited Speaker; **Year:** 2011
Conference Presentation Summary: Haemodialysis: frequency, duration or dose

16th Scientific Meeting of the Philippines Society of Pediatric Nephrology; International; Philippines
Role: Keynote Speaker; **Year:** 2011
Conference Presentation Summary: Evidence based practice for paediatric nephrology: UTI and nephrotic syndrome

American Society of Nephrology (ASN) 44th Annual Meeting; International; United States
Role: Invited Speaker; **Year:** 2011
Conference Presentation Summary: Invited speaker for the Clinical Nephrology Conference titled "Glomerulonephritis Treatment Guidelines". Lecture title: "the strengths and weaknesses of guidelines"

World Society for Pediatric Infectious Disease (WSPID); National; Australia
Role: Invited Speaker; **Year:** 2010
Conference Presentation Summary: Invited to talk on "Antibiotic prophylaxis for pediatric UTI"

The Joanna Briggs Institute Convention; National; Australia
Role: Plenary Speaker; **Year:** 2010

Conference Presentation Summary: Opening plenary session: the 'means' of the mission: the evidence; Topic: capturing and disseminating the best available evidence

8th Winter Symposium of the Christian Medical College and the 3rd South Asian Regional Symposium; International; India
Role: Invited Speaker; **Year:** 2010
Conference Presentation Summary: Presentations:
1. Systematic Reviews and Meta Analysis of Diagnostic Test Accuracy.
2. Evidence in improving outcomes in renal diseases.
Workshops:
1. Systematic Reviews and Meta Analysis of Diagnostic Test Accuracy.
2. Evidenced informed care in renal diseases.
Key Note Address:
The Cochrane Collaboration: The way forward.

12th International Conference on Dialysis; International; United States
Role: Invited Speaker; **Year:** 2010
Conference Presentation Summary: Invited to present Evidence based nephrology – what has been achieved up to now.

Best Care for Australians with Chronic Kidney Diseases – the pathway ahead conference (KHA); National; Australia
Role: Invited Speaker; **Year:** 2009
Conference Presentation Summary: Patient perspectives on best care – lessons for the system

The Cochrane Colloquium; International; Singapore
Role: Invited Speaker; **Year:** 2009
Conference Presentation Summary: A lecture titled The Way Forward

(CIB) David Johnson
National Kidney Foundation Spring Clinical Meeting; International; United States
Role: Keynote Speaker; **Year:** 2014
Conference Presentation Summary: “New peritoneal dialysis solutions”

Australian and New Zealand Peritoneal Dialysis Academy; International; Australia
Role: Invited Speaker; **Year:** 2014
Conference Presentation Summary: "Infection management in peritoneal dialysis"

International Society of Nephrology Nexus Symposium Webinar; International; Australia
Role: Keynote Speaker; **Year:** 2014
Conference Presentation Summary: "Secondary outcomes from the IDEAL Trial"

American Society of Nephrology Annual Sicentific Meeting; International; United States
Role: Keynote Speaker; **Year:** 2014
Conference Presentation Summary: "High bicarbonate solutions"

National Kidney Foundation Spring Clinical Meeting; International; United States
Role: Keynote Speaker; **Year:** 2014
Conference Presentation Summary: "Working to decrease peritonitis rates: focus on training"

ANZ Peritoneal Dialysis Academy; International; Australia
Role: Invited Speaker; **Year:** 2013
Conference Presentation Summary: "Infection management in peritoneal dialysis"

14-15 March 2013

Australian and New Zealand Society of Nephrology; International; Australia
Role: Invited Speaker; **Year:** 2013
Conference Presentation Summary: "What's new and controversial in peritoneal dialysis"

7-9 September

ANZ Peritoneal Dialysis Academy; International; Australia
Role: Invited Speaker; **Year:** 2013
Conference Presentation Summary: "Peritoneal dialysis in Australia and New Zealand"

14-15 March 2013

Australian Diabetes Forum; National; Australia
Role: Invited Speaker; **Year:** 2013
Conference Presentation Summary: "Elevated uric acid in diabetic kidney disease,"

Amgen Nephrology Educational Symposium; International; Germany
Role: Invited Speaker; **Year:** 2013
Conference Presentation Summary: "Is there an IDEAL time to start dialysis?"

22-24 November, 2013.

World Congress of Nephrology; International; Hong Kong
Role: Invited Speaker; **Year:** 2013
Conference Presentation Summary: "Urate trials in chronic kidney disease"

World Congress of Nephrology; International; Hong Kong
Role: Invited Speaker; **Year:** 2013
Conference Presentation Summary: "Honey versus nasal mupirocin prophylaxis in peritoneal dialysis patients – the HONEYPOT trial"

May 30 – June 3, 2013

Annual Meeting of the International Society of Blood Purification; International; Italy
Role: Invited Speaker; **Year:** 2013
Conference Presentation Summary: "Innovation in Dialysis"

12-14 September, 2013.

EuroPD; International; Netherlands
Role: Invited Speaker; **Year:** 2013
Conference Presentation Summary: "Does the use of neutral pH, low glucose degradation product peritoneal dialysis fluids lead to better patient outcomes?"

11-14 October, 2013.

EuroPD; International; Netherlands
Role: Invited Speaker; **Year:** 2013
Conference Presentation Summary: "Biocompatibility – an update from the bedside,"

11-14 October, 2013.

EuroPD; International; Netherlands
Role: Invited Speaker; **Year:** 2013
Conference Presentation Summary: "The balANZ trial,"

11-14 October, 2013.

EuroPD; International; Netherlands
Role: Invited Speaker; **Year:** 2013

Conference Presentation Summary: “How to set up and run a clinical trial”

11-14 October, 2013

American Society of Nephrology; International; United States
Role: Invited Speaker; **Year:** 2013
Conference Presentation Summary: “Innovations in peritoneal dialysis”

November 5-10, 2013.

American Society of Nephrology; International; United States
Role: Invited Speaker; **Year:** 2013
Conference Presentation Summary: “Peritoneal dialysis: Update on Survival”

November 5-10, 2013

Australian and New Zealand Peritoneal Dialysis Academy; International; Australia
Role: Invited Speaker; **Year:** 2012
Conference Presentation Summary: “Peritonitis in Australia and New Zealand”

Sydney, 7-8 March 2012.

Home Dialysis Symposium; National; Australia
Role: Invited Speaker; **Year:** 2012
Conference Presentation Summary: “balANZ trial”

8-10 March 2012

Australian and New Zealand Peritoneal Dialysis Academy; International; Australia
Role: Invited Speaker; **Year:** 2012
Conference Presentation Summary: “Peritoneal dialysis-associated infection workshop”

Sydney, 7-8 March 2012.

City Wide Rounds; International; Canada
Role: Invited Speaker; **Year:** 2012
Conference Presentation Summary: “Peritonitis in Australia”

Toronto, Canada, 17 October 2012.

Ontario Renal Network Opening Plenary Lecture; International; Canada
Role: Keynote Speaker; **Year:** 2012
Conference Presentation Summary: “The role of biocompatible fluids in peritoneal dialysis”

Toronto, Canada, 12-13 October 2012.

European Renal Association – European Dialysis and Transplant Association Meeting; International; France
Role: Invited Speaker; **Year:** 2012
Conference Presentation Summary: “Glucose sparing peritoneal dialysis regimens”

Paris, 24-27 May 2012.

International Society of Peritoneal Dialysis Congress; International; Malaysia
Role: Keynote Speaker; **Year:** 2012
Conference Presentation Summary: “Biocompatible PD solutions: Implications for clinical practice”

Kuala Lumpur, Malaysia, 9-12 September, 2012.

International Society of Peritoneal Dialysis Congress; International; Malaysia
Role: Keynote Speaker; **Year:** 2012
Conference Presentation Summary: “Collaborative research programs in peritoneal dialysis”

Kuala Lumpur, Malaysia, 9-12 September, 2012.

International Society of Peritoneal Dialysis Congress; International; Malaysia
Role: Invited Speaker; **Year:** 2012
Conference Presentation Summary: “Results of the balANZ trial”

Kuala Lumpur, Malaysia, 9-12 September, 2012.

International Society of Peritoneal Dialysis Congress; International; Malaysia
Role: Invited Speaker; **Year:** 2012
Conference Presentation Summary: “Debate: Biocompatible fluids have proven benefits for residual renal function, peritoneal membrane and survival”

Kuala Lumpur, Malaysia, 9-12 September, 2012.

Australasian Creatinine Consensus Meeting; International; Australia
Role: Keynote Speaker; **Year:** 2011
Conference Presentation Summary: “Reporting limits for eGFR”

PD Academy; National; Australia
Role: Keynote Speaker; **Year:** 2011
Conference Presentation Summary: “Management of PD peritonitis”

Australasian Creatinine Consensus Meeting; International; Australia
Role: Keynote Speaker; **Year:** 2011
Conference Presentation Summary: “Age-related decision points for serum creatinine”

PD Academy; National; Australia
Role: Keynote Speaker; **Year:** 2011
Conference Presentation Summary: “Peritoneal dialysis outcomes in Australia and New Zealand”

Dialysis Nephrology Transplant Workshop; National; Australia
Role: Keynote Speaker; **Year:** 2011
Conference Presentation Summary: “CARI Guidelines: Early CKD”

Australasian Proteinuria Consensus Meeting; International; Australia
Role: Keynote Speaker; **Year:** 2011
Conference Presentation Summary: “Population screening for chronic kidney disease”

Australasian Creatinine Consensus Meeting; International; Australia
Role: Keynote Speaker; **Year:** 2011
Conference Presentation Summary: "Age-related decision points for eGFR"

Australasian Heads of Renal Units Meeting; International; Australia
Role: Keynote Speaker; **Year:** 2011
Conference Presentation Summary: "What is CKD in 2011?"

International Society of Blood Purification; International; China
Role: Keynote Speaker; **Year:** 2011
Conference Presentation Summary: "Early versus late start dialysis: what does the trial tell us"

Peritoneal Dialysis University; International; Malaysia
Role: Keynote Speaker; **Year:** 2011
Conference Presentation Summary: "Continuous quality improvement in peritoneal dialysis"

Peritoneal Dialysis University; International; Malaysia
Role: Keynote Speaker; **Year:** 2011
Conference Presentation Summary: "Key performance indicators in peritoneal dialysis"

4-CMGH Meeting; International; Taiwan, Republic Of China
Role: Keynote Speaker; **Year:** 2011
Conference Presentation Summary: "Management of PD peritonitis"

4-CMGH Meeting; International; Taiwan, Republic Of China
Role: Keynote Speaker; **Year:** 2011
Conference Presentation Summary: "Reaching standards of care in peritoneal dialysis"

EuroPD; International; United Kingdom
Role: Keynote Speaker; **Year:** 2011
Conference Presentation Summary: “Peritonitis in the Antipodes”

Cardiff Renal Colloquium; International; United Kingdom
Role: Keynote Speaker; **Year:** 2011
Conference Presentation Summary: “Exploiting patient cohorts to define infection patterns and outcomes: a tale from down under”

EuroPD; International; United Kingdom
Role: Keynote Speaker; **Year:** 2011
Conference Presentation Summary: “The balANZ trial results”

Improving Chronic Kidney Disease Outcomes; National; Australia
Role: Keynote Speaker; **Year:** 2010
Conference Presentation Summary: The Australasian Kidney Trials Network: empowering multidisciplinary research

University of Queensland Centenary Celebrations: Stellar Researchers Session; National; Australia
Role: Invited Speaker; **Year:** 2010
Conference Presentation Summary: Investigator-initiated trials in Nephrology: The agony and the ecstasy

Australian Association of Clinical Biochemists Annual Scientific Meeting; National; Australia
Role: Keynote Speaker; **Year:** 2010
Conference Presentation Summary: Global proteinuria guidelines

Mackay General practice Division Symposium; National; Australia
Role: Keynote Speaker; **Year:** 2010
Conference Presentation Summary: Management of Resistant Hypertension

Australasian Creatinine Consensus Meeting; International; Australia
Role: Keynote Speaker; **Year:** 2010
Conference Presentation Summary: Revised eGFR classification and its implications

Statewide Renal Clinical Network Forum; National; Australia
Role: Keynote Speaker; **Year:** 2010
Conference Presentation Summary: Queensland Statewide Renal Clinical Network Activity

Kidney School Australia; National; Australia
Role: Keynote Speaker; **Year:** 2010
Conference Presentation Summary: PD in Australia and New Zealand: A call to action

Improving Chronic Kidney Disease Outcomes; National; Australia
Role: Keynote Speaker; **Year:** 2010
Conference Presentation Summary: The IDEAL trial

International Perspectives in Chronic Kidney Disease Care; International; Australia
Role: Keynote Speaker; **Year:** 2010
Conference Presentation Summary: Management of CKD in Primary Care

International Perspectives in Chronic Kidney Disease Care; International; Australia
Role: Keynote Speaker; **Year:** 2010
Conference Presentation Summary: Industry and Clinical Trials

Home Dialysis Symposium; International; Australia
Role: Keynote Speaker; **Year:** 2010
Conference Presentation Summary: Preventing infections in peritoneal dialysis

Queensland Kidney Club; National; Australia
Role: Keynote Speaker; **Year:** 2010
Conference Presentation Summary: "PD in Australia and New Zealand: A call to action"

Home Dialysis Symposium; International; Australia
Role: Conference Committee; **Year:** 2010
Conference Presentation Summary: "All PD patients should be transferred to haemodialysis when they lose residual renal function: The case against"

Malaysian Society of Nephrology Annual Scientific Meeting; International; Malaysia
Role: Keynote Speaker; **Year:** 2010
Conference Presentation Summary: Improving standards of care in peritoneal dialysis

Malaysian Society of Nephrology Annual Scientific Meeting; International; Malaysia
Role: Keynote Speaker; **Year:** 2010
Conference Presentation Summary: Reducing drop-outs in peritoneal dialysis

International Society of Peritoneal Dialysis Congress; International; Mexico
Role: Keynote Speaker; **Year:** 2010
Conference Presentation Summary: Dialysis modality comparisons: The Australia and New Zealand experience

International Society of Peritoneal Dialysis Congress; International; Mexico
Role: Keynote Speaker; **Year:** 2010
Conference Presentation Summary: Achieiving continuous quality improvement in peritoneal dialysis

Asia-Pacific Renal Advisory Board; International; Singapore
Role: Keynote Speaker; **Year:** 2010
Conference Presentation Summary: Monitoring key performance indicators in peritoneal dialysis

Renal Fitness Program; International; Singapore
Role: Plenary Speaker; **Year:** 2010
Conference Presentation Summary: Reaching standards of care in peritoneal dialysis

Practical Evidence-Baed Insights in General Practice Summit; International; Viet Nam
Role: Keynote Speaker; **Year:** 2010
Conference Presentation Summary: Emerging strategies for Management of CV risk factors in Hypertension

Home Dialysis Symposium; International; Australia
Role: Plenary Speaker; **Year:** 2009
Conference Presentation Summary: Improving outcomes in peritoneal dialysis

National Chronic Kidney Disease Summit; National; Australia
Role: Presenter; **Year:** 2009
Conference Presentation Summary: Queensland Statewide Renal Clinical Network Activity

Roche National Nephrology and Transplant Symposium; National; Australia
Role: Keynote Speaker; **Year:** 2009
Conference Presentation Summary: The current peritoneal dialysis scene in Australia and New Zealand

General Practitioners' Continuing Education Conference; National; Australia
Role: Plenary Speaker; **Year:** 2009
Conference Presentation Summary: Management of stage 3 chronic kidney disease

Improving Peritoneal Dialysis Outcomes Symposium; National; Australia
Role: Keynote Speaker; **Year:** 2009
Conference Presentation Summary: PD Outcomes in Australia and New Zealand

Home Dialysis Symposium; International; Australia
Role: Keynote Speaker; **Year:** 2009
Conference Presentation Summary: “Planning for dialysis”

Peritoneal Dialysis Meeting; International; China
Role: Keynote Speaker; **Year:** 2009
Conference Presentation Summary: APD: The case against

Euro-PD; International; France
Role: Plenary Speaker; **Year:** 2009
Conference Presentation Summary: PD for all: Lessons from the ANZDATA Registry

World Congress of Nephrology; International; Italy
Role: Keynote Speaker; **Year:** 2009
Conference Presentation Summary: APD versus CAPD

Peritoneal Dialysis College; International; Korea, Republic Of
Role: Plenary Speaker; **Year:** 2009
Conference Presentation Summary: Metabolic syndrome in dialysis

Asia-Pacific RAAS Forum; International; Taiwan, Republic Of China
Role: Keynote Speaker; **Year:** 2009
Conference Presentation Summary: Diabetic nephropathy: benefits of blocking the RAAS

(CIC) Jeremy Chapman
WTC; International; United States
Role: Conference Committee; **Year:** 2014
Conference Presentation Summary: Finance Committee

Terasaki Feschtrift. Los Angeles. Invited presenter.; International; United States
Role: Invited Speaker; **Year:** 2014
Conference Presentation Summary: Terasaki Feschtrift. Los Angeles. Invited presenter.

ISODP. Sydney. Meeting President.; National; Australia
Role: Conference Convenor; **Year:** 2013
Conference Presentation Summary: ISODP. Sydney. Meeting President.

TSANZ. Canberra. Invited speaker.; National; Australia
Role: Invited Speaker; **Year:** 2013
Conference Presentation Summary: TSANZ. Canberra. Invited speaker.

1ST China-Australia Tx Xiangya-Westmead Summit Forum. Changsha. Invited speaker.; International; China
Role: Invited Speaker; **Year:** 2013
Conference Presentation Summary: 1ST China-Australia Tx Xiangya-Westmead Summit Forum. Changsha. Invited speaker.

2013 Changsha Summit Forum on International DCD Transplantation in China. Changsha. Invited speaker.; International; China
Role: Invited Speaker; **Year:** 2013
Conference Presentation Summary: 2013 Changsha Summit Forum on International DCD Transplantation in China. Changsha. Invited speaker.

WHO. Fiji. Public consultation forum – Organ & Tissue Transplantation Law.; International; Fiji
Role: Conference Committee; **Year:** 2013
Conference Presentation Summary: WHO. Fiji. Public consultation forum – Organ & Tissue Transplantation Law.

World Health Organisation. Fiji. Advisor to WHO Fiji Legislative framework for Organ & Tissue Transplantation in Fiji; International; Fiji
Role: Panel Member; **Year:** 2013
Conference Presentation Summary: World Health Organisation. Fiji. Advisor to WHO Fiji Legislative framework for Organ & Tissue Transplantation in Fiji

Seminar of Raising Awareness for Police and Customs Officials on Illegal and Fraudulent Activities (IFA) related to Organs, Tissues and Cells (OTC) – Issy Les Moulineaux. Invited Presenter; International; France
Role: Invited Speaker; **Year:** 2013
Conference Presentation Summary: Seminar of Raising Awareness for Police and Customs Officials on Illegal and Fraudulent Activities (IFA) related to Organs, Tissues and Cells (OTC) – Issy Les Moulineaux. Invited Presenter

Hong Kong Society of Transplantation. Hong Kong. Invited speaker.; International; Hong Kong
Role: Invited Speaker; **Year:** 2013
Conference Presentation Summary: Hong Kong Society of Transplantation. Hong Kong. Invited speaker.

Hong Kong Society of Nephrology. Hong Kong. Invited speaker.; International; Hong Kong
Role: Invited Speaker; **Year:** 2013
Conference Presentation Summary: Hong Kong Society of Nephrology. Hong Kong. Invited speaker.

Indian Society of Organ Transplantation. Bhubaneswar, India. Invited speaker; International; India
Role: Invited Speaker; **Year:** 2013
Conference Presentation Summary: Indian Society of Organ Transplantation. Bhubaneswar, India. Invited speaker

APEX. Mumbai. Guest speaker.; International; India
Role: Keynote Speaker; **Year:** 2013
Conference Presentation Summary: APEX. Mumbai. Guest speaker.

CAST. Kyoto, Japan. Invited speaker; International; Japan
Role: Invited Speaker; **Year:** 2013
Conference Presentation Summary: CAST. Kyoto, Japan. Invited speaker

XVI International Symposium on Renal Transplantation. Aguascalientes, Mexico. Invited speaker.; International; Mexico

Role: Invited Speaker; **Year:** 2013
Conference Presentation Summary: XVI International Symposium on Renal Transplantation. Aguascalientes, Mexico. Invited speaker.

SIUT. Karachi. Guest speaker; International; Pakistan
Role: Keynote Speaker; **Year:** 2013
Conference Presentation Summary: SIUT. Karachi. Guest speaker

The 5th Anniversary of the Declaration of Istanbul and a celebration of the Doha Donation Accord – Doha.; International; Qatar
Role: Panel Chair; **Year:** 2013
Conference Presentation Summary: The 5th Anniversary of the Declaration of Istanbul and a celebration of the Doha Donation Accord – Doha.

SICOT. Riaydh. Invited Speaker; International; Saudi Arabia
Role: Invited Speaker; **Year:** 2013
Conference Presentation Summary: March 2013

SATS Congress. Durban. Invited speaker.; International; South Africa
Role: Invited Speaker; **Year:** 2013
Conference Presentation Summary: SATS Congress. Durban. Invited speaker.

GAT Workshop. Durban. Invited presenter; International; South Africa
Role: Invited Speaker; **Year:** 2013
Conference Presentation Summary: GAT Workshop. Durban. Invited presenter

Liver Transplant Congress. Turkey. Invited speaker.; International; Turkey
Role: Invited Speaker; **Year:** 2013
Conference Presentation Summary: Liver Transplant Congress. Turkey. Invited speaker.

4th International Conference on Transplantomics and Biomarkers in Organ Transplantation. Cambridge. Meeting President; International; United Kingdom

Role: Conference Convenor; **Year:** 2013
Conference Presentation Summary: Transplantomics and Biomarkers in Organ Transplantation. Meeting President

Thomas Starlz Transplantation Institute Ground Rounds. Pittsburgh. Guest Presenter.; International; United States
Role: Keynote Speaker; **Year:** 2013
Conference Presentation Summary: Thomas Starlz Transplantation Institute Ground Rounds. Pittsburgh. Guest Presenter.

9th International Donor Registry Conference & WMDA Working Group. Sydney; National; Australia
Role: Invited Speaker; **Year:** 2012
Conference Presentation Summary: May 2012

TSANZ. Canberra; National; Australia
Role: Invited Speaker; **Year:** 2012
Conference Presentation Summary: June 2012

Canadian Transplant Forum. Toronto. Invited Speaker; International; Canada
Role: Invited Speaker; **Year:** 2012
Conference Presentation Summary: December 2012

Symposium. Developing Systems for Organ Transplantation Beijing. Invited Speaker; International; China
Role: Invited Speaker; **Year:** 2012
Conference Presentation Summary: October 2012

China-Australia Donor Transplant Summit. Changsha. Invited Speaker; International; China
Role: Invited Speaker; **Year:** 2012
Conference Presentation Summary: october 2012

Asian Colloquium Meeting. Seoul and Shanghai. Speaker; International; China

Role: Invited Speaker; **Year:** 2012
Conference Presentation Summary: speaker

TTS Congress. Berlin. Speaker Postgraduate workshop; International; Germany
Role: Invited Speaker; **Year:** 2012
Conference Presentation Summary: July 2012

ISOT. Mumbai. Invited Speaker; International; India
Role: Invited Speaker; **Year:** 2012
Conference Presentation Summary: October 2012

Second Global Consultative Meeting of Bologna Initiative for Global Vigilance & Surveillance Project. Rome. Australian representative; International; Italy
Role: Panel Member; **Year:** 2012
Conference Presentation Summary: November 2012

ICCA Client Workshop. Sarawak. Guest Speaker; International; Malaysia
Role: Invited Speaker; **Year:** 2012
Conference Presentation Summary: July 2012

Spanish Society of Transplantation. Madrid. Invited Speaker; International; Spain
Role: Invited Speaker; **Year:** 2012
Conference Presentation Summary: June 2012

ICCBBA WHO Retreat. Geneva. Australian Representative; International; Switzerland
Role: Panel Member; **Year:** 2012
Conference Presentation Summary: August 2012

Pfizer Forum. Taipei. Invited Speaker; International; Taiwan, Republic Of China
Role: Invited Speaker; **Year:** 2012
Conference Presentation Summary: August 2012

Thai Transplantation Society. Bangkok. Speaker; International; Thailand
Role: Invited Speaker; **Year:** 2012
Conference Presentation Summary: March 2012, speaker

Pfizer 12th International Transplant Symposium. Istanbul Invited Speaker.; International; Turkey
Role: Invited Speaker; **Year:** 2012
Conference Presentation Summary: April 2012, invited speaker

University of Minnesota. Minneapolis Guest Speaker.; International; United States
Role: Invited Speaker; **Year:** 2012
Conference Presentation Summary: November 2012

Transplantomics. San Diego. Meeting President; International; United States
Role: Conference Committee; **Year:** 2012
Conference Presentation Summary: March 2012, meeting President

ATC. Boston. Speaker; International; United States
Role: Invited Speaker; **Year:** 2012
Conference Presentation Summary: June 2012

ASN. San Diego. Invited Speaker; International; United States
Role: Invited Speaker; **Year:** 2012
Conference Presentation Summary: November 2012

ISODP; International; Argentina
Role: Invited Speaker; **Year:** 2011
Conference Presentation Summary:

TSANZ Annual Meeting; National; Australia
Role: Invited Speaker; **Year:** 2011
Conference Presentation Summary:

World Congress of Nephrology; International; Canada
Role: Invited Speaker; **Year:** 2011
Conference Presentation Summary:

XXI Latin American Congress and Pfizer Speaker Tour; International; Colombia
Role: ; **Year:** 2011
Conference Presentation Summary:

Renal Transplant Summit; International; India
Role: Invited Speaker; **Year:** 2011
Conference Presentation Summary:

Renal Transplant Summit; International; India
Role: Invited Speaker; **Year:** 2011
Conference Presentation Summary:

TTS nKOL; International; India
Role: ; **Year:** 2011
Conference Presentation Summary:

Transplantation Workshop.; International; Indonesia
Role: Invited Speaker; **Year:** 2011
Conference Presentation Summary:

Novartis Lecture Series; National; Israel
Role: Invited Speaker; **Year:** 2011
Conference Presentation Summary:

WHO NOTIFY Meeting; International; Italy
Role: ; **Year:** 2011
Conference Presentation Summary:

44th Meeting of the Japanese Society for Clinical Transplantation; International; Japan
Role: Invited Speaker; **Year:** 2011
Conference Presentation Summary:

12th CAST; International; Korea, Republic Of
Role: Invited Speaker; **Year:** 2011
Conference Presentation Summary:

Asia Pacific Summit; International; Korea, Republic Of
Role: Invited Speaker; **Year:** 2011
Conference Presentation Summary:

27th Annual Congress of the Malaysian Society of Nephrology; International; Malaysia
Role: ; **Year:** 2011
Conference Presentation Summary:

Philippine Society of Nephrology; International; Philippines
Role: Invited Speaker; **Year:** 2011
Conference Presentation Summary:

SGH 19th Annual Scientific Meeting; International; Singapore
Role: Invited Speaker; **Year:** 2011
Conference Presentation Summary:

Pfizer 11th International Transplant Symposium; International; Spain
Role: Invited Speaker; **Year:** 2011
Conference Presentation Summary:

Catalan Transplant Society; International; Spain
Role: Invited Speaker; **Year:** 2011
Conference Presentation Summary:

Transplantomics; International; Spain
Role: Conference Committee; **Year:** 2011
Conference Presentation Summary:

TTS/WHO Review-Symposium on Tx Ethics in Sri Lanka; International; Sri Lanka
Role: Invited Speaker; **Year:** 2011
Conference Presentation Summary:

WHO, First Global Consultative Meeting for BIG V&S Project; International; Switzerland
Role: Panel Member; **Year:** 2011
Conference Presentation Summary:

Novartis and Thailand Transplantion Society; International; Thailand
Role: Invited Speaker; **Year:** 2011
Conference Presentation Summary:

Pfizer Sponsored Symposium at ESOT; International; United Kingdom
Role: Invited Speaker; **Year:** 2011
Conference Presentation Summary:

American Society of Nephrology; International; United States
Role: Invited Speaker; **Year:** 2011
Conference Presentation Summary:

National Kidney Foundation; International; United States
Role: Panel Chair; **Year:** 2011
Conference Presentation Summary:

University of Minnesota Transplant Conference; National; United States
Role: Invited Speaker; **Year:** 2011
Conference Presentation Summary:

Vancouver - President XXII International Congress of The Transplantation Society; International; Canada
Role: Conference Committee; **Year:** 2010
Conference Presentation Summary: President

Shenzhen - Asia Pacific Colloquium on Organ Tranpslantation; International; China
Role: Invited Speaker; **Year:** 2010
Conference Presentation Summary: Invited Guest Speaker

Cairo - Declaration of Istanbul Custodian Group; International; Egypt
Role: Panel Member; **Year:** 2010
Conference Presentation Summary:

Mumbai - Apex Summit; International; India
Role: Invited Speaker; **Year:** 2010
Conference Presentation Summary:

Sorrento - 10th Pfizer International Transplantation Symposium; International; Italy
Role: Invited Speaker; **Year:** 2010
Conference Presentation Summary:

Seoul - Asia Pacific Colloquium on Organ Transplantation; International; Korea, Republic Of
Role: Session Chair; **Year:** 2010
Conference Presentation Summary:

Manila - Novartis Symposium; International; Philippines
Role: Invited Speaker; **Year:** 2010
Conference Presentation Summary:

Madrid - European Conference; International; Spain
Role: Invited Speaker; **Year:** 2010
Conference Presentation Summary:

Madrid - World Health Organisation Global Consultation; International; Spain
Role: Conference Committee; **Year:** 2010
Conference Presentation Summary:

Zurich - Ascertain Publication Meeting; International; Switzerland
Role: Conference Committee; **Year:** 2010
Conference Presentation Summary:

San Deigo - AKIN Summit; International; United States
Role: Conference Committee; **Year:** 2010
Conference Presentation Summary:

San Francisco - Transplantomics; International; United States
Role: Conference Committee; **Year:** 2010
Conference Presentation Summary:

San Diego - American Congress of Transplantation; International; United States
Role: Invited Speaker; **Year:** 2010
Conference Presentation Summary:

Buenos Aires - Argentinean Congress of Transplantation; International; Argentina
Role: Invited Speaker; **Year:** 2009
Conference Presentation Summary:

Vina del Mar, Chile - STALYC; International; Chile
Role: Invited Speaker; **Year:** 2009
Conference Presentation Summary:

Aswan - ASNRT Nephrology & Tx Congress; International; Egypt
Role: Invited Speaker; **Year:** 2009
Conference Presentation Summary:

Berlin- ISODP; International; Germany
Role: Invited Speaker; **Year:** 2009
Conference Presentation Summary:

Mumbai - Bone Marrow Transplantation; International; India
Role: Conference Committee; **Year:** 2009
Conference Presentation Summary:

Agra - nKOL; International; India
Role: Conference Committee; **Year:** 2009
Conference Presentation Summary: Meeting President

Venice - IXA & IPITA; International; Italy
Role: Invited Speaker; **Year:** 2009
Conference Presentation Summary:

Tokyo - Japanese Transplantation Society; International; Japan
Role: Invited Speaker; **Year:** 2009
Conference Presentation Summary: Invited Speaker, Medal Awards

Tokyo - Japanese Transplantation Society; International; Japan
Role: Invited Speaker; **Year:** 2009
Conference Presentation Summary: Invited Speaker, Medal Awards.

Kuala Lumpur - World Health Organisation Regional Meeting; International; Malaysia
Role: Conference Committee; **Year:** 2009
Conference Presentation Summary: TTS representative

Krakow - 3rd World Union of Tissue Bank Associations; International; Poland
Role: Keynote Speaker; **Year:** 2009
Conference Presentation Summary:

Riyadh - Saudi Society of Nephrology; International; Saudi Arabia
Role: Invited Speaker; **Year:** 2009
Conference Presentation Summary:

Geneva- Steering Group, Data Harmonization in Transplantation; International; Switzerland
Role: Conference Committee; **Year:** 2009
Conference Presentation Summary:

Geneva - World Health Assembly; International; Switzerland
Role: Conference Committee; **Year:** 2009
Conference Presentation Summary: Representative NEO in Official Relations

Geneva - World Health Organisation Executive Board; International; Switzerland
Role: Conference Committee; **Year:** 2009
Conference Presentation Summary:

Bangkok - Thai Transplantation Society; International; Thailand
Role: Invited Speaker; **Year:** 2009
Conference Presentation Summary:

(CID) Carmel Hawley

Queensland Nurses Education Update; National; Australia
Role: Invited Speaker; **Year:** 2014
Conference Presentation Summary: intradialytic hypotension

NSW Nephrologists state meeting; National; Australia
Role: Invited Speaker; **Year:** 2014
Conference Presentation Summary: AKTN: an important resource for the renal community

Biennial Home Dialysis Conference; National; Australia
Role: Invited Speaker; **Year:** 2014
Conference Presentation Summary: Recent home dialysis trials

Fresenius Medical Care, Annual Clinical Advisory Board Meeting; National; Australia
Role: Invited Speaker; **Year:** 2014
Conference Presentation Summary: Lowering dialysate sodium : proposal of cluster trial

Diamantina Health Partners Forum – Meeting of the Minds; National; Australia
Role: Invited Speaker; **Year:** 2014
Conference Presentation Summary: The Pursuit of Evidence : What can be done

Fresenius Medical Care, Annual Clinical Advisory Board Meeting; National; Australia
Role: Invited Speaker; **Year:** 2014
Conference Presentation Summary: Opportunity for trial utilising Adlosterone antagonism in chronic kidney disease

International Society of Nephrology Nexus Meeting; International; Italy
Role: Invited Speaker; **Year:** 2014
Conference Presentation Summary: Trials in Nephrology. Bridge of Clinical data across races

Amgen and Fresenius sponsored Nephrology Registrar Training Program; National; Australia
Role: Invited Speaker; **Year:** 2013
Conference Presentation Summary: Evidence-based medicine update – critical analysis of clinical trials and meta-analyses

Shire Clinical Insights meeting,; National; Australia
Role: Invited Speaker; **Year:** 2013
Conference Presentation Summary: News stories in nephrology: clinical trials last year in review

Shire CKD-MBD meeting; National; Australia
Role: Conference Committee; **Year:** 2013
Conference Presentation Summary: N/A

Dialysis Nephrology Transplantation (DNT) Annual meeting; National; Australia
Role: Invited Speaker; **Year:** 2013
Conference Presentation Summary: The Future of your trials Network

Fresenius Medical Care Clinical Advisory Board Meeting,; National; Australia
Role: Invited Speaker; **Year:** 2013
Conference Presentation Summary: HDF – The Catalina Study – Review/ Discussion

Roche Nephrology and Transplant Symposium; National; Australia
Role: Invited Speaker; **Year:** 2012
Conference Presentation Summary: How best to use your nurse practitioner

Medical Journal of Australia - Trials Summit; National; Australia
Role: Invited Speaker; **Year:** 2012
Conference Presentation Summary: The Australasian Kidney Trials Network

Queensland Renal physicians, State meeting; National; Australia
Role: Invited Speaker; **Year:** 2012
Conference Presentation Summary: Bone disease in the post-transplant setting

Amgen Nephrology Scientific Symposium; National; Australia
Role: Invited Speaker; **Year:** 2012
Conference Presentation Summary: HDF negative trials; in the future or the past? What is the evidence?

Translational research Institute Annual Symposium, Meeting of the Minds; National; Australia
Role: Invited Speaker; **Year:** 2012
Conference Presentation Summary: Australasian Kidney Trials Network

Biennial Home Dialysis Meeting; National; Australia
Role: Panel Member; **Year:** 2012
Conference Presentation Summary: Home Dialysis

Shire CKD-MBD meeting; National; Australia
Role: Conference Committee; **Year:** 2012
Conference Presentation Summary: N/A

Shire CKD-MBD meeting; National; Australia
Role: Invited Speaker; **Year:** 2012
Conference Presentation Summary: CKD-MBD Clinical Trials in Review

American Society of Nephrology; International; United States
Role: Invited Speaker; **Year:** 2012
Conference Presentation Summary: Clinical Trials in Nephrology Summary

Australian and New Zealand Society of Nephrology Annual Scientific Meeting; National; Australia
Role: Speaker selected from abstract; **Year:** 2011
Conference Presentation Summary: The post hoc analysis of the ADVANCE study.

Dialysis Nephrology Transplantation (DNT) Annual meeting; National; Australia
Role: Panel Chair; **Year:** 2011
Conference Presentation Summary: Dialysis Consumer Consensus

Shire CKD-MBD meeting; National; Australia
Role: Conference Committee; **Year:** 2011
Conference Presentation Summary: N/A

Australian and New Zealand Society of Nephrology Annual Scientific Meeting; International; Australia
Role: Invited Speaker; **Year:** 2011
Conference Presentation Summary: The AKTN Trials and Triumphs

Dialysis Nephrology Transplantation (DNT) Annual meeting; National; Australia
Role: Invited Speaker; **Year:** 2011
Conference Presentation Summary: Progress and strategic directions of the AKTN

world Congress of Nephrology Satellite meeting; International; Canada
Role: Invited Speaker; **Year:** 2011
Conference Presentation Summary: Trial Networks in nephrology: experiences, challenges and successes.

American Society of Nephrology; International; United States
Role: Invited Speaker; **Year:** 2011
Conference Presentation Summary: Establishing a clinical trials network in Nephrology; The key to success

Renal Society of Australasia - Annual Scientific Meeting; International; Australia
Role: Keynote Speaker; **Year:** 2010
Conference Presentation Summary: Bone Mineral Metabolism – Emerging Concepts

Amgen Australia Inaugural CKD-MBD Symposium; National; Australia
Role: Invited Speaker; **Year:** 2010
Conference Presentation Summary: “Calciphylaxis: origins and innovations in treatment”.

Australia and New Zealand Society of Nephrology (ANZSN) Annual Scientific Meeting; International; Australia
Role: Speaker selected from abstract; **Year:** 2010
Conference Presentation Summary: Outcomes associated with extended hours haemodialysis

Fresenius Advisory Board Meeting; National; Australia
Role: Invited Speaker; **Year:** 2010
Conference Presentation Summary: Dialysate Potassium

Queensland Renal dieticians annual meeting; National; Australia
Role: Invited Speaker; **Year:** 2010
Conference Presentation Summary: Vascular calcification

Shire CKD-MBD meeting; National; Australia
Role: Conference Committee; **Year:** 2010
Conference Presentation Summary: N/A

Princess Alexandra Hospital Research Week; National; Australia
Role: Invited Speaker; **Year:** 2010
Conference Presentation Summary: "Changing Environment for Research: Grasping the emerging Challenges and Opportunities- what Next?”

Shire “CKD-BMD” Annual Workshop; National; Australia
Role: Invited Speaker; **Year:** 2010
Conference Presentation Summary: “Non-Calcium based binders should be first-line in managing hyperphosphataemia in CKD”

Third Australia and New Zealand Home Therapies Workshop.; International; Australia
Role: Conference Convenor; **Year:** 2010
Conference Presentation Summary: Outcomes in home extended hours haemodialysis

World Congress of Internal Medicine; National; Australia
Role: Speaker selected from abstract; **Year:** 2010
Conference Presentation Summary: “The evidence for calcium, phosphate, vitamin D and PTH to improve cardiovascular outcomes in chronic kidney disease”

XL VII European Renal Association-European Dialysis and Transplant Association Congress; International; Germany
Role: Speaker selected from abstract; **Year:** 2010
Conference Presentation Summary: "Intervention for regression of LVH in patients with CKD: A systematic review and meta-analysis.”

Asian-Pacific Congress of Nephrology; International; Korea, Democratic People's Republic Of
Role: Speaker selected from abstract; **Year:** 2010
Conference Presentation Summary: Vitamin D Independently Predicts Cardiovascular Fitness in Patients with Chronic Kidney Disease.

American Society of Nephrology Annual Scientific Meeting; International; United States
Role: Speaker selected from abstract; **Year:** 2010
Conference Presentation Summary: Low 1,25-Dihydroxyvitamin D is Associated with Vascular Calcification, but Not Vascular Structure or Function in Chronic Kidney Disease
Australian home dialysis uptakes vary markedly, state by state and within states.

Dialysis Home Delivered - A Forum for Home Dialysis; National; Australia
Role: Invited Speaker; **Year:** 2009
Conference Presentation Summary: Results of the Health Professionals’ Survey

Pfizer CV Forum 2011 Specialist Weekend Meeting.; National; Australia
Role: Invited Speaker; **Year:** 2009

Conference Presentation Summary: Major News Stories and their implications to practice

Queensland Transplant Symposium; National; Australia
Role: Invited Speaker; **Year:** 2009
Conference Presentation Summary: The Marginal Donor – have we gone too far

Australia & New Zealand Society of Nephrology, Dialysis Nephrology Transplant Workshop; International; Australia
Role: Keynote Speaker; **Year:** 2009
Conference Presentation Summary: Practical Issues and Barriers to Home Dialysis

Australia and New Zealand Society of Nephrology Postgraduate Education Meeting; International; Australia
Role: Keynote Speaker; **Year:** 2009
Conference Presentation Summary: Ultrapure Dialysate: Does it Matter and How to achieve it

Australia and New Zealand Society of Nephrology (ANZSN) Annual Scientific Meeting; International; Australia
Role: Invited Speaker; **Year:** 2009
Conference Presentation Summary: Update for the Australasian Kidney Trials Network

9th Annual Australia and New Zealand Society of Nephrology Postgraduate Meeting; International; Australia
Role: Keynote Speaker; **Year:** 2009
Conference Presentation Summary: HDF and new Dialysis Therapies

Queensland Renal Advanced Trainee Society Bi-Monthly Meeting; National; Australia
Role: Keynote Speaker; **Year:** 2009
Conference Presentation Summary: Haemodiafiltration : an Overview

Fresenius Medical Care.- Annual Advisory Board Meeting; National; Australia
Role: Invited Speaker; **Year:** 2009

Conference Presentation Summary: Ideal Body Weight Assessment

Annual Scientific Congress, Royal Australasian College of Surgeons; National; Australia
Role: Keynote Speaker; **Year:** 2009
Conference Presentation Summary: Pathogenesis of secondary hyperparathyroidism

Australia & New Zealand Society of Nephrology, Dialysis Nephrology Transplant Workshop; International; Australia
Role: Invited Speaker; **Year:** 2009
Conference Presentation Summary: Clinical Trials Update

Australian Research Collaboration Service (ARCS) Conference; National; Australia
Role: Speaker selected from abstract; **Year:** 2009
Conference Presentation Summary: "Building relationship in Clinical Research"

Cardiovascular Sympsoium; National; Australia
Role: Invited Speaker; **Year:** 2009
Conference Presentation Summary:

42nd Annual Meeting and Scientific Exposition of the American Society of Nephrology; International; United States
Role: Speaker selected from abstract; **Year:** 2009
Conference Presentation Summary: Extended hours hemodialysis in Australia.

(CIE) Stephen McDonald
World Transplant Congress; International; United States
Role: Speaker selected from abstract; **Year:** 2014
Conference Presentation Summary: Oral presentation from submitted abstract:Outcomes of kidneys from circulatory vs brain death donors

Health Roundtable ESKD meeting; National; Australia
Role: Invited Speaker; **Year:** 2014

Conference Presentation Summary: Invited speaker on trends in ESKD

Japanese Society of Dialysis Therapy Annual Scientific Meeting; International; Japan
Role: Invited Speaker; **Year:** 2014
Conference Presentation Summary: Invited to give presentation on contribution of Registry data to nephrology

World Transplant Congress; International; United States
Role: Invited Speaker; **Year:** 2014
Conference Presentation Summary: Invited speaker in pre-meeting symposium "Biostatistics 101" on "Descriptive data -- diamonds in the Rough"

Indigenous Kidney Transplant Outcomes; National; Australia
Role: Conference Convenor; **Year:** 2013
Conference Presentation Summary: Organiser of conference. Obtained funding, organised conferences

Transplantation Society of Australia & New Zealand; National; Australia
Role: Invited Speaker; **Year:** 2013
Conference Presentation Summary: Invited speaker about indigenous outcomes

Dialysis Nephrology Transplantation Workshop; National; Australia
Role: Invited Speaker; **Year:** 2013
Conference Presentation Summary: Summary of development oin centre-specific reporting

Indigenous Kidney Transplant Outcomes; National; Australia
Role: Invited Speaker; **Year:** 2013
Conference Presentation Summary: Speaker on tx outcomes overall

Clinical registries seminar: monitoring and improving health outcomes; National; Australia
Role: Invited Speaker; **Year:** 2013

Conference Presentation Summary: "Experiences from the Australia and New Zealand dialysis and transplant registry"

Account of the work done in recent years to drive change in reporting of unit-specific otucomes and implement a feedback loop

American Transplant Congress; International; United States
Role: Speaker selected from abstract; **Year:** 2013
Conference Presentation Summary: "The Time-Varying Relationship between Aboriginal Racial Origin and Transplant Outcomes in Australia."

Aboriginal Australians have markedly higher rates of kidney disease. Rates of kidney transplantation from deceased donor kidneys are substantially lower, with few live donors. Outcomes from transplantation in this group appear to be poorer but have not been well examined.

Utilising the ANZDATA Registry, Australian primary deceased-donor kidney-only transplants 1991-2011 were examined (n=6451). Time to graft failure (composite of death with graft function & loss of graft function) was examined piecewise due to non-proportional risks. Subhazards for death with function vs. loss of graft function were examined with competing risks methods.

Aboriginal recipients (n=327) were younger and more commonly female, smokers, diabetic, and to have vascular disease or chronic lung disease; at transplant they had higher waiting times, peak PRA and HLA mismatches

Risk of graft failure (covariates adjusted) varied over time after transplantation, peaking 2 years after transplantation, with no interaction with year of transplantation. [figure1] Adjusted subhazard ratios showed increased risk for death with graft function from 3 months post transplantation , whereas the rate of graft loss was not increased until 12 months post transplantation. [figure2] Among Aboriginal recipients, the excess death rate was predominantly due to infectious causes in the first year (65% vs 37%), and thereafter cardiovascular causes (57% vs 31% in 2nd year).

Although the outcomes among Aboriginal transplant recipients are poorer than non-Aboriginal recipients, the risk is not homogenous over time. Outcomes in the first 3 months are comparable; after this poorer graft outcomes reflect excess infective deaths in first year among Aboriginal recipients, with cardiovascular disease and increased loss of graft function later factors.

American Transplant Congress; International; United States
Role: Speaker selected from abstract; **Year:** 2013
Conference Presentation Summary: “The Occurrence of Non-Melanoma Skin Cancer Predicts Risk of Subsequent Solid Organ Cancer.”

Among kidney transplant recipients, there is an increased risk of solid organ cancer after the occurrence of a NMSC. This risk is independent of other risk factors for cancer such as age, year of transplantation (a proxy for immunosuppression), anti-CD3 use. Whether this relationship marks an underlying propensity to malignant transformation, or an increased vulnerability to the carcinogenic effects of immunosuppressive therapy is not clear.

Australia and New Zealand Home Therapies Conference; National; Australia
Role: Plenary Speaker; **Year:** 2012
Conference Presentation Summary: Summary of state of epidemiology of uptake of dialysis home therapies in Australia, with emphasis on geographic variation and comparison with other countries.

International Society of Renal Epidemiology; International; Australia
Role: Invited Speaker; **Year:** 2012
Conference Presentation Summary: Satellite meeting to WNC. Invited speaker on Aboriginal Renal disease

Japanese Society of Dialysis Therapy; International; Japan
Role: Plenary Speaker; **Year:** 2012
Conference Presentation Summary: Plenary presentation -- financial aspects of dialysis treatment in Australia

Dialysis Nephrology Transplant Workshop; National; Australia
Role: Invited Speaker; **Year:** 2011
Conference Presentation Summary: Invited speaker on dialysis KPIs

Annual Scientific Meeting, Australia & New Zealand Society of Nephrology; National; Australia
Role: Conference Convenor; **Year:** 2011
Conference Presentation Summary: Chair of local organising committee for 2011 ASM of ANZSN.

Renal Society of Australasia; National; Australia
Role: Plenary Speaker; **Year:** 2011
Conference Presentation Summary: Renal Disease in Australasia

Renal Disease in Indigenous and Disadvantaged Peoples; International; Canada
Role: Invited Speaker; **Year:** 2011
Conference Presentation Summary: Satellite conference to World Congress in Nephrology. Presentation "Kidney DIsease in Indigenous Australians"

International Society of Haemodialysis; International; India
Role: Invited Speaker; **Year:** 2011
Conference Presentation Summary: Dialysis Registries in Australia

Annusal Scientific Meeting, ANZN Society of Nephrology; ; Australia
Role: Plenary Speaker; **Year:** 2009
Conference Presentation Summary:

Annual Scientic Meeting, Transplantation Society of Australia; ; Australia
Role: Plenary Speaker; **Year:** 2009
Conference Presentation Summary:

Renal Disease in Disadvantaged populations; ; Italy
Role: Plenary Speaker; **Year:** 2009
Conference Presentation Summary:

CV-CE: Community Engagement (last 5 years only)

Team Member	Title/Topic	Form of Engagement	Audience	Representing Whom	Location	Frequency	Duration	Did this involve Aboriginal or Torres Strait Islander Peoples?
(CIA) Jonathan Craig	Research Priority Setting Partnership	Roundtable meeting	Clinicians, patients, policy makers, researchers	Research Collaboration	Sydney	Once	//2014 - //	No
	Commonwealth Indigenous Reform Group	Presentation and workshop	Departments of Prime Minister and Cabinet, Treasury, DoHA, Housing, Community Services etc	University of Sydney	Canberra. Department of Industry, Innovation, Science, Research and Tertiary Education	Once	//2013 - //	Yes
	SEARCH - CEOs forum	Meetings with CEOs of participating ACCHO	ACCHOs	Investigators	Various	Biannually	//2010 - //	Yes
(CIB) David Johnson	Member of the Australian Primary Healthcare Collaborative on Chronic Kidney Disease Expert Reference Panel	Provide advice regarding chronic kidney disease detection, management and prevention	General practitioners, Government	Kidney Health Australia	Improvement Foundation, Melbourne	Annually	//2014 - //	Yes
	Clinical InfoNet	Review patient and doctor educational materials for accuracy of content	Community, General Practitioners, Government, Indigenous Healthcare Workers, Practice Nurses	Kidney Heakth Australia	Perth, Western Australia	11+ times	//2012 - //	Yes

	Member of The Australian Primary Care Collaborative in Indigenous Healthcare: Bridging the Gap Expert Reference Panel	Provide advice regarding chronic kidney disease detection, management and prevention	Primary Care, Government	Kidney Health Australia	Improvement Foundation, Melbourne	Annually	//2010 - //	Yes
	Australian Primary Care Collaborative in Chronic Disease Prevention and Self-Management Expert Reference Panel	Provide advice regarding chronic kidney disease detection, management and prevention	Primary Care, Government	Kidney Health Australia	Improvement Foundation, Melbourne	Annually	//2009 - //	Yes
	Australian Primary Care Collaborative in Coronary Heart Disease Expert Reference Panel	Provide advice regarding chronic kidney disease detection, management and prevention	Primary Care, Government	Kidney Health Australia	Improvement Foundation, Melbourne	Annually	//2007 - //	Yes
	Australian Primary Care Collaborative in Diabetes Mellitus Expert Reference Panel	Provide advice regarding chronic kidney disease detection, management and prevention	Primary Care, Government	Kidney Health Australia	Improvement Foundation, Melbourne	Annually	//2007 - //	Yes
	National Chronic Kidney Disease Strategy and Policy Working Group		Consumers, Government	Kidney Health Australia		Quarterly	//2007 - //	No
	Media Spokesperson for Kidney Health Australia on Chronic kidney disease and Organ donation	Media Spokesperson for Kidney Health Australia	General public	Kidney Health Australia	Queensland	11+ times	/6/2005 - //	Yes
(CID) Carmel Hawley	Board Member, Kidney Health Australia	Member of the Board	Patients with Kidney Disease	Renal Physicians in Australia	Melbourne	Monthly	//2004 - //2009	No

	All Hallows School Finance Committee	Meetings and School interaction	Parents and Teachers	Parent Body	Brisbane	Monthly	//2001 - //2011	No
(CIE) Stephen McDonald	ANZ Society of Nephrology	Chair, Local organising committee, 2011 Annual Scientific Meeting	ANZSN Society of Nephrology		Adelaide		/5/2009 - /10/2011	No
	Clinical Governance Committee Membership, Country Health SA	Committe membership		SA dialysis units	Adelaide	Monthly	/10/2009 - //	No
	National Transplant Authority Clinical Information Systems Committee	Committee Membership		ANZDATA Registry		Monthly	/12/2009 - //	No

CV-SM: Supervision & Mentoring (last 5 years only)

Team Member	Supervised / Mentored	Start Year	Number of Students
(CIA) Jonathan Craig	Mentored - In a research environment - Postdoc	2014	8
	Supervised - In a research environment - PhD	2014	13
	Supervised - In a research environment - PhD	2013	15
	Mentored - In a research environment - Postdoc	2013	7
	Mentored - In a research environment - Postdoc	2012	7
	Supervised - In a research environment - PhD	2012	15
	Mentored - In a research environment - Postdoc	2011	7
	Supervised - In a research environment - PhD	2011	11
	Mentored - In a research environment - Postdoc	2010	7
	Supervised - In a research environment - PhD	2010	12
	Supervised - In a research environment - PhD	2009	12
	Mentored - In a research environment - Postdoc	2009	6
(CIB) David Johnson	Supervised - In a clinical environment - PhD	2013	5
	Supervised - In a clinical environment - PhD	2012	5
	Supervised - In a clinical environment - PhD	2011	4
	Supervised - In a research environment - PhD	2010	4
	Supervised - In a clinical environment - PhD	2010	3
	Mentored - In a clinical environment - PhD	2010	1
	Mentored - In a research environment - PhD	2010	1
	Supervised - In a clinical environment - PhD	2009	3
	Mentored - In a research environment - PhD	2009	7
(CIC) Jeremy Chapman	Mentored - In a research environment - Postdoc	2013	2
	Supervised - In a research environment - PhD	2013	2
	Mentored - In a research environment - Postdoc	2012	2
(CID) Carmel Hawley	Supervised - In a clinical environment - Masters	2014	1
	Supervised - In a clinical environment - PhD	2013	1

	Supervised - In a clinical environment - PhD	2012	1
	Supervised - In a clinical environment - PhD	2011	1
	Supervised - In a clinical environment - PhD	2010	1
	Mentored - In a clinical environment - PhD	2009	2
(CIE) Stephen McDonald	Supervised - In a clinical environment - PhD	2014	1
	Mentored - In a research environment - PhD	2012	1
	Mentored - In a research environment - Postdoc	2011	1
	Supervised - In a clinical environment - PhD	2009	1
	Mentored - In a clinical environment - Postdoc	2009	1

CV-RF: NHMRC Research Funding (last 5 years only)

Team Member & AppID	Title	Funding Type & Grant Type	Your Role	First Year Funded	No of Years	Total Amount ($AUD)	% P/W
(CIA) Jonathan Craig N/A	Cochrane Review Groups with their sole editorial base in Australia: Cochrane Renal Group.	Research Support, National Health Research Enabling Capabilities (NHREC)	CIA	2013	3	$360,000.00	5
457281	A Multidisciplinary,multi-method focus on kidney disease	Research Support, CAPACITY	CIA	2007	5	$2,616,629.00	0
631731	Australasian Kidney Trials (AKT) Network	Infrastructure / Equipment Support, Enabling	CIF	2010	6	$2,000,000.00	3
338800	Australasian Kidney Trials (AKT) Network	Research Support, Enabling	CIG	2005	5	$1,290,000.00	0
1035378	SEARCH: better evidence, better health for urban Aboriginal children	Research Support, Partnership Project	CIA	2012	3	$1,395,760.00	5
571447	Making clinical networks work better	Research Support, Partnership Project	CIE	2010	3	$338,850.00	2
633003	Screening and Test Evaluation Program	Research Support, Program Grant	CIC	2011	5	$9,084,385.00	80
1023998	SEARCH: Study of Environment on Aboriginal Resilience in Child Health	Research Support, Project	AI	2012	5	$1,727,460.00	5
1050583	Antecedents of Renal Disease in Aboriginal Children and Young Adults – 16 year follow-up.	Research Support, Project Grant	CIA	2013	5	$1,816,455.23	5
512248	ARDAC Second Phase Study	Research Support, Project Grant	CIA	2008	5	$1,825,649.00	0

N/A	Cochrane Renal Group Funding	Research Support, Targeted Research	CIA	2011	2	$202,500.00	0
358457	SEARCH - Study of environment on Aboriginal Resilience and child health	Research Support,	CIA	2006	5	$2,043,111.00	0
402764	Screening and Test Evaluation Program (STEP)	Research Support,	CIB	2006	5	$6,705,455.00	0
(CIB) David Johnson APP1061235	Improving peritoneal dialysis practices and outcomes in Australia	Research Support, Project	Chief Investigator	2014	3	$473,230.00	0
631731	Australasian Kidney Trials (AKT) Network	Infrastructure / Equipment Support, Enabling	CID	2010	6	$2,000,000.00	20
338800	The Australasian Kidney Trials Network (AKTN)	Infrastructure / Equipment Support, Enabling	CIC	2005	5	$1,290,000.00	10
APP1030584	Serial Nt-Probnp Monitoring For Predicting Major Cardiovascular Events In The Dialysis Population	Research Support, Project	CIA	2012	3	$281,193.00	10
APP1008604	The HERO Trial: Handling Erythropoietin Resistance with Oxpentifylline	Research Support, Project	CIA	2011	3	$333,409.00	10
APP1012009	Macrophages: a therapeutic target in peritoneal dialysis-induced fibrosis?	Research Support, Project	CIC	2011	3	$573,390.00	5
631576	Investigating how Epo therapy can be improved to provide best protection for heart and kidney after ischaemic injury	Research Support, Project	CIA	2010	3	$355,374.00	15
511109	Tailoring doses of the newer immunosuppressant drugs	Research Support, Project	CID	2008	3	$486,207.00	0

401568	Identification and characterisation of the way erythropoietin protects tissue from oxygen stress	Research Support, Project	CIA	2006	4	$477,661.00	0
APP1043203	Controlled trial of slowing of Kidney Disease progression From the Inhibition of Xanthine oxidase (CKD-FIX): A double-blind, randomised, placebo-controlled trial	Research Support, Project Grant	CIA	2013	5	$1,917,146.74	25
APP1042474	The SIGNAL trial	Research Support, Project Grant	CID	2013	5	$2,700,000.00	10
(CID) Carmel Hawley GNT1044302	IMPROVE-CKD Study: Impact of a phosphate binder on cardiovascular disease in Chronic Kidney Disease	Research Support, Project Grant	CIA	2013	5	$1,595,697.00	25
631731	Australasian Kidney Trials (AKT) Network	Infrastructure / Equipment Support, Enabling	CIA	2010	6	$2,000,000.00	30
338800	Australasian Kidney trials (AKT) Network	Infrastructure / Equipment Support, Enabling	CIA	2006	5	$1,290,000.00	0
1030584	Serial NT-proBNP Monitoring for Predicting Major Cardiovascular Events in the Dialysis Population	Research Support, Project Grant	Chief Investigator	2012	2	$281,193.00	0
1006171	BLOCADE Trial	Research Support, Project Grant	CIB	2011	3	$579,527.20	15
571045	ACTIVE Dialysis	Research Support, Project Grant	CIE	2009	4	$1,248,175.00	15
458652	A trial of aspirin and fish oil for the prevention of thrombosis in dialysis access	Research Support, Project Grant	CIE	2007	4	$1,793,000.00	15

(CIE) Stephen McDonald APP1061235	Improving peritoneal dialysis practices and outcomes in Australia	Research Support, Project	AI	2014	3	$473,230.00	0
457281	Improving population health by a multidisciplinary, multi-method focus kidney disease of the young, the old, and of Indigenous Australians	People Support, CAPACITY	CIE	2007	5	$2,595,487.00	5
APP1027156	Long-term implications of becoming a living kidney donor	Research Support, Project	CIE	2012	5	$259,332.50	10
APP1008604	A randomised, placebo controlled trial of Oxpentifylline versus placebo in the treatment of erythropoietin-resistant anaemia in chronic kidney disease	Research Support, Project Grant	CIE	2011	3	$333,409.00	5

CV-ORF: Other Research Funding (last 5 years only)

Team Member & App ID	Funding Organisation	Domestic/ International	Funding Source	Peer Reviewed	Your Role	First Year Funded	No of Years	Total Amount ($AUD)	% P/W
(CIA) Jonathan Craig N/A	Kidney Health Australia	Domestic	NGO's (non-profit)	Yes	Contribution to the design, conduct, publication and dissemination	2013	1	$45,000.00	2
N/A	The University of Sydney, International Program Development Fund	Domestic	University	Yes	Contribution to the design, conduct, publication and dissemination	2012	3	$18,040.00	2
N/A	APHCRI Centre of Research Excellence Grant	Domestic	Government	Yes	Chief Investigator	2012	4	$2,500,000.00	5
PG31 12	Kidney Health Australia Biomedical Project Grant	Domestic	NGO's (non-profit)	Yes	Co investigator	2012	1	$50,000.00	1
LP100100300	ARC Linkage Project	Domestic	Government	Yes	Chief Investigator	2012	3	$292,000.00	5
2011-224	Foundation for Children	Domestic	Philanthropic	Yes	Chief Investigator	2011	3	$163,920.00	0

NA	Therapeutic Innovation Australia (Translating Health Discovery Program)	Domestic	Government		Applicant for a data capture and management system for clinical trials	2011	2	$910,000.00	0
N/A	ANZSN Amgen Quality Assurance Grants Programme	Domestic	NGO's (non-profit)	Yes	Chief Investigator	2010	1	$10,000.00	0
N/A	Kidney Health Australia	Domestic	NGO's (non-profit)		Clinical and scientific leadership	2010	2	$10,000.00	1
N/A	Sydney Medical School	Domestic	University	Yes	Co investigator	2010	1	$30,000.00	1
N/A	Estate of the Elizabeth Littie May Rosenthal for Bone Research	Domestic	Philanthropic	Yes	Co invesitgator	2009	2	$100,000.00	2
2008-009	The Financial Markets Foundation for Children	Domestic	Philanthropic	Yes	Chief Investigator	2008	2	$134,684.00	0
N/A	University of Sydney Data Linkage project funding	Domestic	University	Yes	Co investigator	2008	3	$10,000.00	1

N/A	Agenzia Italiana del Farmaco (AIFA)-Italy (DOSE trial)	International	Other		Co investigator	2006	4	$3,039,713.00	0
(CIB) David Johnson N/A	Fresenius Medical Care Grant	International	Commercial	Yes	(CIA)	2014	1	$30,630.00	0
N/A	Princess Alexandra Hospital Research Infrastructure Support Grant	Domestic	NGO's (non-profit)	Yes	Recipient CIA	2014	1	$19,200.00	0
N/A	Baxter Clinical Evidence Council (CEC) Grant	International	Commercial	Yes	Recipient (CIA) - “Evaluation of the integrated home dialysis”	2014	3	$188,444.00	0
N/A	PA Research Support Scheme Small Grant	Domestic	Research Institution	Yes	CIA for topic “The utility of serum interleukin-6 level in peritoneal dialysis patients.”	2013	1	$20,000.00	4
(PGO6 13)	Kidney Health Australia	Domestic	NGO's (non-profit)	Yes	Chief Investigator	2013	1	$45,000.00	5
N/A	PA Research Support NHMRC Infrastructure Grant.	Domestic	Research Institution	Yes	Chief Investigator	2013	1	$27,200.00	0

RM 2013001202	UQ Academic Title Holder Research Fund Scheme 2013	Domestic	University	Yes	Recipient (CID)	2013	1	$10,000.00	
N/A	PA Research Support Scheme Grant	Domestic	Research Institution	Yes	CIA-A randomised, placebo-controlled trial of synbiotics in the management of chronic kidney disease	2013	1	$75,000.00	5
N/A	Fresenius Medical Care Research Grant	International	Commercial	Yes	Principal Investigator for balANZ Biomarker Study	2012	1	$45,000.00	5
N/A	Princess Alexandra Research Foundation	Domestic	Research Institution	No	Chief Investigator	2012	1	$17,900.00	0
PB-PG-0610-22456	National Institute of Health Research	International	Government	Yes	Chief Investigator	2012	4	$379,572.00	5
N/A	Kidney Health Australia	Domestic	NGO's (non-profit)	Yes	Chief Investigator	2011	2	$50,000.00	2
N/A	Kidney Health Australia	Domestic	NGO's (non-profit)	Yes	Chief Investigator	2011	2	$49,394.00	2
N/A	Princess Alexandra Research Foundation	Domestic	Research Institution	Yes	Chief Investigator	2011	1	$75,000.00	2

N/A	Princess Alexandra Research Foundation	Domestic	Research Institution	Yes	Chief Investigator	2011	1	$65,000.00	2
N/A	Novartis	International	Commercial	Yes	Recipient - Novartis Research Grant “Mycophenolate pharmacokinetics.”	2010	4	$237,000.00	0
N/A	Queensland Government Health Research Fellowship	Domestic	Government	Yes	Fellowship Recipient	2010	4	$750,000.00	10
N/A	Queensland Health - Health Practitioner Research Scheme	Domestic	Government	Yes	Chief Investigator	2010	1	$45,000.00	0
N/A	Clinical Practice Improvement Centre Clinical Service Improvement Funding	Domestic	Government	Yes	Principal Investigator	2008	3	$146,909.00	2
N/A	Clinical Practice Improvement Centre Clinical Service Improvement Funding	Domestic	Government	Yes	Chief Investigator	2008	3	$146,909.00	1

N/A	Baxter Clinical Evidence Council	International	Commercial	Yes	Chief Investigator	2008	3	$180,000.00	0
N/A	Queensland Health Smart Health Research Grants	Domestic	Government	Yes	Principal Investigator	2008	3	$280,000.00	4
N/A	Baxter Extramural Grant	International	Commercial	Yes	Principal Investigator	2008	3	$500,000.00	6
	Fresenius Medical Care Research Grant	International	Commercial	Yes	Principal Investigator in The balANZ randomized controlled trial	2004	8	$3,000,000.00	5
(CIC) Jeremy Chapman Nil	Juvenile Diabetes Research Foundation	Domestic	NGO's (non-profit)	Yes	Chief Investigator	2006	4	$10,029,261.00	10
(CID) Carmel Hawley N/A	PA Research Foundation	Domestic	Research Institution	Yes	CI - IMPROVE Trial	2013	3	$19,000.00	5
N/A	Amgen	Domestic	Commercial	No	CI - FAVOURED Trial	2012	2	$200,000.00	15
N/A	Abbott Laboratories	Domestic	Commercial	No	CI - FAVOURED Trial	2012	2	$200,000.00	15
N/A	Kidney Health Australia	Domestic	NGO's (non-profit)	Yes	Principal Investigator - Monitor	2011	2	$50,000.00	10
N/A	Princess Alexandra Research Foundation	Domestic	Research Institution	Yes	Principal Investigator - Monitor	2011	1	$75,000.00	10

N/A	Shire PLC	International	Commercial	No	Principal Investigator - IMPROVE Trial	2010	5	$1,500,000.00	25
N/A	Fresenius	Domestic	Commercial	No	CI - MONITOR trial equipment support	2010	4	$65,000.00	5
N/A	Roche foundation for Anaemia Research	Domestic	Commercial	Yes	AI - HERO trial	2008	2	$200,000.00	0
N/A	Queensland Health, Smart Health Research Grant	Domestic	Government	Yes	CI - HONEYPOT Trial	2008	3	$280,000.00	5
(CIE) Stephen McDonald n/a	Kidney Health Australia	Domestic	NGO's (non-profit)	No	Executive Officer, ANZDATA Registry	2014	1	$75,000.00	0
None	Australian Organ and Tissue Donation and Transplantation Authority	Domestic	Government	No	Executive Officer, ANZDATA Registry. Negotiated quantum and scope of funding.	2014	2	$1,676,600.00	10
n/a	NZ government Ministry of Health	International	Government	No	Executive Officer, ANZDATA Registry	2014	2	$274,945.00	0
n/a	Kidney Health Australia	Domestic	Philanthropic	Yes	AI	2013	2	$49,969.00	2
n/a	Australia and NZ Society of Nephrology	Domestic	NGO's (non-profit)	Yes	CIB	2013	1	$45,000.00	5
n/a	Kidney Health Australia Research Grants	Domestic	NGO's (non-profit)	Yes	Chief Investigator B	2012	1	$48,344.00	5

n/a	Australian Organ and Tissue Donation and Transplantation Authority	Domestic	Government	No	Executive Officer, ANZDATA Registry	2012	2	$1,295,140.00	10
n/a	Australian Organ and Tissue Donation and Transplantation Authority	Domestic	Government	No	Executive Officer, ANZDATA Registry	2011	1	$506,000.00	15
n/a	Australian Organ and Tissue Donation and Transplantation Authority	Domestic	Government	No	Executive Officer, ANZDATA Registry	2010	1	$383,943.00	10
n/a	NZ Ministry of Health (Pro--rata with Australian govt contribution)	International	Government	No	Executive Officer	2009	1	$69,530.00	2
n/a	Australian Organ Donation and Transplantation Authority	Domestic	Government	No	Executive Officer, ANZDATA Registry	2009	1	$295,183.00	10

CV-P: Patents

(CIA) Jonathan Craig

Patent: none: , **Status:**
Registered in names of (Inventors):
Description of patent:
Applicability/Impact:

CV-W: Workload (Current)

(CIA) Professor Jonathan Craig

Weekly Load (average hours/week)

Teaching	Clinical	NHMRC Research	Other Research
2	8	25	5

Administrative Responsibilities (average hours/week)

Administrative Commitment: 5
Responsibilities: Directing a Clinical Epidemiology program at the University of Sydney

(CIB) Professor David Johnson

Weekly Load (average hours/week)

Teaching	Clinical	NHMRC Research	Other Research
6	18	20	12

Administrative Responsibilities (average hours/week)

Administrative Commitment: 6
Responsibilities: Director of Nephrology, Princess Alexandra, Ipswich, Redlands & Logan Hospitals, Medical Director, Queensland Renal Transplant Service, Chair, Queensland Statewide Renal Clinical Network, Director, Centre for Kidney Disease Research, Deputy Chair, Executive Operations, Australasian Kidney Trials Network

(CIC) Professor Jeremy Chapman

Weekly Load (average hours/week)

Teaching	Clinical	NHMRC Research	Other Research
5	20	0	15

Administrative Responsibilities (average hours/week)

Administrative Commitment: 15
Responsibilities: Division of Medicine and Cancer,
Western Sydney Local Health District, Westmead Hospital, Clinical Professor, University of Sydney, Faculty of Medicine, Physician, National Pancreas Transplant Unit
Westmead Hospital

(CID) Associate Professor Carmel Hawley

Weekly Load (average hours/week)

Teaching	Clinical	NHMRC Research	Other Research
2	20	16	4

Administrative Responsibilities (average hours/week)

Administrative Commitment: 10
Responsibilities: Chair, Operations Secretariat, Australasian Kidney Trials Network, Clinical Director, Haemodialysis Unit, Princess Alexandra Hospital

(CIE) Professor Stephen McDonald

Weekly Load (average hours/week)

Teaching	Clinical	NHMRC Research	Other Research
2	25	2	10

Administrative Responsibilities (average hours/week)

Administrative Commitment: 6
Responsibilities: Clinical director, Country renal services South AUstralia, Supervisor, haemodialysis, Queen Elizabeth Hospital
