# Supplementary material for: Multi-center, pragmatic, cluster-randomized, controlled trial of standardized peritoneal dialysis (PD) training versus usual care on PD-related infections (the TEACH-PD trial): trial protocol
Source: Trials. 2023 Nov 14;24:730. doi: 10.1186/s13063-023-07715-0 (PMC10647147; doi:10.1186/s13063-023-07715-0)
Supplement: Supplementary file 2 — Additional file 2. Funding documents. [file Funding_AmgenR1.pdf › Funding_AmgenR1.pdf]

DocuSign Envelope ID: B4BC0CD0-47CF-4A78-8E01-ABE11E6B29A5

AMGEN

Amgen Australia Pty Ltd
A.B.N. 31 051 057 428

Head Office:
Level 7, 123 Epping Rd
NORTH RYDE NSW 2113
(PO Box 410
NORTH RYDE NSW 1670)
Tel. + 61 2 9870 1333
Fax +61 2 9870 1344

Medical Information:1800 803 638

27TH FEBRUARY 2019

Neil Boudville
University of Western Australia
35 Stirling Highway
Nedlands
WA 6009

Dear Professor Neil Boudville

Re: TEACH-PD

I am writing this letter ("Letter Agreement") to memorialize the terms upon which Amgen Australia Pty Ltd ("Amgen") wishes to provide support to **University of Western Australia** ("Recipient"). Amgen and Recipient may be referred to individually as a "Party" and collectively as the "Parties".

1. Amgen's Obligations.

Supported Activity. Amgen will make a grant to Recipient in the amount of **AUD50,000.00** (the "Donation") for the activity described in Annex 1 to this Agreement (the "Supported Activity"). Recipient shall invoice Amgen for the Donation on signing of this Letter Agreement and Amgen shall pay any and all undisputed invoices in accordance with the following:

- If Recipient has confirmed in writing to Amgen that Recipient is not required to hold an Australian Business Number ("ABN"), Amgen will make payment within thirty (30) days following receipt of invoice; OR

- If Recipient holds an ABN and is registered for GST (i.e. the supply of Services is a GST taxable supply), Recipient must issue Amgen a tax invoice for the Donation (net of GST). Amgen will make payment to recipient within thirty (30) days of receipt of valid tax invoice; OR

- If Recipient holds an ABN but is not required to be registered for GST, Recipient must issue Amgen an invoice for the Donation and the invoice must state Recipient is not registered for GST and is not required to be registered under GST law. Amgen will make payment to Recipient within thirty (30) days of receipt of a valid invoice.

Failure to make the confirmations required above, if applicable, will require Amgen to withhold tax from the amount payable to Recipient hereunder.

2. Recipient's Obligations.

(a) Restrictions on Use of Donation. Recipient will use the Donation solely for the Supported Activity. No part of the Donation may be paid, granted or distributed to any other organization or individual, other than to pay reasonable compensation for items and services provided to Recipient in connection with the Supported Activity.

(b) Use of logos. The Donation is made on the condition that Amgen's support shall be clearly acknowledged; this may be achieved through references in documentation related to the Supported Activity as follows: "Supported by Amgen" and/or the Amgen logo appearing on the Recipient's website with a link to www.amgen.com.au through the display of the Amgen logo. Except as provided herein, Recipient undertakes not to use Amgen name nor any trademark or other distinctive signs belonging to the Amgen group in any statements or public announcements without Amgen's prior written consent. Amgen shall have the right to use, copy and publish the name, trademark, or logo of the Recipient in order to comply with transparency reporting requirements to which it may be subject.

(c) Records. Recipient will maintain records of receipts and expenditures related to the Donation and the Supported Activity and as required by any applicable laws. Recipient's records required under this section must be adequate to determine whether Recipient used the Donation in compliance with this Letter Agreement. Recipient will make such records available to Amgen at such times that Amgen may request in its reasonable discretion during the term of this Letter Agreement and for five (5) years after the date of this Letter Agreement.

(d) Amgen is providing the Donation for the Supported Activity in accordance with **Edition 18**. Recipient and Amgen acknowledge and agree that the Donation is not intended, directly or indirectly, to compensate Recipient or Recipient Personnel for purchasing, ordering, prescribing, using or recommending Amgen's products or services, and neither Recipient nor any of the Recipient Personnel is required to purchase, use, prescribe, order, recommend, promote or advertise Amgen's products or services as a condition of this Letter Agreement. If at any time during the term of this Letter Agreement, Recipient's receipt of any amount under this Letter Agreement violates any statute, regulation, or a related judicial or government agency interpretation thereof, Amgen has no obligation to provide a Donation any higher than what is permitted, if any, under such statute, regulation or related interpretation. Recipient will, and will cause its employees, other agents and contractors (collectively, "Recipient Personnel") to conduct the Supported Activity and use the Donation in accordance with all local laws applicable to Recipient.

(e) Recipient represents, warrants and covenants, as of the date of this Letter Agreement to and through the expiration or termination of this Letter Agreement, (1) that Recipient, and, to the best of its knowledge, Recipient's owners, directors, officers, employees, or any agent, representative, subcontractor or other third party acting for or on Recipient's behalf (collectively, "Representatives"), shall not, directly or indirectly, offer, pay, promise to pay, or authorize such offer, promise or payment, of anything of value, to any individual or entity for the purposes of obtaining or retaining business or any improper advantage in connection with this Letter Agreement, or that would otherwise violate any applicable laws, rules and regulations

concerning or relating to public or commercial bribery or corruption ("Anti-Corruption Laws"), (2) that Recipient's books, accounts, records and invoices related to this Letter Agreement or related to any work conducted for or on behalf of Amgen are and will be complete and accurate and (3) that Amgen may terminate this Letter Agreement (a) if Recipient or Recipient's Representatives fails to comply with the Anti-Corruption Laws or with this provision, or (b) if Amgen has a good faith belief that Recipient or Recipient's Representatives has violated, intends to violate, or has caused a violation of the Anti-Corruption Laws. If Amgen requires that Recipient complete a compliance certification, Amgen may also terminate this Letter Agreement if Recipient (1) fails to complete a compliance certification, (2) fails to complete it truthfully and accurately, or (3) fails to comply with the terms of that certification.

3. Termination. Amgen may terminate this Letter Agreement immediately upon written notice to Recipient of a material breach of this Letter Agreement by Recipient or any Recipient Personnel which is not cured to the reasonable satisfaction of Amgen within thirty (30) days of Recipient's receipt of a notice describing the breach.

4. Disclosure. Notwithstanding anything to the contrary in this Letter Agreement, Recipient acknowledges and agrees that, to the extent required or necessary to comply with applicable laws and codes of practice on disclosure obligations, Amgen shall have the right to publicly disclose information regarding Recipient and this Letter Agreement and that information may include without limitation the following: (i) amounts, dates, and description of the Donation; (ii) Recipient's name, address; and (iii) a description of the Supported Activity.

5. Entire Agreement; Amendment; Waiver. This Letter Agreement, including any exhibits, is the entire agreement between Amgen and Recipient regarding the subject matter hereof. This Letter Agreement supersedes all prior or contemporaneous discussions, representations, correspondence, and agreements, oral or written, between the Parties with respect to the subject matter hereof. Any amendments or modifications to this Letter Agreement must be in writing and signed by both Parties. None of the provisions of this Letter Agreement shall be considered waived by either Party except when such waiver is given in writing.

6. Governing Law. This Letter Agreement will be governed by Australian law and the parties submit to the nonexclusive jurisdiction of the courts of New South Wales.

As part of Amgen's on-going commitment to the support of medical education and high-quality patient care we appreciate the opportunity to help you accomplish the Supported Activity. If the terms of the Letter Agreement are acceptable, please indicate your acceptance by signing two copies of it, faxing a copy immediately and returning one original to Amgen by post. If I can be of any assistance to you, please do not hesitate to contact me at the above address.

Yours sincerely,

AMGEN AUSTRALIA PTY LTD

DocuSigned by:
Ana Svensson
55EAD604AF3448C...

Name: Ana Svensson

Title: Medical Director

Acknowledgement and Agreement:

I have read this Letter Agreement and agree to be bound by its terms and conditions.

Signature: DocuSigned by: [signature] B3D9E74F96F8439...

Name: Neil Boudville

Title: Professor of Renal Medicine

Date: 05-Mar-2019

Annex 1
Supported Activity

Name of Activity	The TEACH-PD study
Type of Activity/Objectives	TEACH-PD is a registry-based, pragmatic, cluster-RCT evaluating whether implementation of standardised training modules based on International Society for Peritoneal Dialysis (ISPD) guidelines targeting both PD trainers and patients reduces the composite end-point of exit site infection, tunnel infection and peritonitis in incident PD patients compared to current standard of care.
Time Frame	1 February 2019 – 31 December 2023
