# Supplementary material for: Multi-center, pragmatic, cluster-randomized, controlled trial of standardized peritoneal dialysis (PD) training versus usual care on PD-related infections (the TEACH-PD trial): trial protocol
Source: Trials. 2023 Nov 14;24:730. doi: 10.1186/s13063-023-07715-0 (PMC10647147; doi:10.1186/s13063-023-07715-0)
Supplement: Supplementary file 2 — Additional file 2. Funding documents. [file Funding_Baxter CECR1.pdf › Funding_Baxter CECR1.pdf]

Baxter

September 26, 2018

Prof. David Johnson
Princess Alexandria Hospital
Department of Nephrology ARTS Building
199 Ipswich Road
Woolloongabba
QLD 4102
Australia

Subject: Grant Application for Baxter Renal Care

Dear Professor Johnson:

Congratulations!

On behalf of Dr. Angelito Bernardo, I am delighted to inform you that the Global Scientific Review Council has recommended that your grant application, "*Targeted Education Approach to Improve Pertioneal Dialysis Outcomes (TEACH-PD)*, be funded.

Total funding award to be provided by Baxter in the amount of $150,000 AUD

Our contract associate will forward a Research Agreement including grant payment schedule for review and approval. The negotiation of the Research Agreement must be completed within 90 days from the Institution's receipt of the initial draft Agreement. Failure to meet this deadline may result in withdrawal by Baxter of the grant award. Also, please provide a copy of your protocol as a Word document to my attention at michelle_duddington@baxter.com

Thank you for submitting your application to our research grant program and again, congratulations on your grant award. Please do not hesitate to contact me if you have any questions.

Sincerely,

Kelly A. Machak

Kelly A. Machak
Senior Grants Program Manager
Medical Affairs Operations
Baxter Healthcare Corporation

CC: Angelito Bernardo
Mark Marshall
Michelle Duddington
Victor Carey
