# Supplementary material for: Multi-center, pragmatic, cluster-randomized, controlled trial of standardized peritoneal dialysis (PD) training versus usual care on PD-related infections (the TEACH-PD trial): trial protocol
Source: Trials. 2023 Nov 14;24:730. doi: 10.1186/s13063-023-07715-0 (PMC10647147; doi:10.1186/s13063-023-07715-0)
Supplement: Supplementary file 2 — Additional file 2. Funding documents. [file Funding_ISPD Research GrantR1.pdf › Funding_ISPD Research GrantR1.pdf]

FW: Result: call for ISPD Research Proposals 2017

Josephine Chow

Sent: 17 March 2018 10:37 AM

To: Carmel Hawley [Carmel.Hawley@health.qld.gov.au]; Elaine Pascoe [e.pascoe@uq.edu.au]; Yeoungjee Cho [Yeoungjee.Cho@health.qld.gov.au]; Matthew Jose (Matthew.Jose@utas.edu.au) (Matthew.Jose@utas.edu.au); David Johnson [David.Johnson2@health.qld.gov.au]; Teach PD AKTN [teachpd@uq.edu.au]; Neil Boudville (neil.boudville@uwa.edu.au); Laura Robison [l.robison@uq.edu.au]; Donna Reidlinger [d.reidlinger@uq.edu.au]

Dear All

Congratulations to all. Only the third trial.

Dear Donna

Would you mind emailing me the name and address for the cheque or would you like me to make other arrangement?

Jo

From: Miguel Gallardo [miguel.gallardo@ispd.org]
Sent: 16 March 2018 8:34 PM
To: Josephine Chow
Cc: David Johnson; Anjali Saxena
Subject: Result: call for ISPD Research Proposals 2017

Message sent on behalf of David Johnson, Chair of the ISPD International Studies Committee

Dear Dr Chow,

Thank you very much for your recent application for ISPD International Studies Committee funding of your research proposal entitled *Targeted Education ApproaCH to improve Peritoneal Dialysis outcomes (TEACH-PD Trial)*.

I am pleased to announce that your application was successful, and that your group has been awarded USD $10,000.00.

The $10,000.00 will be awarded by way of cheque. That being the case, may I please ask to confirm the **name to which the cheque should be drawn** and the **address to which the cheque should be sent** to Dr Anjali Saxena, ISPD Treasurer, to arrange for payment. I have taken the liberty of CCing Dr Saxena into this email chain so you have her details.
The formal announcement will be done next Monday on our website and social media.
On behalf of the International Society for Peritoneal Dialysis (ISPD), I wish you every success with your exciting and very important research.

Yours sincerely,

Prof David Johnson
Chair, International Studies Committee
ISPD

Best regards,
Miguel Gallardo
ISPD Society Coordinator
InternationalSociety for Peritoneal Dialysis
Rue des Fabriques 1
B-1000 Brussels - Belgium
Phone: +32 2 808 04 20 | Fax +32 2 808 44 54
Follow us on Twitter & Facebook

17th Congress of the International Society for Peritoneal Dialysis
VANCOUVER, CANADA | MAY 5-8, 2018

[ISPD Vancouver 2018, a different kind of meeting]
