# Supplementary material for: Multi-center, pragmatic, cluster-randomized, controlled trial of standardized peritoneal dialysis (PD) training versus usual care on PD-related infections (the TEACH-PD trial): trial protocol
Source: Trials. 2023 Nov 14;24:730. doi: 10.1186/s13063-023-07715-0 (PMC10647147; doi:10.1186/s13063-023-07715-0)
Supplement: Supplementary file 2 — Additional file 2. Funding documents. [file Funding_SWS Small Grant SchemeR1.pdf › Funding_SWS Small Grant SchemeR1.pdf]

SOUTH WEST SYDNEY RESEARCH

Professor Josephine Chow
Director, Liverpool Renal Clinical Research Centre
Clinical Stream Office
Liverpool Hospital
Don Everett Building
Locked Bag 7103
Liverpool 2170 NSW
E: Josephine.Chow@health.nsw.gov.au

22nd June 2018

Dear ~~Professor~~ Josephine Chow,

It is with great pleasure that I advise that your recent application to the South West Sydney Research (SWSR) Small Grant Scheme 2018 has been successful.

Your project titled "Targeted Education ApproaCH to improve Peritoneal Dialysis outcomes (TEACH-PD) – Digital Patient Training Manuals development and validation" has been awarded $20,000 + GST under the following conditions:

- Funds must be spent in accordance with the Project Budget
- Funds to be expended by 30 June 2019 (with return of any unspent funds)
- A final report on the outcomes as they relate to the Project Plan and funds acquittal must be submitted to info@southwestsydneyresearch.org.au by 31 July 2019
- The funding contribution of South West Sydney Research (SWSR) must be acknowledged on any output arising from the awarded grant

Grant funds will be paid following receipt of a valid tax invoice and a signed copy of this letter indicating your acceptance of the grant offer and its conditions.

Congratulations on your success and I look forward to seeing the outcomes of your project.

Yours sincerely,

Michael

Professor Michael Barton OAM
Chair, South West Sydney Research

CC: Luisa Ford (Ingham Institute)

Acceptance:

**FULL NAME**	Josephine Chow
**SIGNATURE**	[signature]
**DATE**	7/7/18
