# Supplementary material for: Multi-center, pragmatic, cluster-randomized, controlled trial of standardized peritoneal dialysis (PD) training versus usual care on PD-related infections (the TEACH-PD trial): trial protocol
Source: Trials. 2023 Nov 14;24:730. doi: 10.1186/s13063-023-07715-0 (PMC10647147; doi:10.1186/s13063-023-07715-0)
Supplement: Supplementary file 2 — Additional file 2. Funding documents. [file Funding_Translational Research InstituteR1.pdf › Funding_Translational Research InstituteR1.pdf]

TRANSLATIONAL RESEARCH INSTITUTE
AUSTRALIA

December 2017

Magid Fahim
Email: Magid.Fahim@health.qld.gov.au

Dear Dr Fahim

It is my pleasure to inform you that your application ***Targeted Education Approach to improve Peritoneal Dialysis*** has been successful in obtaining one of two 2017 TRI Development Awards.

The Judges, members of the TRI Personal Development and Talent Management Committee, deemed that your project met all of the criteria and should receive an Award.

The $10,000 in Award funding is available from January 2018. The Award funds will be administered through your institution. Please arrange for your invoice for $10,000 plus GST to be forwarded to TRI Accounts at Accounts@tri.edu.au.

If you have any questions, please contact Louise Morland, TRI Director, Communications and Marketing, tel: 3443 7744 or email: louise.morland@tri.edu.au.

Congratulations once again and all the best in achieving the desired outcomes for your project.

Kind Regards

Professor Carolyn Mountford D.Phil
CEO and Director of Research
Translational Research Institute Pty Ltd
