# Supplementary material for: Multi-center, pragmatic, cluster-randomized, controlled trial of standardized peritoneal dialysis (PD) training versus usual care on PD-related infections (the TEACH-PD trial): trial protocol
Source: Trials. 2023 Nov 14;24:730. doi: 10.1186/s13063-023-07715-0 (PMC10647147; doi:10.1186/s13063-023-07715-0)
Supplement: Supplementary file 3 — Additional file 3. Ethics Approval (Australia). [file 13063_2023_7715_MOESM3_ESM.pdf]

Research and Ethics Office
Leadership · Quality · Governance
Locked Bag 7103, LIVERPOOL BC, NSW, 1871
Phone: 02 8738 8304
Facsimile: 02 8738 8310
https://www.swslhd.health.nsw.gov.au/ethics/default.html

South Western Sydney Local Health District acknowledges the traditional owners of this land.

8 March 2019

Professor Josephine Chow
Liverpool Hospital
Locked Bag 7103
LIVERPOOL BC NSW 1871

*****THIS LETTER CONSTITUTES ETHICAL APPROVAL ONLY. THIS RESEARCH PROJECT MUST NOT COMMENCE AT A SITE UNTIL SEPARATE AUTHORISATION FROM THE CHIEF EXECUTIVE OR DELEGATE OF THAT SITE HAS BEEN OBTAINED. ******

Dear Professor Chow,

Project Title: **TEACH-PD CRCT Study**
HREC Reference: **HREC/19/LPOOL/32**
Local Project Number: **HE19/15**

Thank you for your response dated 21 February 2019 to our request for further information dated 18 February 2019. This Human Research Ethics Committee is constituted and operates in accordance with the National Health and Medical Research Council's *National Statement on Ethical Conduct in Research Involving Humans* and the *CPMP/ICH Note for Guidance on Good Clinical Practice*.

I am pleased to advise that the Committee has granted ethical approval of the above project.

The following documentation has been reviewed and approved:

Document	Version	Date
Human Research Ethics Application	AU/1/970A317	31.01.2019
Protocol	1.0	30.01.2019
Australian Specific Appendix	1.0	30.01.2019
Protocol – Australia	1.0	30.01.2019
MASTER Participant Information Sheet/Consent Form – Part A	1.0	30.01.2019
MASTER Participant Information Sheet/Consent Form – Part B	1.0	30.01.2019
Queensland Health Memorandum	N/A	05.09.2018
Questionnaire – EQ-5D-5L	1.0	2009
Ability to Participate in Social Roles and Activities – Short Form 6A	2.0	23.06.2016
Australasian Kidney Trials Network - Scientific Committee Meeting Minutes	N/A	11.05.2017

Please ensure for all future documents submitted for review include a document version number, document date and page numbering.

Monitoring Requirements:
(National Statement Chapters 2.1 and 5.5)

- The Committee has classified this project as:

Low Risk

- Monitoring required for this study will be:

- Submission of Annual Progress Reports with the first report due **8 March 2020 and annually thereafter for the duration of the approval period**

Approval has been granted for the following site(s):

- Liverpool Hospital
- Cairns Hospital
- Gold Coast University Hospital
- Princess Alexandra Hospital
- Sunshine Coast University Hospital
- The Townsville Hospital
- Toowomba Hospital
- Logan Hospital

Please note the following conditions of approval:

1. The Principal Investigator will immediately report anything which might warrant review of ethical approval of the project in the specified format, including:
   - any serious or unexpected adverse events; and
   - unforeseen events that might affect continued ethical acceptability of the project.

2. The Principal Investigator will report proposed changes to the research protocol, conduct of the research, or length of HREC approval to the HREC in the specified format, for review. For multi-centre studies, the Chief Investigator should submit to the Lead HREC and then send the amendment approval letter to the investigators at each sites so that they can notify their Research Governance Officer.

3. The Principal Investigator will inform the HREC, giving reasons, if the project is discontinued before the expected date of completion.

4. The Principal Investigator will provide an annual report to the HREC and at completion of the study in the specified format.

5. The Principal Investigator must reassure participants about confidentiality of the data.

6. Proposed changes to the personnel involved in the study are submitted to the HREC accompanied by a CV where applicable.

7. The Principal Investigator is responsible for ensuring the research project is conducted in line with relevant NSW Health, South Western Sydney Local Health District and Hospital policies available from: https://www.swslhd.health.nsw.gov.au/ethics/default.html

HREC approval is valid for (5) years. If the study is ongoing at the conclusion of the five year approval period, a full resubmission may be required. Ethics approval will continue during the re-approval process.

The South Western Sydney Local Health District Human Research Ethics Committee has been accredited by the NSW Ministry of Health to provide single ethical and scientific review of research proposals conducted within the NSW public health system and Victorian and Queensland Public Health Organisations participating in the Mutual Acceptance Scheme.

You are reminded that this letter constitutes ethical approval only. This research project must not commence at a site until separate authorisation from the Chief Executive or delegate of that site has been obtained. It is your responsibility to forward a copy of this letter together with any approved documents as enumerated above, to all site investigators for submission to the site's Research Governance Officer.

Should you have any queries about your project please contact **Jessica Grundy** on the telephone number 8738 8304. The HREC Terms of Reference, Standard Operating Procedures, membership and standard forms are available from the SWSLHD website: *https://www.swslhd.health.nsw.gov.au/ethics/default.html*

Please quote the Local HREC reference **HE19/015** in all correspondence. The HREC wishes you every success in your research.

Yours faithfully

Jessica Grundy
on behalf of
Professor Jeremy Wilson
Chairperson, SWSLHD Human Research Ethics Committee

This HREC is constituted and operates in accordance with the National Health and Medical Research Council's (NHMRC) *National Statement on Ethical Conduct in Human Research (2007).* The processes used by this HREC to review multi-centre research proposals have been certified by the National Health and Medical Research Council.
