# Supplementary material for: Multi-center, pragmatic, cluster-randomized, controlled trial of standardized peritoneal dialysis (PD) training versus usual care on PD-related infections (the TEACH-PD trial): trial protocol
Source: Trials. 2023 Nov 14;24:730. doi: 10.1186/s13063-023-07715-0 (PMC10647147; doi:10.1186/s13063-023-07715-0)
Supplement: Supplementary file 4 — Additional file 4. Ethics Approval (New Zealand). [file 13063_2023_7715_MOESM4_ESM.pdf]

Health and Disability Ethics Committees
Ministry of Health
133 Molesworth Street
PO Box 5013
Wellington
6011

0800 4 ETHICS
hdecs@health.govt.nz

23 September 2019

Professor Suetonia Palmer
Department of Medicine
University of Otago Christchurch
2 Riccarton Ave
Christchurch 8140

Dear Professor Palmer,

Re:	**Ethics ref:**	**19/NTB/133**
	Study title:	A pragmatic, registry-based, international, cluster-randomised controlledtrial examining the use of TEACH-PD training modules for incident PD patients versus existing practices on the rate of PD-related infections.

I am pleased to advise that this application has been *approved* by the Northern B Health and Disability Ethics Committee. This decision was made through the HDEC-Expedited Review pathway.

Conditions of HDEC approval

HDEC approval for this study is subject to the following conditions being met prior to the commencement of the study in New Zealand. It is your responsibility, and that of the study's sponsor, to ensure that these conditions are met. No further review by the Northern B Health and Disability Ethics Committee is required.

Standard conditions:

1. Before the study commences at *any* locality in New Zealand, all relevant regulatory approvals must be obtained.

2. Before the study commences at *any* locality in New Zealand, it must be registered in a clinical trials registry. This should be a WHO-approved registry (such as the Australia New Zealand Clinical Trials Registry, www.anzctr.org.au) or https://clinicaltrials.gov/.

3. Before the study commences at *each given* locality in New Zealand, it must be authorised by that locality in Online Forms. Locality authorisation confirms that the locality is suitable for the safe and effective conduct of the study, and that local research governance issues have been addressed.

Please refer to the *Standard Operating Procedures for Health and Disability Ethics Committees* (available on www.ethics.health.govt.nz) for HDEC requirements relating to amendments and other post-approval processes.

Your next progress report is due by 23 September 2020.

Participant access to ACC

The Northern B Health and Disability Ethics Committee is satisfied that your study is not a clinical trial that is to be conducted principally for the benefit of the manufacturer or distributor of the medicine or item being trialled. Participants injured as a result of treatment received as part of your study may therefore be eligible for publicly-funded compensation through the Accident Compensation Corporation (ACC).

Please don't hesitate to contact the HDEC secretariat for further information. We wish you all the best for your study.

Yours sincerely,

Ms Kate O'Connor
Chairperson
Northern B Health and Disability Ethics Committee

Encl: appendix A: documents submitted
appendix B: statement of compliance and list of members

Appendix A
Documents submitted

Document	*Version*	*Date*
CV for CI: CV for Co-ordinating investigator	V1.0	03 June 2019
Evidence of scientific review: AKTN Scientific Committee review 11May2017	1	11 May 2017
PIS/CF: TEACH-PD NZ PICF A	V1.1	13 September 2019
PIS/CF: TEACH-PD NZ PICF B	V1.1	13 September 2019
Evidence of scientific review: HRC Reviewer scientific assessments March 2019	2019	03 March 2019
CVs for other Investigators: CV for Other investigator_Chembo	V1.0	29 May 2019
Survey/questionnaire: EQ-5D-5L NZ English version	V1.0	03 June 2019
CVs for other Investigators: CV for Other Investigator_Hayat	v1.0	29 May 2019
CVs for other Investigators: CV for Other investigator_Reynolds	V1.0	08 June 2019
CVs for other Investigators: CV for Other investigator_Maher	V1.0	11 June 2019
CVs for other Investigators: CV for Other investigator_Kendrick-Jones	v1.0	08 June 2019
CVs for other Investigators: CV for Other investigator_Saweirs	v1.0	29 May 2019
CVs for other Investigators: CV for Other investigator_Putt	v1.0	04 June 2019
CVs for other Investigators: CV for Other investigator_Nguyen	v1.0	11 June 2019
CVs for other Investigators: CV for Other investigator_Tan	v1.0	19 September 2018
Evidence of CI indemnity	18-19	05 December 2018
Evidence of CI indemnity	18-19	05 December 2018
Protocol: TEACH-PD CRCT Protocol New Zealand Specific Appendix	V1.1	20 September 2019
Health Research Council of NZ funding decision	Letter	31 May 2019
Evidence of consultation with Kaitohutohu Rangahau Maori at University of Otago Christchurch	V1.0	06 November 2018
TEACH-PD trial infographic New Zealand	V1.0	12 June 2019
Covering Letter: Covering letter TEACH-PD	V1.0	18 August 2019
CVs for other Investigators: CV for Other investigator_Aday	V1.0	08 July 2019
Evidence of sponsor insurance	18-19	05 December 2018
Evidence of sponsor insurance	18-19	05 December 2018
Protocol: TEACH-PD CRCT Protocol	V1.0	30 January 2019
Application		20 August 2019
PIS/CF: TEACH-PD NZ PICF B_tracked	V1.1	13 September 2019
PIS/CF: TEACH-PD NZ PICF A_tracked	V1.1	13 September 2019
Response to Committee	1	20 September 2019
Response to Request for Further Information		

Appendix B
Statement of compliance and list of members

Statement of compliance

The Northern B Health and Disability Ethics Committee:

- is constituted in accordance with its Terms of Reference
- operates in accordance with the *Standard Operating Procedures for Health and Disability Ethics Committees*, and with the principles of international good clinical practice (GCP)
- is approved by the Health Research Council of New Zealand's Ethics Committee for the purposes of section 25(1)(c) of the Health Research Council Act 1990
- is registered (number 00008715) with the US Department of Health and Human ervices' Office for Human Research Protection (OHRP).

List of members

Name	*Category*	*Appointed*	*Term Expires*
Mr John Hancock	Lay (the law)	14/12/2015	14/12/2018
Dr Nora Lynch	Non-lay (health/disability service provision)	19/03/2015	19/03/2022
Miss Tangihaere Macfarlane	Lay (consumer/community perspectives)	20/05/2017	20/05/2020
Mrs Kate O'Connor	Lay (ethical/moral reasoning)	14/12/2015	14/12/2018
Mrs Stephanie Pollard	Non-lay (intervention studies)	01/07/2015	01/07/2018
Mrs Leesa Russell	Non-lay (intervention studies), Non-lay (observational studies)	14/12/2015	14/12/2018
Ms Susan Sherrard	Lay (consumer/community perspectives)	19/03/2019	19/03/2022
Mrs Jane Wylie	Non-lay (intervention studies)	20/05/2017	20/05/2020

Unless members resign, vacate or are removed from their office, every member of HDEC shall continue in office until their successor comes into office (HDEC Terms of Reference)

http://www.ethics.health.govt.nz
